# Universal Regulation of DNA Polymerase via Photocaged Primer Enables Light‐Start Isothermal Amplification on Demand

**DOI:** 10.1002/advs.202511245

**Published:** 2025-10-24

**Authors:** Min Qing, Yufan Qin, Zhijin Li, Xiufang Yu, Jianbo Wang, Xiaoqiong Liu, Xin Chen, Chao Yu

**Affiliations:** ^1^ Chongqing Key Laboratory for Pharmaceutical Metabolism Research College of Pharmacy Chongqing Medical University Chongqing 400016 P. R. China; ^2^ Department of Infectious Diseases the First Affiliated Hospital of Chongqing Medical University Chongqing 400016 P. R. China

**Keywords:** in vitro molecular diagnosis, light‐start recombinase polymerase amplification, photoactivatable DNA polymerase

## Abstract

The development of DNA polymerases with controllable functions, such as hot‐start or light‐start variants, allows on‐demand activation of enzymatic activity and therefore improves reaction specificity. However, the fine control of DNA polymerase activity faces challenges to accuracy and flexibility. Here, a simple and general photoactivatable approach for the temporal regulation of DNA polymerase activity, and thus, light‐start isothermal amplification on demand is engineered. This approach leverages photocaged primer with light‐sensitive 6‐nitropiperonyloxymethyl to temporarily inhibit the conformational change of DNA polymerase until reactivation by 365 nm UV. The mechanism through biochemical assays and molecular dynamic simulations is elucidated, and validate the temporal precision using droplet digital technology. The light‐start recombinase polymerase amplification (Light‐start RPA) and demostrates that it improves reaction specificity and provides superior temporal control, thereby integrating better into digital droplet workflows is further developed. Moreover, Light‐start RPA offers sensitivity, specificity, and multiplexing capability for pathogen detection. Notably, both RT‐Light‐start RPA and Light‐start RPA‐based lateral flow assays demonstrate 100% sensitivity and specificity for influenza A virus detection within 20 min. Overall, this photocaged primer approach not only expands the conditional control toolbox of DNA polymerase activity, but also provides a versatile and promising framework for molecular diagnostics and microfluidics.

## Graphical abstract

A simple and general photoactivatable approach is developed to regulate DNA polymerase activity, enabling light‐start isothermal amplification on demand. The molecular dynamics simulations reveals that the photocaged primer blocks DNA polymerase activity by inhibiting the conformational change of the polymerase. Moreover, the proposed light‐start recombinase polymerase amplification (Light‐start RPA) improves reaction specificity and provides superior temporal control.

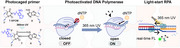

## 1 Introduction

DNA polymerase is a powerful tool for rapid DNA synthesis, catalyzing the addition of deoxyribonucleotides to the 3′‐end of a primer to generate a complementary strand to a DNA template.^[^ ^1^, ^2^ ^]^ Leveraging this capability, various methods have been developed to detect trace amounts of nucleic acids in complex biological samples. A classical example is the polymerase chain reaction (PCR), which has been widely applied in fields ranging from molecular diagnostics to synthetic biology.^[^ ^3^, ^4^, ^5^, ^6^ ^]^ The biotechnological significance of DNA polymerase has motivated efforts to regulate its function, enabling on‐demand activation of enzymatic activity while minimizing undesired side effects.^[^ ^7^, ^8^, ^9^ ^]^ Although factors such as temperature, target sequence, and ion concentration are known to influence polymerase activity,^[^ ^10^, ^11^ ^]^ the precise control of DNA synthesis remains insufficiently understood. The ability to control the function of DNA polymerase will be the foundation of advance this technology toward biomedical applications and beyond.

Attempts to regulate DNA polymerase functions have involved hot‐start or light‐start variants. Hot‐start approaches employ engineered aptamers or antibodies to inhibit polymerase activity at lower temperatures, allowing activation only upon reaching a defined elevated temperature.^[^ ^12^, ^13^ ^]^ The hot‐start DNA polymerases are arguably one of the most successful applications of enzymes with controllable functions to biotechnology. However, the development of antibodies and aptamers specific for a given polymerase requires laborious optimization and time‐consuming library screening.^[^ ^14^, ^15^ ^]^ Moreover, the mandatory heating step renders this approach incompatible with mesophilic DNA polymerases, such as *Bsu* DNA polymerase, Klenow fragment, and phi29 DNA polymerase, which operate optimally at 30–37 °C.

Regulation of DNA polymerase function with light provides control over biological processes with unprecedented temporal resolution.^[^ ^16^, ^17^ ^]^ Methods have been developed to achieve light‐control of DNA polymerase activity, including the incorporation of a light‐responsive ortho‐nitrobenzyl caging group into the tyrosine residue of *Taq* polymerase, and the use of covalently bound oligonucleotides to block the DNA polymerase through substrate competition.^[^ ^7^, ^18^, ^19^ ^]^ The incorporation of photosensitive unnatural amino acids via protein engineering allows for precise light activation of specific enzymatic functions without the need for hot‐start aptamers or antibodies blockage.^[^ ^20^, ^21^ ^]^ However, the development of photoactivatable enzymes requires detailed structural and mechanistic insights into the enzyme, as well as a meticulous majorization of the conditions. Alternatively, photosensitive oligonucleotides were fused to DNA polymerase via click reaction or nonconvalent binding to effectively inactivate the enzyme, while UV irradiation restores its DNA polymerization activity.^[^ ^7^ ^]^ Similar to the protein engineering strategy, the later also needs enzyme site‐specific functionalization with unnatural amino acid or G‐quadruplex‐binding protein. Importantly, because oligonucleotides enter the enzyme's cleft or compete for access to the active site and are not easily removed, this approach may result in low photoactivation efficiency. Overall, current approaches are dedicated to modifications of DNA polymerase, whereas the desire for simple and general strategies still need to be addressed.

We anticipate that conditional control of primers will not only circumvent the need for protein engineering, but will also provide a simple and flexible way to regulating DNA polymerase activity. Although previous studies have employed photocaged primers to achieve light‐start PCR,^[^ ^22^ ^]^ the requirement for multiple light‐sensitive groups modification faces challenge to generalizability. For example, changing the detection target necessitates re‐screening both the position and number of caging groups. This multi‐modification is both cumbersome and costly, and requires longer activation time. Moreover, the conditional regulation of DNA polymerase activity through photocaged primers has not been systematically explored, including the control of multiple enzymatic activities, temporal regulation, and mechanistic insights. The isothermal amplification operates at moderate temperatures using mesophilic DNA polymerases, and the uncontrolled initiation is prone to generating nonspecific products. It remains an open question whether photocaged primers can regulate DNA polymerase activity to suppress premature amplification during isothermal nucleic acid amplification in miniaturized compartments, such as droplets or microchambers. Therefore, we engineer a simple and general photocaged primer approach for precise‐regulation of DNA polymerase activity and thus light‐start isothermal nucleic acid amplification on demand. This approach leverages photocaged primer modified with light‐sensitive 6‐nitropiperonyloxymethyl (6‐NPOM) at the 3′‐end to temporarily inhibit the conformational change of DNA polymerase until reactivation by 365 nm UV. The biochemical assays and molecular dynamics simulations reveal that the photocaged primer blocks DNA polymerase activity by inhibiting the conformational change of the polymerase. We also demonstrate the temporal precision by combining droplet digital technology. In addition, we reveal a novel application of photoactivatable DNA polymerases in recombinase polymerase amplification (RPA). In contrast to conventional RPA, the light‐start recombinase polymerase amplification (Light‐start RPA), which relies on light rather than magnesium ions to initiate the reaction, improves reaction specificity by preventing premature nonspecific amplification prior to light activation. This approach provides superior temporal control to initiate amplification on demand, thereby integrating better into digital droplet workflows. We further demonstrate that Light‐start RPA offers sensitivity, specificity, and multiplexing capability for pathogen detection. Notably, both RT–Light‐start RPA and Light‐start RPA‐based lateral flow assays demonstrate 100% sensitivity and specificity for influenza A virus detection within 20 min. Overall, this photocaged primer approach is a flexible and universal alternative for precise‐regulation of DNA polymerase activity.

## 2 Results and Discussion

### 2.1 Design and Mechanistic Insights of Photocaged Primer‐Regulation of DNA Polymerase Activity

The photocaged primer is designed by substituting the thymine with light‐sensitive, 6‐nitropiperonyloxymethyl (6‐NPOM)‐caged thymine.^[^ ^23^ ^]^ Photo‐caging fully disrupts Watson−Crick hydrogen bonding, with rapid restoration of hybridization affinity upon photoactivation. Unlike previous studies where the photosensitive 6‐NPOM groups were randomly distributed on the primer or incorporated into the 5′‐end,^[^ ^17^, ^22^, ^24^ ^]^ this study highlights the effects and applications of 3′‐end modification. Primer−template complementation is essential for primer extension. Previous works have shown that the mismatch at the 3′‐end of the primer prevents DNA polymerase from initiating DNA synthesis.^[^ ^1^, ^25^ ^]^ We anticipate that modification of the 6‐NPOM‐caged nucleobases at the 3′‐end of the primers will block the Watson−Crick base pairing, thereby inhibiting polymerase‐mediated DNA synthesis. To investigate the effects of modified positions on the conditional control of DNA polymerase activity, two 6‐NPOM‐caged thymidines were incorporated into different positions of reverse primers and evaluated in quantitative PCR (qPCR) (Figure S1a, Supporting Information). Upon light activation, *Taq* DNA polymerase showed complete restoration of polymerization activity with all photocaged reverse primers (Figure S1b, Supporting Information). However, cycle threshold (Ct) value revealed differences in inhibitory effects among various photocaged reverse primers. In the absence of light activation, no amplification was observed when the 3′‐end was caged, whereas caging at the middle position caused delayed amplification. Notably, nonspecific amplification was observed when 6‐NPOM‐caged thymidines were positioned in close proximity to the 5′‐end of the primer (Figure S1c, Supporting Information). These results indicate that the mismatch at the 3′‐end of the primer has a greater inhibitory effect on the activity of *Taq* DNA polymerase. Furthermore, we evaluated the effect of the number of 3′‐end modifications on the conditional control of *Taq* DNA polymerase. A single‐base photocaging strategy was sufficient to effectively inhibit *Taq* DNA polymerase activity (Figure S2, Supporting Information). This 3′‐end photo‐caging strategy provides a simple and flexible design, effectively eliminating the need for optimization of modification positions.

**Figure 1:**
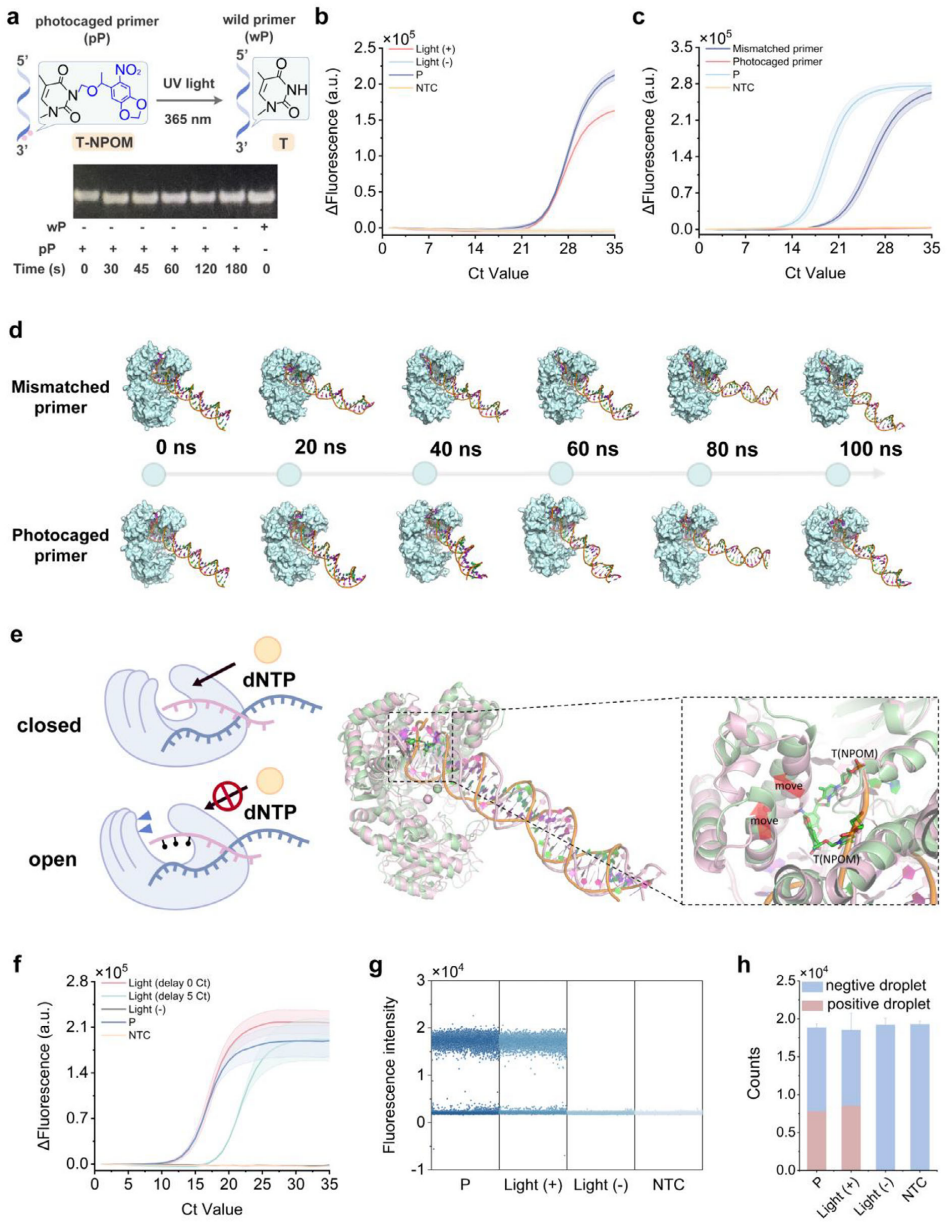
a) The photocaged reverse primer (EMP1‐RP2‐NPOM) was treated with 365 nm UV lamp (30 W) for varying durations and subsequently analyzed by PAGE. The wild reverse primer (EMP1‐RP2) was served as positive control. b) The feasibility analysis of photoactivated qPCR for amplifying EMP1 gene (1 pg µL^−1^) using wild forward primer (EMP1‐FP2) and photocaged reverse primer (EMP1‐RP2‐NPOM). c) The exonuclease resistance of photocaged primer against high‐fidelity DNA Polymerase. The qPCR experiments were conducted using wild forward primer (EMP1‐FP2) and either mismatched reverse primer (EMP1‐RP2‐M3) or photocaged reverse primer (EMP1‐RP2‐NPOM), without treatment with 365 nm UV lamp (30 W). d) The conformational changes of primer‐template‐protein complexes over a 100 ns timescale. e) Comparative analysis of mismatched primer‐template‐polymerase (pink) and photocaged primer‐template‐polymerase (green) overlays at the 100 ns. f) Time course of photoactivated PCR for amplifying CHIKV plasmid (1 ng µL^−1^) using wild forward primer (CHIKV‐FP3) and photocaged reverse primer (CHIKV‐RP1‐NPOM) g) Scatterplot of the feasibility of photoactivated ddPCR for amplifying the linear CHIKV plasmid (10 fg µL^−1^) using wild forward primer (CHIKV‐FP3) and photocaged reverse primer (CHIKV‐RP1‐NPOM). h) Quantification of positive and negative droplets in photoactivated ddPCR. "Light+" indicates that amplification reagent was subjected to light treatment with 365 nm UV lamp (30 W) for 50 s. "Light‐" indicates that the reaction was not treated with a 365 nm UV lamp. P represents the positive control group, using a plasmid template (EMP1 and CHIKV plasmid for qPCR, linear CHIKV plasmid for ddPCR) with wild forward and reverse primers (EMP1‐FP2 and EMP1‐RP2 for qPCR, CHIKV‐FP3 and CHIKV‐RP1 for qPCR, CHIKV‐FP3 and CHIKV‐RP1 for ddPCR) for conventional qPCR. NTC represents the blank control, using RNase‐free water instead of plasmid template. ΔFluorescence (a.u.) represents the difference between the fluorescence value at 35 Ct and the initial fluorescence value. Data are represented as mean ± standard error (*n* = 3 technical replicates).

Subsequently, polyacrylamide gel electrophoresis (PAGE) analysis showed that the shift position of the characteristic bands of the photocaged reverse primer was consistent with that of the wild reverse primer after exposure to 365 nm UV (**Figure** **1**a). Low‐resolution mass spectrometry demonstrated that the 6‐NPOM‐caged thymine in the photocaged reverse primer can be converted to normal thymine through light activation within 50 s (Figure S3, Supporting Information). Moreover, the qPCR assay revealed that the Ct value of the photoactivated qPCR was consistent with that of conventional qPCR (Figure 1b), and the melting curve further confirmed this result (Figure S4, Supporting Information). This underlines that photoactivation of DNA polymerase enables efficient recovery of its activity, which is comparable to that of hot‐start DNA polymerase. Additionally, the general applicability of the photocaged primer strategy was validated using four additional photocaged primers targeting the DENV, CHIKV, ZIKV, and H1N1 genes (Figures S5–S8, Supporting Information). The dose‑response experiments showed negligible impact on template integrity and amplification efficiency after UV exposure of 70 s (Figure S9, Supporting Information).

The hot‐start DNA polymerase strategy prevents nonspecific amplification by inhibiting its activity at low temperatures. To evaluate whether the photocaged primers could prevent nonspecific amplification, we designed a system containing primers with three complementary bases at their 3′‐ends, and the products of PCR were analyzed by agarose gel electrophoresis (Figure S10, Supporting Information). These primers generate clear primer dimer products in the presence of wild Taq DNA polymerase alone, and even in the presence of additional input EMP1 template. However, when using the photocaged reverse primer, only the desired target‐specific product is observed. These results demonstrate that photoactivated PCR prevents nonspecific primer dimer formation, similar to hot‐start DNA polymerases.

The *Pro Taq* HS DNA polymerase premix includes a high‐fidelity DNA polymerase possessing 3′−5′ exonuclease activity, enabling the correction of mismatched nucleobases. We further investigated whether the photocaged primer possesses exonuclease resistance (Figure 1c). The reverse primer containing mismatched nucleobases was corrected by high‐fidelity DNA polymerase, but showed slower extension kinetics compared to the fully matched primer. In contrast, the photocaged reverse primer remained stable without light activation and could not be extended by *Taq* DNA polymerase. The phi29 DNA polymerase possesses strong 3′−5′ exonuclease activity.^[^ ^26^ ^]^ To study exonuclease resistance of photocaged primer against phi29 DNA polymerase, we designed a primer degradation experiment (Figure S11, Supporting Information). Following a 20 min incubation with phi29 DNA polymerase, the wild primer was nearly completely degraded. In contrast, the relative intensity of the band of the photocaged primer remained above 80% after incubating with phi29 DNA polymerase for 40 min. These results suggest that the photocaged primer exhibits exonuclease resistance against high‐fidelity DNA polymerases but does not provide complete resistance against phi29 DNA polymerase. Future research can explore enhancing the stability of photocurable primers through backbone modifications, such as using phosphorothioates, 2′‐O‐methyl or bridged nucleic acid modifications to prevent degradation by nucleases.

Furthermore, molecular dynamics simulations were employed to elucidate the mechanism by which the photocaged primer regulates DNA polymerase activity, focusing on its interaction within the ternary complex of primer, template, and DNA polymerase I (Klenow large fragment). The polymerase exhibits 3′−5′ exonuclease activity and 5′−3′ polymerase activity.^[^ ^27^ ^]^ The photocaged primer–template–polymerase complex exhibited higher root‐mean‐square deviation (RMSD) values and greater fluctuation amplitude compared to the mismatched primer–template–polymerase complex, indicating increased structural volatility (Figure S12, Supporting Information). Similarly, a larger radius of gyration was observed for the photocaged complex, suggesting a higher degree of spatial expansion and conformational flexibility (Figure S13, Supporting Information). Moreover, the polymerase displayed greater flexibility in the presence of the photocaged primer–template complex, further supporting the destabilizing effect of the photocaged primer (Figure S14, Supporting Information). Previous research demonstrated that the polymerase could exert its activity to extend the 3′‐end of the primer upon transitioning from an open to a closed conformation.^[^ ^28^ ^]^ We further compared the conformational changes of the polymerase bound to the mismatched primer‐template complex and the photocaged primer‐template complex over 100 ns kinetic simulations. The conformations of the polymerase are initially similar but begin to diverge during the middle and late stages of the simulation. The conformation of polymerase bound to the mismatched primer‐template complex tends to adopt a closed state, whereas the polymerase bound to the photocaged primer‐template complex favors an open conformation (Figure 1d). A local comparison graphic reveals that the polymerase helix undergoes a leftward shift due to the influence of 6‐NPOM nucleobases, resulting in the formation of a larger pocket (Figure 1e). The 6‐NPOM nucleobases primarily serve to fill the gap to stabilize the pocket structure. We conclude that the photocaged primer blocks the polymerase‐mediated primer extension reaction by inhibiting the conformational change of the polymerase.

### 2.2 Verification of Temporal Regulation of DNA Polymerase Activity

To evaluate whether the photoactivated technique can temporally regulate DNA polymerase activity, 365 nm UV was applied at specific time points to initiate primer extension. In the photoactivated PCR reaction, the quantity of amplification products increased exponentially upon 365 nm UV exposure, while no amplification products were observed without light activation (Figure 1f; Figure S15, Supporting Information). When 365 nm UV was applied after 5 Ct, exponential amplification was observed with corresponding delay of 5 Ct. In addition, we conducted the photoactivated droplet digital PCR (ddPCR) to investigate the temporal control of DNA polymerase activity. A photocaged reverse primer was designed and incorporated into a standard ddPCR reagent. After partitioning with Bio‐Rad droplet dPCR system, amplification was initiated by light. Following a standard PCR, high‐throughput readout of whole‐tube droplet was performed using Bio‐Rad droplet dPCR system. The results showed that fluorescence‐positive droplets were only detectable with light activation, and no fluorescence‐positive droplets were observed in the absence of light activation (Figure 1g,h). The results demonstrated that the proposed photocaged primer strategy can be used as an external stimulus to replace the hot‐start strategy, enabling temporal regulation of DNA polymerase activity and thereby initiating qPCR on demand. Furthermore, photoactivation strategy provides a rapid and temporal regulation approach, with the entire process taking only 50 s.

### 2.3 Light‐Start Recombinase Polymerase Amplification

Isothermal nucleic acid amplification technologies offer rapid and efficient amplification at a constant temperature, thereby eliminating the need for thermocycling as required in PCR.^[^ ^29^, ^30^ ^]^ Unlike PCR, which relies on heat to denature the double‐stranded template, primer invasion in isothermal nucleic acid amplification is driven by helicase, recombinase, or their combinations, enabling subsequent DNA synthesis by DNA polymerase.^[^ ^31^, ^32^, ^33^ ^]^ Isothermal amplification operates at moderate temperatures using mesophilic DNA polymerases, and the uncontrolled initiation is prone to generating nonspecific products. Although spatial isolation strategies can reduce nonspecific products in test tube reactions, premature nonspecific amplification is inevitable when isothermal nucleic acid amplification is performed in miniaturized compartments, such as droplets or microchambers.^[^ ^34^ ^]^ Prior to compartmentalization, all components of the reaction are mixed and the isothermal amplification reaction start spontaneously, resulting in increased nonspecific products. Therefore, suppressing enzymatic activity at ambient temperatures is critical for improving reaction specificity. Recombinase polymerase amplification (RPA) utilizes *Bsu* DNA polymerase, a large fragment of *Bacillus subtilis* DNA polymerase I with strand displacement activity, for DNA synthesis. In addition, RPA represents a particularly complex and crowded reaction system composed of recombinase, creatine kinase, recombinase‐loading factors, single‐stranded binding proteins, and DNA polymerase.^[^ ^35^ ^]^ In this context, we investigated whether the photocaged primer could regulate the activity of *Bsu* DNA polymerase and thereby enable recombinase polymerase amplification on demand.

**Figure 2:**
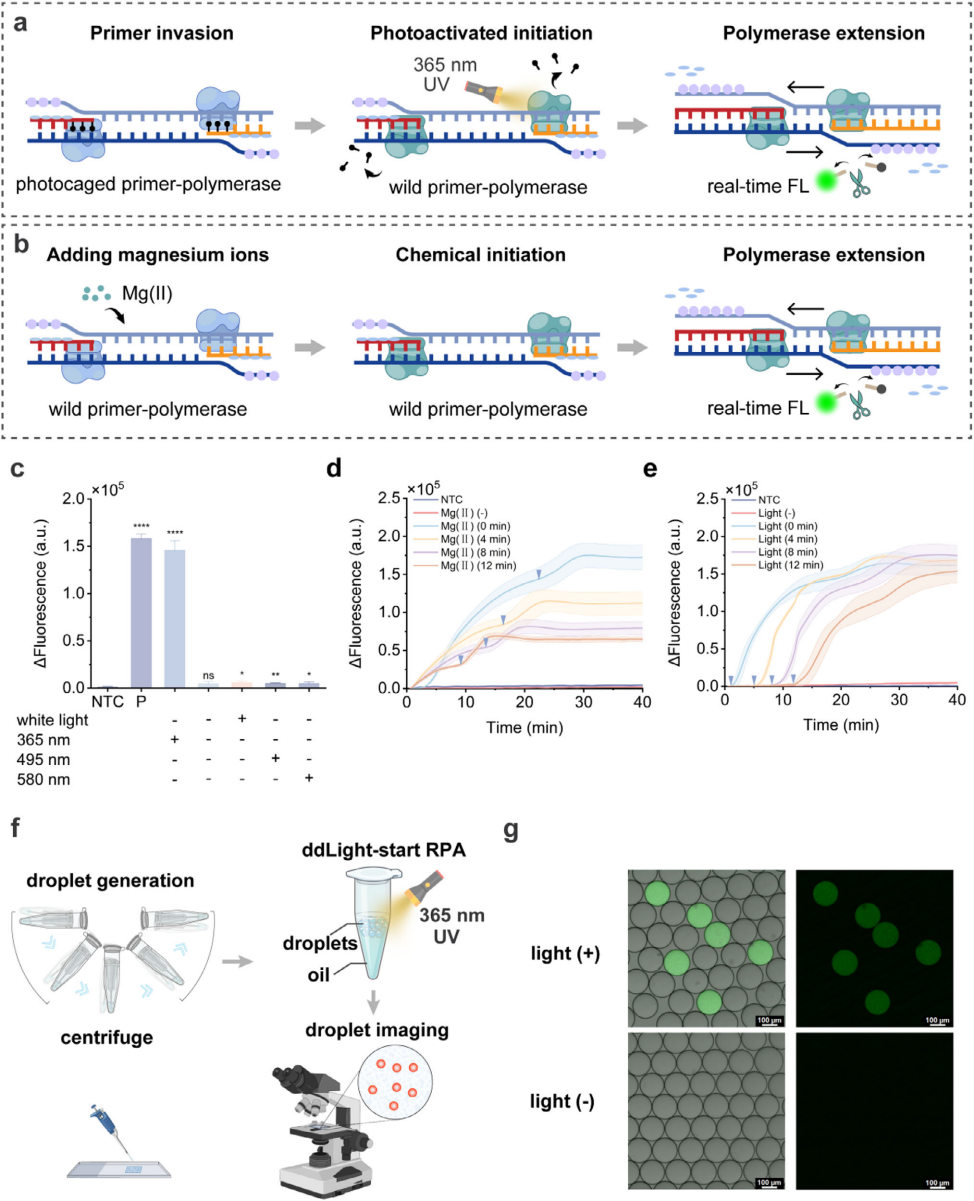
a) Schematic diagram of Light‐start RPA. Recombinase facilitates photocaged primer invasion into homologous sequences, after which DNA polymerase binds to the 3′‐end of the primer. Upon 365 nm UV exposure, the 6‐NPOM group at the 3′‐end is removed, enabling the polymerase to initiate primer extension. b) Schematic diagram of conventional RPA. Conventional RPA is initiated immediately upon the addition of magnesium ions. In this process, the recombinase facilitates the invasion of the primers into homologous sequences, after which DNA polymerase binds and initiates primer extension. c) Investigation of the feasibility of Light‐start RPA and the wavelength‐selectivity of photoactivation. The Light‐start RPA experiments were conducted using wild forward primers (CHIKV‐FP3) and photocaged reverse primers (CHIKV‐RP1‐NPOM). Fluorescence intensity was compared with NTC using a two‐tailed t‐test for significant differences: ****p < 0.0001; ***p < 0.001; **p < 0.01; *p < 0.05; ns represents no significant difference from NTC. d) Effect of premature nonspecific amplification on the amplification efficiency of conventional RPA. The reaction system preincubated with magnesium ions at 39 °C for varying durations, followed by real‐time fluorescence detection. Mg (II) (‐) indicates that the amplification reaction without magnesium ion. The inverted triangle in the figure indicates the time when amplification reaches the plateau stage. e) Study on the temporal regulation of Light‐start RPA by light. The reaction system was preincubated with magnesium ions for varying durations (0−12 min) before light was applied to initiate amplification. The inverted triangle in the figure indicates the time of light activation. f) The schematic diagram of the centrifugal‐driven droplet generation, the ddLight‐start RPA and the droplet counting. g) Feasibility of the ddLight‐start RPA for detection of linear ZIKV plasmid (1 fg µL^−1^) using photocaged reverse primer (ZIKV‐RP3‐NPOM). The fluorescent droplets indicate positive droplets, while the dark droplets indicate negative droplets. "Light+" indicates that amplification reagent was subjected to light treatment with 365 nm UV lamp (30 W) for 50 s. "Light‐" indicates that the reaction was not treated with a 365 nm UV lamp. P represents the positive control group, using a plasmid template (CHIKV plasmid) with wild forward and reverse primers (CHIKV‐FP3 and CHIKV‐RP1) for the conventional amplification system. NTC represents the blank control, using RNase‐free water instead of plasmid template. ΔFluorescence (a.u.) represents the difference between the fluorescence value at 40 min and the initial fluorescence value. Data are represented as mean ± standard error (*n* = 3 technical replicates).

We henceforth proposed the light‐start recombinase polymerase amplification (Light‐start RPA) (**Figure** **2**a). Under the optimized conditions (Figures S16–S20, Supporting Information), we validated the Light‐start RPA using a photocaged reverse primer. Conventional RPA was performed in parallel, except the initiation of the amplification is triggered by adding magnesium ions, with the reaction beginning once these ions enter the system^[^ ^35^ ^]^ (Figure 2b). Notably, Light‐start RPA could be specifically initiated by 365 nm UV, and exhibited amplification efficiency comparable to that of the conventional RPA (Figure 2c). The photocaged primers can be stably stored under ambient light for 24 h (Figure S21, Supporting Information). To evaluate whether the Light‐start RPA improves reaction specificity, we compared its performance with that of conventional RPA under conditions prone to nonspecific amplification. In conventional RPA, the time required for the amplification curve to reach the plateau stage is inversely proportional to the premixing time, and the longer the pre‐incubation time, the shorter the amplification curve to reach the platform stage (Figure 2d). However, increased pre‐incubation time leads to a reduction in the plateau fluorescence intensity, indicating that nonspecific amplification at ambient temperature diminishes the overall amplification efficiency. In contrast, Light‐start RPA, which relies on light rather than magnesium ions to initiate the reaction, enables precise temporal control of amplification (Figure 2e). This regulation effectively prevents premature nonspecific amplification prior to light activation, thereby improving reaction specificity. Although the fluorescence intensity at the plateau phase of the amplification curve slightly decreased when the pre‐incubation time exceeded 4 min, the photoactivatable DNA polymerase provided an additional level of control over isothermal nucleic acid amplification. Upon the introduction of magnesium ions, T4 uvsX recombinase binds to oligonucleotides and hydrolyzes ATP. The recombinase, in its ADP‐bound state, spontaneously degrades and is replaced by T4 gp32, thereby affecting amplification efficiency.^[^ ^35^ ^]^

To further verify the temporal control of Light‐start RPA, we integrated it with droplet digital technology and established the droplet digital Light‐start RPA (ddLight‐start RPA). We developed a centrifugal‐driven droplet generation approach to rapidly generate large quantities of monodisperse emulsion droplets (Figure 2f). The workflow of centrifugal‐driven droplet generator is illustrated in Figure S22 (Supporting Information). The droplet diameter is negatively correlated with centrifugal speed and decreases as the centrifugal speed increases (Figures S23–S28, Supporting Information). The rotational speed (3000 rpm) that produced the most uniform droplet diameters was selected by comparing the coefficients of variation of droplet diameters. To investigate the temporal control of ddLight‐start RPA can eliminate pre‐amplification during microreactor generation, we conducted the comparative test under light and no‐light activation. In the ddLight‐start RPA system, the 10 µL reaction mixture containing Light‐start RPA components and photocaged reverse primer was fully converted into droplets under a centrifugal force of 3000 rpm. After light activation, the amplification was carried out directly in the microcentrifuge tube, while the control group was not exposed to light. Droplet counting was conducted using planar imaging. Fluorescence‐positive droplets were observed in the light activation group, whereas no such droplets were detected in the non‐light activation group even after 20 min (Figure 2g). Poisson distribution parameters (λ) and the corresponding coefficient of variation (CV) were 0.14% and 1.14%, respectively. This indicates that the centrifugal‐driven droplet generation approach can be applied to the digital droplet assay. The estimated target concentration by ddLight‐start RPA is 221 copies µL^−1^. Together, these results suggest that temporal control of RPA can be achieved by light‐mediated regulation of DNA polymerase, enabling initiation of the amplification process on demand. This regulation allows the amplification reaction to be initiated after microreactor formation, thereby eliminating nonspecific amplification during droplet generation and enabling better integration into digital droplet workflows.

### 2.4 Application of Light‐Start Recombinase Polymerase to Pathogens Detection

**Figure 3:**
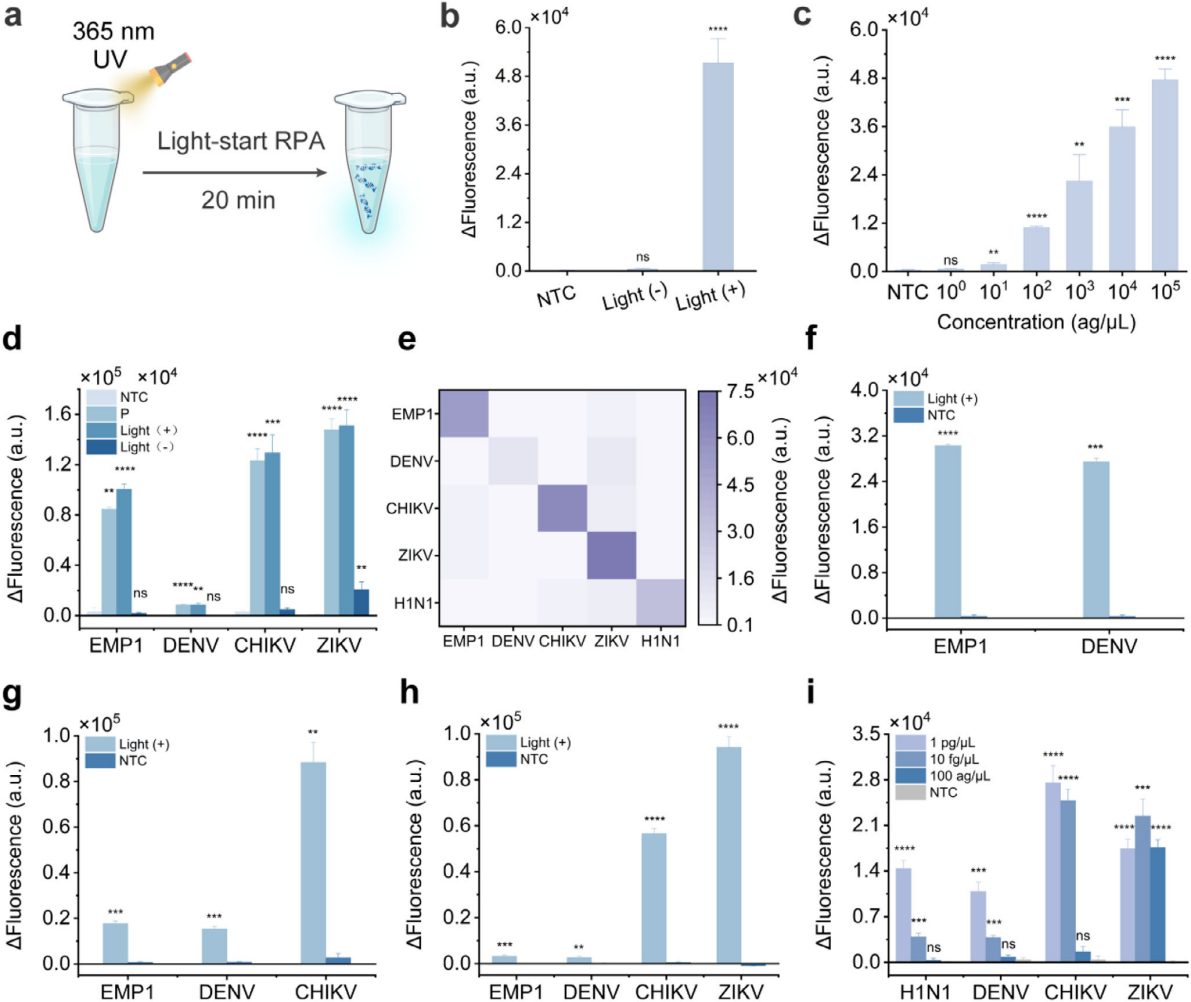
a) The operation flow diagram of a Light‐start RPA system. b) Feasibility study on regulating the activity of Bsu DNA polymerase using photocaged primer and thereby initiating RPA for detecting H1N1 plasmid (1 pg µL^−1^). c) Evaluation of the LoD of Light‐start RPA to detect H1N1 plasmid templates. d) Generalizability test of the photocaged primer design for detecting other pathogens (1 pg µL^−1^). e) Specificity analysis of Light‐start RPA. f–h) Evaluation of the Light‐start RPA for simultaneously detection of two, three, or four synthesized pathogens plasmids (1 pg µL^−1^). i) Investigation of the Light‐start RPA for simultaneously detection of four synthesized pathogens plasmids with concentrations range from 1 pg µL^−1^ to 100 ag µL^−1^. "Light+" indicates that amplification reagent was subjected to light treatment with 365 nm UV lamp (30 W) for 50 s. "Light‐" indicates that the reaction was not treated with a 365 nm UV lamp. P represents the positive control group, using a plasmid template with wild forward and reverse primers for the conventional amplification system. NTC represents the blank control, using RNase‐free water instead of plasmid template. ΔFluorescence (a.u.) represents the difference between the fluorescence value at 20 min and the initial fluorescence value. Fluorescence intensity was compared with NTC using a two‐tailed t‐test for significant differences: ****p < 0.0001; ***p < 0.001; **p < 0.01; *p < 0.05; ns represents no significant difference from NTC. Data are represented as mean ± standard error (*n* = 3 technical replicates).

As proof of the analytical application of the Light‐start RPA, we tested the applicability of the proposed Light‐start RPA for pathogens detection. We screened optimal primer pair using a plasmid template containing genome fragments of the influenza A virus (H1N1) (Figure S29, Supporting Information). Then, a photocaged reverse primer for regulation of DNA polymerase activity was designed, and Light‐start RPA assay was conducted (**Figure** **3**a,b). We established the limit of detection (LoD) of Light‐start RPA for H1N1 detection using gradient‐diluted H1N1 plasmids as the template (Figure 3c). The results showed that the Light‐start RPA could detect H1N1 plasmids down to 10 ag µL^−1^, comparable to that of the conventional RPA (Figure S30, Supporting Information).

To further investigate the universality of the photocaged primer design for regulating DNA polymerase activity, we extended the Light‐start RPA strategy to the detection of other pathogens. We constructed plasmids containing genome fragments of plasmodium falciparum (EMP1), flavivirus dengue virus (DENV), Zika virus (ZIKV), and Chikungunya virus (CHIKV), and designed photocaged primers for Light‐start RPA (Figure S31, Supporting Information). The Light‐start RPA can be extended to the detection of other pathogens (Figure 3d). And the cross‐over experiments demonstrated that the Light‐start RPA system exhibits excellent specificity (Figure 3e). In addition, the LoD of these Light‐start RPA assays for detecting EMP1, DENV, ZIKV and CHIKV plasmids was comparable to or exceeded that of conventional RPA (Figures S32–S35, Supporting Information).

The multiplexed nucleic acid amplification enables the simultaneous detection of multiple targets in a single reaction, improving detection efficiency and reducing processing time.^[^ ^36^, ^37^ ^]^ The flexibility of Light‐start RPA enables multiplexed detection by fine‐tuning the primer sequence. We first demonstrated the potential of the Light‐start RPA to detect two, three, or four synthesized pathogens plasmids simultaneously. The unoptimized panels can detect two or three targets simultaneously (Figure 3f,g), but not four targets (Figure 3h). By optimizing the ratios of the four primer probes, we explored the optimal signal‐to‐noise ratio for quadruplex detection. Using a series of gradient concentrations of the four synthesized pathogens plasmids, we demonstrated that the multiplexed Light‐start RPA could detect 10 fg µL^−1^ of synthesized pathogens plasmids from four pathogens (Figure 3i). Overall, the photoactivated strategy provides a competitive alternative to traditional chemical initiation in nucleic acid amplification. The Light‐start RPA offers flexibility, sensitivity, specificity and other advantages of isothermal nucleic acid amplification.

### 2.5 Practicability of Light‐Start RPA for Detection of H1N1 RNA in Clinical Samples

**Figure 4:**
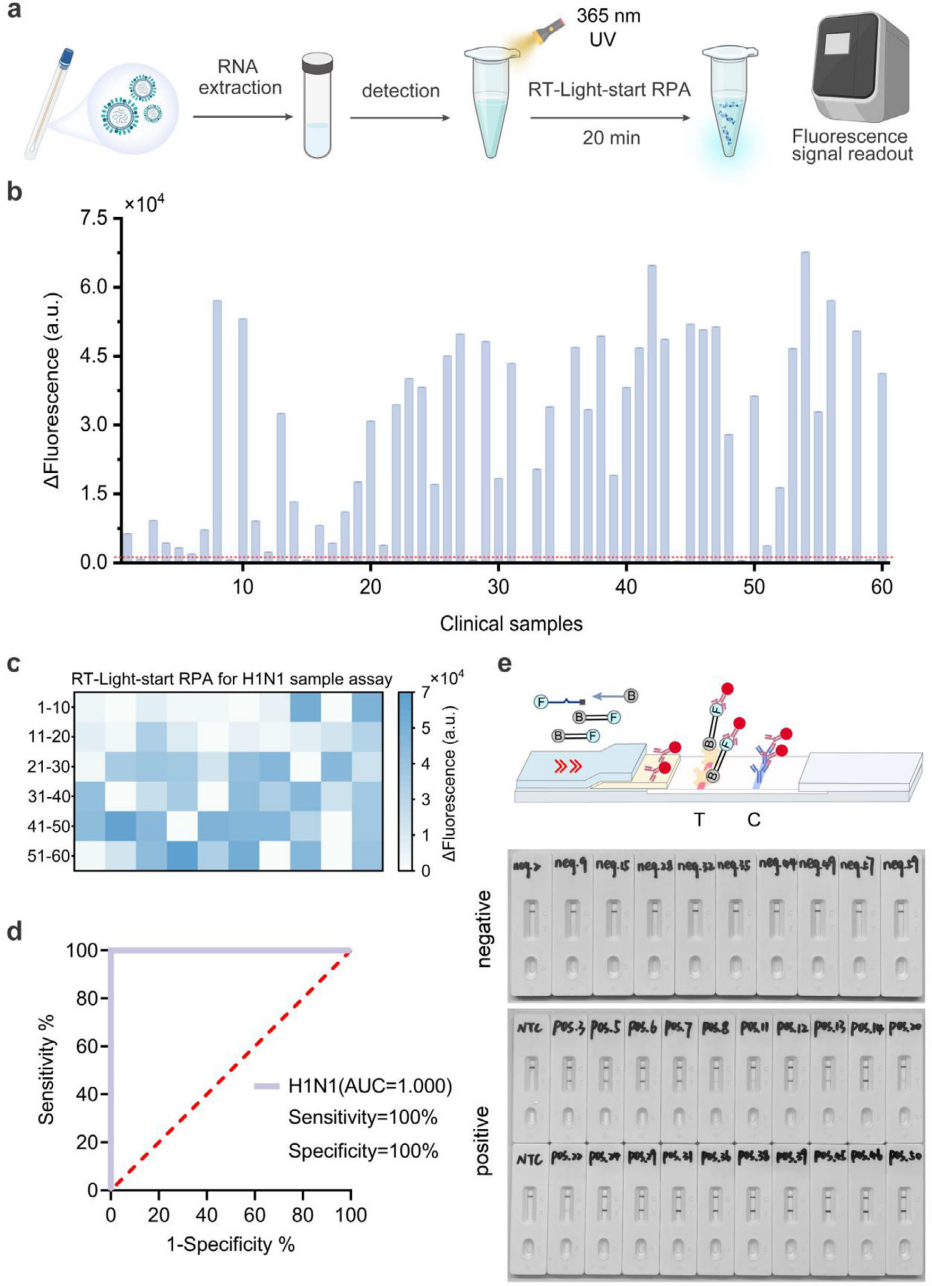
a) The schematic diagram of the RT‐Light‐start RPA for the detection of H1N1 RNA extracted from clinical samples. b) The detection results of 60 clinical samples using the RT‐Light‐start RPA. c) The heat map showing the detection results of 60 clinical H1N1 RNA samples using the RT‐Light‐start RPA. d) ROC curve analysis of the detection accuracy in clinical applications. e) Schematic diagram of RT‐Light‐start RPA‐LFA. f) Representative grayscale images of RT‐Light‐start RPA‐LFA detection results for clinical H1N1 RNA samples. The abbreviation “Neg.” denotes negative results, while “Pos.” indicates positive results. The numbers correspond to sample identifiers. ΔFluorescence (a.u.) represents the difference between the fluorescence value at 20 min and the initial fluorescence value.

To assess the clinical applicability of the Light‐start RPA, we performed Light‐start RPA assay with reverse transcriptase (RT‐Light‐start RPA) to detect H1N1 in 60 clinical samples from clinical settings (**Figure** **4**a). The fluorescence detection results distinguished between positive and negative samples based on a pre‐established threshold, calculated as the mean value of the NTC plus three times the standard deviation. This assay tested 60 clinical samples within 20 min, identifying 50 positive and 10 negative (Figure 4b,c). The results were also verified by a parallel CFDA‐approved RT‐qPCR method (Figure S36, Supporting Information). Cycle thresholds (Ct) were used to determine the negative and positive results of the samples. The area under the curve (AUC) of the subject operating characteristic (ROC) curve for H1N1 RNA in this study was 1.0, using the results provided by the hospital as a criterion. The results obtained using RT‐Light‐start RPA were consistent with those of RT‐qPCR, demonstrating 100% sensitivity and specificity (Figure 4d). In addition, we evaluated the adaptability of Light‐start RPA for instrument‐free point‐of‐care diagnostics by integrating it with a lateral flow assay (RT‐Light‐start RPA‐LFA). As illustrated in Figure 4e and Figure S37 (Supporting Information), a positive result is indicated when both the test and control lines show color, while a negative result is indicated when only the control line is colored. Representative clinical H1N1 RNA samples were tested using RT‐Light‐start RPA‐LFA, and the positive and negative results were consistent with those of RT‐Light‐start RPA and RT‐qPCR.

### 2.6 Construction of Integrated Light‐Start RPA Detection Device

**Figure 5:**
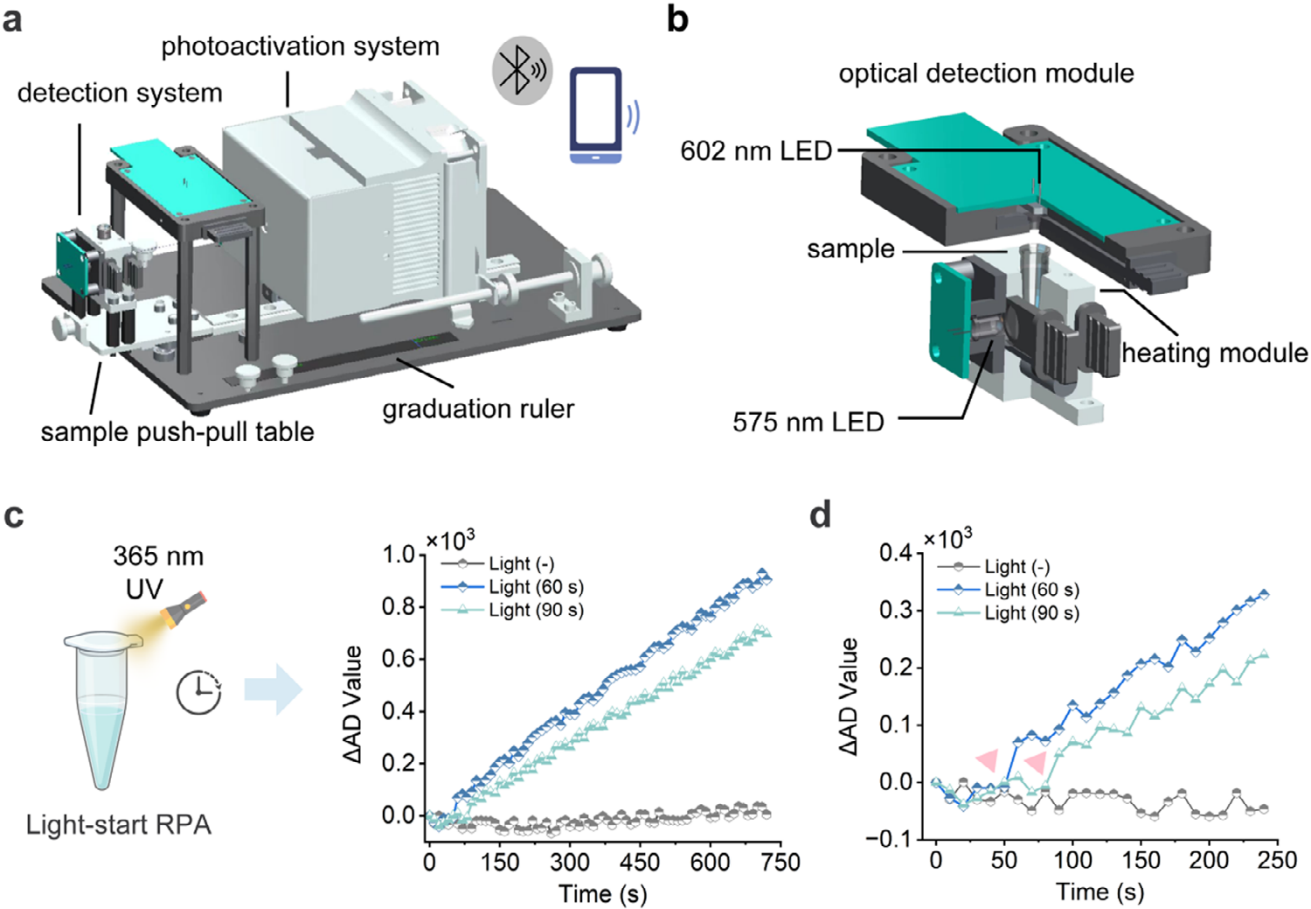
a) The modeling graph of the integrated Light‐start RPA detection device. The device consists of a photoactivation system and a detection system. b) The modeling graph of the optical detection module. c) The integrated Light‐start RPA detection device was used to investigate the initial regulation of Light‐start RPA by light. The reaction system was preincubated with magnesium ions for varying durations (60 or 90 s) before light was applied to initiate amplification. The real‐time fluorescence curve results were recorded over a 720 s period. d) The real‐time fluorescence curve results within 240 s. An inverted triangle is used to indicate the light activation time point.

Light‐start RPA strategy has the advantages of high sensitivity and speed, and is an excellent alternative for point‐of‐care nucleic acid testing. To demonstrate the adaptability of Light‐start RPA for resource‐limited settings, we designed an integrated device for both photoactivation and detection (**Figure** **5**a). This device consists of a photoactivation system and a detection system, where the detection system includes a sample push‐pull table, a heating module, and an optical detection module. In the optical detection module (Figure 5b), a 575 nm light‐emitting diode (LED) lamp serves as the fluorescence excitation source, while a 602 nm photodiode tube, positioned above the PCR tube, measures real‐time fluorescence signals. The heating module maintains the PCR tube at 39.5 °C. The core component in photoactivation system is a 365 nm LED lamp with a power output of 30 W (Figure S38, Supporting Information). This device is equipped with Bluetooth connectivity, enabling interfacing with both computer and mobile phone. Upon entering the test command, the device automatically turns the photoactivation system on and off, and subsequently performs real‐time fluorescence signal detection (Figure 5c,d). Subsequently, we investigated the feasibility of Light‐start RPA using the integrated detection device. The reaction system was preincubated with magnesium ions for varying durations, and then the photoactivation system was turned on to initiate amplification. The fluorescence signal is collected by the detection system every 10 s. The results indicate that the onset time of the amplification curve is associated with 365 nm illumination, and the device successfully integrates photoactivation with fluorescence detection.

## 3 Conclusion

In summary, we developed a simple and general photocaged primer approach to precisely‐control DNA polymerase activity. We systematically investigated the conditional blocking of photocaged primer to DNA polymerase activity. Molecular dynamic simulations shed light on that the photocaged primers cause the polymerase to remain in an open conformation, inhibiting the polymerase‐mediated primer extension reaction. We further demonstrated the applicability of photoactivatable DNA polymerases in recombinase polymerase amplification, and proposed the light‐start recombinase polymerase amplification (Light‐start RPA). The photoactivated strategy provided a competitive alternative to traditional chemical initiation in isothermal amplification. We demonstrated that the Light‐start RPA improved reaction specificity by preventing premature nonspecific amplification at ambient temperature before light activation. This approach provided superior temporal control to initiate amplification on demand, thereby integrating better into digital droplet workflows. Light‐start RPA enables the amplification reaction to be initiated after microreactor generation, thereby eliminating the nonspecific amplification during the generation process of the microreactor. With this advance, the digital droplet technology can be easily integrated with isothermal nucleic acid amplification to improve the reliability and sensitivity of nucleic acid detection. Compared to the spatial isolation strategies,^[^ ^38^, ^39^ ^]^ Light‐start RPA is a simple approach that can significantly expedite and simplify the analysis workflow. The future development of Light‐start digital isothermal amplification technology may facilitate further innovations in microfluidics. Although this study developed a centrifugal‐driven droplet generation method which effectively simplified the chip design and reduced the related costs, future development should further integrate the sample preparation and droplet generation steps to simplify the analysis process and shorten the turnaround time.

In addition, the Light‐start RPA offered universality, sensitivity, specificity and the capability to simultaneously detect four pathogens. In a cohort of nasopharyngeal samples, the real‐time RT‐Light‐start RPA and Light‐start RPA‐based lateral flow assay demonstrate 100% sensitivity and specificity in identifying influenza A virus, as validated by parallel RT‐qPCR analysis. We also designed an integrated device for both photoactivation and detection to streamline the analytical pipeline, and demonstrated Light‐start RPA is an attractive alternative for point‐of‐care nucleic acid testing in resource‐limited settings. Although there remain several future directions to pursue, it will be attractive to explore light‐start isothermal nucleic acid amplification with superior sensitivity and speed.

This study validates the photocaged primer strategy as an effective way for temporal control of DNA polymerase activity. However, the de‐caging of 6‐NPOM‐caged thymine relies on UV irradiation, which may pose a risk of nucleic acid damage. Although dose‑response experiments showed negligible impact on template integrity and amplification efficiency after UV exposure of 70 s, these results primarily reflect short‐term effects. Future efforts to develop light‐sensitive caging groups responsive to longer, less damaging wavelengths may improve biocompatibility and further expand the applicability of this strategy in molecular diagnostics. Furthermore, the photocaged primer can temporarily inhibit the polymerization activity of DNA polymerases, including *Taq* DNA polymerase and *Bsu* DNA polymerase, but they cannot completely resist the degradation by nucleases such as phi29 DNA polymerase. Further exploration will focus on enhancing the stability of the photocaged primer, for instance, by using thiophosphate, 2′‐O‐methyl or bridging nucleic acid modifications to prevent degradation by nucleases.

Overall, the photocaged primer approach expanded the conditional control toolbox of DNA polymerase activity, and offered unique features, including 1) simple, rapid and fine control of DNA polymerase activity, 2) precise temporal activation, 3) flexibility and generalizability of photocaged primer, and 4) broad applicability for polymerase‐mediated primer extension. More importantly, this work uncovers a new application for photoactivatable DNA polymerases in precisely initiating RPA. The temporal activation of isothermal amplification provides a versatile and promising framework for molecular diagnostics and microfluidics.

## 4 Experimental Section

### Photoactivated qPCR Assay

The qPCR assay was performed with the SYBR Green *Pro Taq* HS Premixed qPCR Kit (Accurate Biotech). The conventional qPCR assay was performed in a 10 µL reaction mixture prepared with a final concentration of 1× SYBR Green *Pro Taq* HS Premix, 0.2 µM of wild forward primer, 0.2 µM of wild reverse primer, a certain concentration of template, and DEPC‐treated water. As previously described, the photoactivated qPCR assay was performed in a 10 µL system, except that a photocaged reverse primer was used. The amplification was initiated by irradiating at 4 cm with a UV lamp (λ = 365 nm, 30 W) for 50 s.

qPCR was carried out with an initial denaturation at 95 °C for 30 s, followed by 35 cycles of denaturation at 95 °C for 5 s, and annealing/extension at 65 °C for 30 s. Fluorescence signals of the qPCR were recorded at 30 s intervals using the ABI Q3 (Thermo Fisher). Melting curves were obtained at the end of the qPCR procedure with program included denaturation 95 °C for 15 s, annealing at 60 °C for 60 s, and a melting analysis step from 60 to 95 °C with 0.15 °C increments.

### Photoactivated ddPCR Assay

For the photoactivated ddPCR assay, 11 µL of ddPCR supermix for probe, 2.97 µL of forward primer (10 µM), 2.97 µL of photocaged reverse primer (10 µM), 1.65 µL of probe (10 µM) and 1.21 µL of DEPC‐treated water were added to the ddPCR 96‐well plate to achieve a final volume of 19.8 µL premix. Subsequently, 2.2 µL of linear plasmid template (10 fg µL^−1^) was added to the ddPCR 96‐well plate to achieve a final volume of 22 µL amplification system. The ddPCR 96‐well plate was placed in an automated droplet generator to generate droplets, which were then irradiated with a UV lamp (λ = 365 nm, 30 W) from a distance of 4 cm for 50 s. Following a standard PCR, high‐throughput readout of whole‐tube droplet was performed using Bio‐Rad droplet dPCR system (QX600‐ddPCR). The conventional ddPCR assay was performed as described above, using a wild reverse primer and conducted without light activation.

### Light‐Start RPA Assay

The DNA Isothermal Rapid Amplification Kit (Fluorescent Type, Amplification Future Biotech) and the photocaged primers (Biosyntech) were used for the Light‐start RPA assay. Initially, 29.4 µL of A buffer was added to lyophilized enzyme pellet to prepare a suspended mixture. Subsequently, 2 µL of photocaged reverse primer (10 µM), 2 µL of wild forward primer (10 µM), 0.6 µL of probe (10 µM), and 11 µL of DEPC‐treated water were added to the suspended mixture to obtain a 45 µL premix. Finally, the Light‐start RPA assay was performed in a 10 µL of amplification system containing 0.5 µL of template, 9 µL of premix, and 0.5 µL of B buffer (containing magnesium ions). The system was irradiated with a UV lamp (λ = 365 nm, 30 W) at a distance of 4 cm for 50 s, followed by inversion (8–10 times) and centrifugation, and then incubated at 39 °C for 20 min. The fluorescence signals of the system were recorded at 30 s intervals using the ABI Q3. For RT‐Light‐start RPA assay, the RNA Isothermal Rapid Amplification Kit (Fluorescent Type, Amplification Future Biotech) was used.

To study the initiation regulation of Light‐start RPA by light irradiation, 10 µL amplification system was prepared as described previously. Specifically, the system without light irradiation was incubated at 39 °C after inversion and centrifugation, with fluorescence signals recorded at 30 s intervals using the ABI Q3. During the incubation and fluorescence reading, the system was irradiated with a 365 nm UV lamp (30 W, at a distance of 4 cm) at 0, 4, 8, or 12 min. After that, incubation continued at 39 °C, and fluorescence recording was performed for a total of 40 min.

### The ddLight‐Start RPA Assay

A centrifugal‐driven droplet generation approach to rapidly generate large quantities of monodisperse emulsion droplets was developed. The droplet generator was constructed by adding 10 µL of the sample into a polyethylene needle, which was then inserted into a 200 µL PCR tube containing 20 µL of fluorocarbon oil. Notably, the tip of the polyethylene needle should be submerged below the liquid level of the fluorocarbon oil. Subsequently, the generator was placed in a 1.5 mL centrifuge tube, which was then capped. The tube was placed in a bench centrifuge (Eppendorf) and subjected to centrifugation at a specific speed for 4 min. Finally, the droplets were transferred to notched slides and observed under a Leica upright fluorescence microscope (DM4 B) in bright field mode to record their morphology and size.

The ddLight‐start RPA assay was performed in a 10 µL of amplification system containing 0.5 µL of linear plasmid template, 9 µL of premix, and 0.5 µL of B buffer (containing magnesium ions). Then, the mixture was subjected to centrifugal‐driven droplet generation at 3000 rpm as previously described. The droplets were irradiated with a UV lamp (365 nm, 30 W) from a distance of 4 cm for 50 s, followed by incubation in a thermostatic metal bath at 39 °C for 20 min. Finally, the droplets were transferred onto a disposable cell counting plate (with a well depth of 200 µm), and their fluorescence were recorded using a Leica laser scanning confocal microscope (TCS‐SP8‐SR) in XY continuous scanning mode (10×). The droplets were statistically analyzed using the ImageJ software. The estimated target concentration (*c*) was calculated using Poisson statistics with the probability of positive droplets (*p*) and average volume of droplets (*V*).

(1) $$c=\frac{-\ln\left(1-p\right)}{V}$$

### Multiplexed Light‐Start RPA Assay

For the dual Light‐start RPA assay, 29.4 µL of A buffer was added to lyophilized enzyme pellet to prepare a suspended mixture. Subsequently, 2 µL of each primer (EMP1‐FP2, EMP1‐RP2‐NPOM, DENV‐FP, and DENV‐RP‐NPOM) at 10 µM, 0.6 µL of each probe (EMP1‐Probe, DENV‐Probe) at 10 µM, and 3.9 µL of DEPC‐treated water were added to the suspended mixture to achieve a final volume of 42.5 µL premix. Finally, the dual Light‐start RPA assay was performed in a 10 µL of amplification system containing 0.5 µL of 1 pg µL^−1^ each template (EMP1 plasmid, DENV plasmid), 8.5 µL of premix, and 0.5 µL of B buffer (containing magnesium ions).

For the triple Light‐start RPA assay, 29.4 µL of A buffer was added to lyophilized enzyme pellet to prepare a suspended mixture. Subsequently, 1.5 µL of each primer (EMP1‐FP2, EMP1‐RP2‐NPOM, DENV‐FP, DENV‐RP‐NPOM, CHIKV‐FP3, and CHIKV‐RP1‐NPOM) at 10 µM, 0.45 µL of each probe (EMP1‐Probe, DENV‐Probe, and CHIKV‐Probe) at 10 µM, and 0.25 µL of DEPC‐treated water were added to the suspended mixture to achieve a final volume of 40 µL premix. Finally, the dual Light‐start RPA assay was performed in a 10 µL of amplification system containing 0.5 µL of 1 pg µL^−1^ each template (EMP1 plasmid, DENV plasmid, and CHIKV plasmid), 8 µL of premix, and 0.5 µL of B buffer (containing magnesium ions).

For the quadruple Light‐start RPA assay, 29.4 µL of A buffer was added to lyophilized enzyme pellet to prepare a suspended mixture. Subsequently, 1 µL of each primer (H1N1‐FP1, H1N1‐RP4‐NPOM, DENV‐FP, and DENV‐RP‐NPOM) at 10 µM, 0.5 µL of each primer (CHIKV‐FP3, CHIKV‐RP1‐NPOM, ZIKV‐FP3, and ZIKV‐RP3‐NPOM) at 10 µM, 0.3 µL of each probe (H1N1‐Probe, DENV‐Probe) at 10 µM, 0.15 µL of each probe (ZIKV‐Probe, CHIKV‐Probe) at 10 µM, and 0.74 µL of DEPC‐treated water were added to the suspended mixture to achieve a final volume of 37.5 µL premix. Finally, the dual Light‐start RPA assay was performed in a 10 µL of amplification system containing 0.5 µL of 1 pg µL^−1^ each template (H1N1 plasmid, EMP1 plasmid, DENV plasmid, and CHIKV plasmid), 7.5 µL of premix, and 0.5 µL of B buffer (containing magnesium ions). The amplification system was irradiated with a 365 nm UV lamp (30 W) at a distance of 4 cm for 50 s, followed by inversion (8–10 times) and centrifugation, and then incubated at 39 °C for 20 min. The fluorescence signals of the system were recorded at 30 s intervals using the ABI Q3.

### Conventional RPA Assay

The conventional RPA assay was performed as Light‐start RPA assay, except using wild forward primer and reverse primer. The DNA Isothermal Rapid Amplification Kit (Fluorescent Type, Amplification Future Biotech) was used for the conventional amplification assay. One lyophilized enzyme pellet was suspended in 29.4 µL of A buffer to prepare a suspended mixture. Subsequently, 2 µL of wild forward primer (10 µM), 2 µL of wild reverse primer (10 µM), 0.6 µL of probe (10 µM), and 11 µL of DEPC‐treated water were added to prepare the premix. Then, 9 µL of premix was mixed with 0.5 µL of template. For spatial separation of chemical initiator (magnesium ions), 0.5 µL of B buffer was added to the tube cap. Finally, the conventional RPA assay was performed in a 10 µL amplification system. The system was thoroughly mixed by inversion (8–10 times) followed by centrifugation. Subsequently, the system was incubated at 39 °C for 20 min, with fluorescence signals recorded at 30 s intervals using the ABI Q3.

### Statistical Analysis

Two‐tailed t‐test (and nonparametric tests) were used to determine the statistical differences among samples. Statistical analyses were performed using GraphPad Prism 9.5.0. Data were represented as mean ± standard error. Differences were considered significant at the following P values: *p < 0.05, **p < 0.01, ***p < 0.001, and ****p < 0.0001. Sample size (*n*) for each statistical analysis was given in each caption of figures.

### Ethical Approval

The clinical samples tested in this study were approved by the Ethics Committee of Chongqing Medical University (reference number: 2 024 128). Clinical samples for this research were collected from patients who visited the First Affiliated Hospital of Chongqing Medical University for routine diagnosis and treatment. All participants provided informed consent prior to sample collection. The clinical samples were fully de‐identified prior to analysis.

## Conflict of Interest

The authors declare no conflict of interest.

## Supporting information



Supporting Information

## Data Availability

The data that support the findings of this study are available from the corresponding author upon reasonable request.
