# Supplementary material for: Universal Regulation of DNA Polymerase via Photocaged Primer Enables Light‐Start Isothermal Amplification on Demand
Source: Adv Sci (Weinh). 2025 Oct 24;13(3):e11245. doi: 10.1002/advs.202511245 (PMC12806212; doi:10.1002/advs.202511245)
Supplement: Supplementary file 1 — Supporting Information [file ADVS-13-e11245-s001.docx]

*Supplementary Information*

Universal Regulation of DNA Polymerase via Photocaged Primer Enables Light-start Isothermal Amplification on Demand

*Min Qing,* ^[a],†^ *Yufan Qin,* ^[a],†^ *Zhijin Li,* ^[a]^ *Xiufang Yu,* ^[b]^ *Jianbo Wang,* ^[a]^ *Xiaoqiong Liu,* ^[a]^ *Xin Chen,* ^[a]^ *Chao Yu* ^[a],*^

^[a]^Min Qing, Yufan Qin, Zhijin Li, Jianbo Wang, Xiaoqiong Liu, Xin Chen, Chao Yu

Chongqing Key Laboratory for Pharmaceutical Metabolism Research, College of Pharmacy

Chongqing Medical University

Chongqing 400016, P. R. China

E-mail: yuchao@cqmu.edu.cn

^[b]^Xiufang Yu

Department of Infectious Diseases

the First Affiliated Hospital of Chongqing Medical University

Chongqing 400016, P. R. China

^†^ These authors contributed equally to this work.

**Table of contents**

Experimental Section 4

Table S1. Nucleic acid sequences. 8

Table S2. Plasmids information. 11

Table S3. The information of Influenza A clinical samples. 13

Figure S1 14

Figure S2 15

Figure S3 16

Figure S4 17

Figure S5 18

Figure S6 19

Figure S7 20

Figure S8 21

Figure S9 22

Figure S10 23

Figure S11 24

Figure S12 25

Figure S13 26

Figure S14 27

Figure S15 28

Figure S16 29

Figure S17 31

Figure S18 32

Figure S19 33

Figure S20 34

Figure S21 35

Figure S22 36

Figure S23 37

Figure S24 38

Figure S25 38

Figure S26 39

Figure S27 39

Figure S28 40

Figure S29 41

Figure S30 42

Figure S31 43

Figure S32 44

Figure S33 45

Figure S34 46

Figure S35 47

Figure S36 48

Figure S37 49

Figure S38 50

**Experimental Section**

**Culture and extraction of DNA plasmids.**

For transformation and culture of template plasmids were performed with the Top10 competent cells. Cultured plasmids were extracted using the TIANpure Midi Plasmid Kit (TIANGEN Biotech), with all steps performed in accordance with the instructions provided. Subsequently, the nucleic acid concentrations were quantified using a N60 NanoDrop spectrophotometer (Implen). The extracted plasmids were diluted in a gradient using DEPC-treated water (Beyotime Biotech).

**Preparation of linearized plasmids.**

The pathogens genes were synthesized and cloned into the pUC57 or pBluescript II SK (+) vectors (Sangon Biotech), with plasmid information provided in Supplementary Table S2. The constructed plasmid contained a T7 promoter and two enzymatic sites, Hind III and BamH I. The linearized plasmid was obtained by restriction endonuclease. A 20 μL system was prepared by mixing 10 μL of plasmids (100 ng/μL), 2 μL of 10× CutEZTM Buffer, 7 μL of DEPC-treated water, and 1 μL of BeyoFast^TM^ BamH Ⅰ (D5625, Beyotime Biotech), followed by incubation at 37 °C for 15 min. The linearized plasmids were analyzed and purified using 1.5% agarose gel electrophoresis, and the purified linear DNA was extracted using the EZ-10 Column DNA PAGE Gum Recovery Kit (Sangon Biotech). The DNA concentration was quantified using a N60 NanoDrop spectrophotometer (Implen) and stored at -20 °C. For subsequent ddLight-start RPA and photoactivated ddPCR assay, the linear plasmid concentration was diluted using DEPC-treated water.

**Molecular dynamics simulation.**

The three-dimensional structure of the protein was obtained from the Protein Data Bank (PDB) with the ID 8OO6. The mismatched primer and template DNA were constructed using Maestro 13.0 software, while the photocaged primer was designed with the same software in conjunction with the NPOM structure, followed by manual modifications.

After obtaining the target structures, the original DNA from the 8006 crystal structure was used as a reference, with the mismatched primer and template incorporated into it. The structures were prepared using the Protein Preparation Wizard in Maestro (Schrödinger, LLC, NY, USA) and then globally optimized with the Prime module. This process aimed to achieve the mismatched primer-template-protein and photocaged primer-template-protein ternary complexes.

In this study, molecular dynamics simulations of small molecule compound-protein complexes were performed using the Desmond program (Schrödinger, LLC, New York, USA). Before initiating the simulation, each complex system was dissolved in a SPC solvent box with a periodic boundary size of 10 × 10 × 10 Å, creating the complex-solvent system. A specific quantity of Na^+^ or Cl^-^ was then added to achieve electrical neutrality. The OPLS4 force field was selected for the simulations. Once the system was conFigured, the Molecular Dynamics module was utilized to conduct the formal simulations. The temperature was set to 300 K, and the pressure was maintained at 1.01325 bar. An NPT simulation was performed for 100 ns, with energy recorded every 1.0 ps and orbital coordinates monitored every 20 ps. A total of 5000 frames of conformations were output during the simulations.

The behavior of the mismatched primer-template-protein and photocaged primer-template-protein ternary complexes was analyzed using the trajectory analysis function in Maestro software. The 3D conformations were extracted and visualized using PyMOL 2.5.5. Additionally, the root-mean-square deviation (RMSD), radius of gyration (RoG), and root-mean-square fluctuation (RMSF) were calculated for the selected complexes to assess their structural stability, convergence, and flexibility.

**Gel electrophoresis assay.**

To verify the conversion of photocaged primers into wild primers, the products of light treatment were analyzed using polyacrylamide gel electrophoresis (PAGE). A panel was designed in which 10 μL of 0.5 μM photocaged primers were separately treated with a UV lamp (λ = 365 nm, 30 W) for 0 s, 15 s, 30 s, 45 s, 60 s, 120 s, and 180 s at a distance of 4 cm. Subsequently, the samples were analyzed using the 12% PAGE and imaged with GenoSens 2150 imaging system (Clinx Scientific Instrument). To investigate the effect of photocaged primer on preventing non-specific primer dimer formation, PCR assay was performed in a 10 μL system, prepared with a final concentration of 1× SYBR Green *Pro Taq* HS Premix (pre-thermal activation), 0.4 μM of EMP1-FP2-dimer, 0.4 μM of reverse primer (EMP1-RP2-NPOM or EMP1-RP2), and 1 pg/μL EMP1 plasmid. The the products were analyzed using the 2% agarose gel and imaged with Gel Doc XR+ imaging system (Bio-Rad Laboratories). To study exonuclease resistance of photocaged primer against phi29 DNA polymerase, the primer degradation assay was performed in a 20 μL of system containing 4 U of phi29 DNA polymerase and 1 μM primer. The reaction was incubated at 30 °C and then terminated by heat inactivation at 65 °C. The the products were analyzed using the 12% PAGE and imaged with Gel Doc XR+ imaging system (Bio-Rad Laboratories). The intensity of the bands was analyzed using the Image J software.

**H1N1 RNA clinical samples collection.**

A total of stored 60 human nasopharyngeal samples were provided by The First Affiliated Hospital of Chongqing Medical University (Supplementary Table S3). The Influenza A and B virus nucleic acid detection Kit (ZJ Bio-Tech, No. ME-0001) was used to extract H1N1 RNA from clinical samples in biosafety level II laboratory, and the extracted H1N1 RNA samples were tested using the standard qRT-PCR.

For the RT-Light-start RPA assay of clinical samples, the testing procedure was performed as described previously, with the exception that amplification was carried out in a 10 μL system containing 0.5 μL of H1N1 RNA samples, 9 μL of premix, and 0.5 μL of B buffer (containing magnesium ions).

For the RT-Light-start RPA-LFA assay of clinical samples, the RNA Isothermal Rapid Amplification Kit (Colloidal gold test strip Type, Amplification Future Biotech) was used. Initially, 29.4 μL of A buffer was added to lyophilized enzyme pellet to prepare a suspended mixture. Subsequently, 2 μL of biotin labeled forward primer (H1N1-Biotin-FP1, 10 μM), 2 μL of photocaged reverse primer (H1H1-RP4-NPOM, 10 μM), 0.6 μL of LFA-probe (H1N1-LFA-Probe, 10 μM), and 11 μL of DEPC-treated water were added to the suspended mixture to obtain a 45 μL premix. Finally, the RT-Light-start RPA-LFA assay was performed in a 10 μL of amplification system containing 0.5 μL of H1N1 RNA samples, 9 μL of premix, and 0.5 μL of B buffer (containing magnesium ions). The system was irradiated with a 365 nm UV lamp (30 W) at a distance of 4 cm for 50 s, followed by inversion (8-10 times) and centrifugation, and then incubated at 39 °C for 15 min. Add 5 μL of amplicons to 95 μL of DEPC-treated water. Then, transfer 80 μL of diluted amplicons to the sample well of the colloidal gold test strip. The results are read after 10 min, and images are captured using a phone.

**Table S1**. Nucleic acid sequences.

| **Name** | **Sequence (5'-3')** |
| --- | --- |
| EMP1-FP1 | GAGGAAGATAAAGATGCCAAATATATGTTT |
| EMP1-FP2 | TGAGGAAGATAAAGATGCCAAATATATGTT |
| EMP1-FP3 | TGGTGAGGAAGATAAAGATGCCAAATATAT |
| EMP1-FP4 | TGTGGTGAGGAAGATAAAGATGCCAAATAT |
| EMP1-FP2-dimer | CAAATATATGTTTGATAGGATAGGGAAAGA |
| EMP1-RP1 | TTTTTCTTTTATAAGCTCGCACGGATCTGT |
| EMP1-RP2 | CGTTTTTCTTTTATAAGCTCGCACGGATCT |
| EMP1-RP2-M1 | CGTTTTTCTTTTATAAGCTCGCACGGATCA |
| EMP1-RP2-M2 | CGTTTTTCTTTTATAAGCTCGCACGGACCC |
| EMP1-RP2-M3 | CGTTTTTCTTTTATAAGCTCGCACGGAACA |
| EMP1-RP2-M4 | CGTTTTTCTTTTATAAGCTCGCACGGAGCG |
| EMP1-RP2-NPOM | CGTTTTTCTTTTATAAGCTCGCACGGA/NPOMdT/C/NPOMdT/ |
| EMP1-RP2-1NPOM | CGTTTTTCTTTTATAAGCTCGCACGGATC/NPOMdT/ |
| EMP1-RP2-5'NPOM | CG/NPOMdT/T/NPOMdT/TTCTTTTATAAGCTCGCACGGATCT |
| EMP1-RP2-mNPOM | CGTTTTTCTTTTA/NPOMdT/AAGC/NPOMdT/CGCACGGATCT |
| EMP1-Probe | AGTGAAAAATGCTGCAAATGTCTTTAAAGA/iHEXdT/T/idSp/T/iBHQ1dT/TGAAAGGAAATTTGA/3C3 Spacer/ |
| DENV-FP | ATTCCTAAGATTTCTAGCCATACCTCCAACAGC |
| DENV-RP | GTCACAGATCTTTTTCTCCTGTTCATTATGTTC |
| DENV-RP-M | GTCACAGATCTTTTTCTCCTGTTCATTATGAAC |
| DENV-RP-NPOM | GTCACAGATCTTTTTCTCCTGTTCATTATG/NPOMdT//NPOMdT/C |
| DENV-Probe | TCAAGAAGAATGGAGCGATTAAAGTGTTACGGGG/iTAMdT//idSp//iBHQ2dT/CAAAAAAGAAATCTC/3C3 Spacer/ |
| CHIKV-FP1 | TGACGCACAAGAAGGAGGTCGTGCTAACCG |
| CHIKV-FP2 | CGCACAAGAAGGAGGTCGTGCTAACCGTGC |
| CHIKV-FP3 | CACAAGAAGGAGGTCGTGCTAACCGTGCCG |
| CHIKV-RP1 | ATAGTAGGGTACAGCTCATAATAGTACAAGATT |
| CHIKV-RP2 | TAGTAGGGTACAGCTCATAATAGTACAAGATT |
| CHIKV-RP3 | AGTAGGGTACAGCTCATAATAGTACAAGATT |
| CHIKV-RP1-M | ATAGTAGGGTACAGCTCATAATAGTACAAGAAA |
| CHIKV-RP1-NPOM | ATAGTAGGGTACAGCTCATAATAGTACAAGA/NPOMdT//NPOMdT/ |
| CHIKV-Probe | AGGTTACGTGGGGCAACAACGAGCCGTA/iROXdT/AA/idSp//iBHQ2dT/ATTGGCCGCAGTTAT/3C3 Spacer/ |
| CHIKV-Probe (forddPCR) | /5ROX/TCTGCAAACGGTACAGCCCACGGCCA/3BHQ2/ |
| ZIKV-FP1 | GATCTGTATTACCTGACCATGAACAATAAGCA |
| ZIKV-FP2 | AAAGAGTGGTTTCATGACATCCCATTGCCT |
| ZIKV-FP3 | AAGAGTGGTTTCATGACATCCCATTGCCTT |
| ZIKV-RP1 | ATCCTTGAATTCTACCAATGCCTCTTTGTT |
| ZIKV-RP2 | CATCCTTGAATTCTACCAATGCCTCTTTGT |
| ZIKV-RP3 | GGCTCCCCAGAACGACGACGGTTTGCCTCTTG |
| ZIKV-RP4 | GCATCCTTGAATTCTACCAATGCCTCTTTG |
| ZIKV-RP3-M | GGCTCCCCAGAACGACGACGGTTTGCCTCAAG |
| ZIKV-RP3-NPOM | GGCTCCCCAGAACGACGACGGTTTGCCTC/NPOMdT//NPOMdT/G |
| ZIKV-Probe | AGAGTGGTTTCATGACATCCCATTGCCT/i6FAMdT/GG/idSp/A/iBHQ1dT/GCTGGGGCAGACAC/3C3 Spacer/ |
| H1N1-FP1 | ATGAGTCTTCTAACCGAGGTCGAAACGTAC |
| H1N1-FP2 | CTCAAAGCCGAGATCGCACAGAGACTTGAAG |
| H1N1-RP1 | AGCGTGAACACAAATCCTAAAATCCCCTTA |
| H1N1-RP2 | CCTTAGTCAGAGGTGACAGGATTGGTCTTG |
| H1N1-RP3 | TTAGTCAGAGGTGACAGGATTGGTCTTGTCT |
| H1N1-RP4 | GTCAGAGGTGACAGGATTGGTCTTGTCTTT |
| H1N1-RP4-M | GTCAGAGGTGACAGGATTGGTCTTGTCAAT |
| H1H1-RP4-NPOM | GTCAGAGGTGACAGGATTGGTCTTGTC/NPOMdT//NPOMdT/T |
| H1N1-Probe | TGAAGATGTCTTTGCAGGGAAGAACACCGA/iHEXdT//idSp//iBHQ1dT/TGAGGTTCTCATGGAA/3C3 Spacer/ |
| H1N1-Biotin-FP1 | /5Biotin/ATGAGTCTTCTAACCGAGGTCGAAACGTAC |
| H1N1-LFA-Probe | /56FAM/TTCCATGAGAACCTCAAGATCGGTGTTCTTC/idSp/CTGCAAAGACATCTTCA/3C3 Spacer/ |

**Abbreviations and modifications:**

/NPOMdT/: 6-nitropiperonyloxymethyl that can be modified at T in the sequence.

/iHEXdT/：Hexachloro fluorescein internal modification.

/iBHQ1dT/: Black Hole Quencher-1 internal modification, which is used to quench yellow-green and yellow dyes, such as FAM and HEX.

/idSp/：dSpacer (1,2'-Dideoxyribose) internal modification.

/3C3 Spacer/：3’ C3 Spacer modification.

/iTAMdT/：Carboxy tetramethyl rhodamine internal modification.

/iBHQ2dT/: Black Hole Quencher-2 internal modification, which is used to quench yellow-orange dyes, such as TAMRA and ROX.

/iROXdT/：Carboxy-X-Rhodamine internal modification.

/i6FAMdT/：6-carboxy-fluorescein internal modification.

/5ROX/：5’ Carboxy-X-Rhodamine modification.

/3BHQ2/：3’ Black Hole Quencher-2 modification.

/5Biotin/: 5’ Biotin modification.

/56FAM/: 5’ 6-carboxy-fluorescein modification.

/5HEX/：5’ Hexachloro fluorescein modification.

/3BHQ1/：3’ Black Hole Quencher-1 modification

**Table S2**. Plasmids information.

| **Name** | **Sequence (5'-3')** |
| --- | --- |
| EMP1 gene·GenBank:>NC_037281.1:28490-28743 (Note: Cloning vector: pUC57) | ATGGCGGCGCAAAGTAGTGGTGGGGGTGGAGGTTGTGGTGAGGAAGATAAAGATGCCAAATATATGTTTGATAGGATAGGGAAAGAAGTGCACGACGAAGTGAAAAATGCTGCAAATGTCTTTAAAGATTATTTGAAAGGAAATTTGACAATATCAACAATTTTTGGTGAGGAAACAGTTGCCTTCACAGATCCGTGCGAGCTTATAAAAGAAAAACGTGATGAACTTCTTGCTGCTCGCGGTGATCCGTGCGG |
| DENV gene·GenBank:>NC_001477.1:61-420 (Note: Cloning vector: pBluescript II SK (+), digestive site: HindIII/BamHI) | AAGCTTTTCTAACAGTTTTTTATTAGAGAGCAGATCTCTGATGAACAACCAACGGAAAAAGACGGGTCGACCGTCTTTCAATATGCTGAAACGCGCGAGAAACCGCGTGTCAACTGTTTCACAGTTGGCGAAGAGATTCTCAAAAGGATTGCTTTCAGGTCAAGGACCCATGAAACTGGTGATGGCTTTTATAGCATTCCTAAGATTTCTAGCCATACCTCCAACAGCAGGAATTTTGGCTAGATGGGGCTCATTCAAGAAGAATGGAGCGATTAAAGTGTTACGGGGTTTCAAAAAAGAAATCTCAAACATGTTGAACATAATGAACAGGAGAAAAAGATCTGTGACCATGCTCCTCATGCTGCTGGATCC |
| CHIKV gene·GenBank:>NC_004162.2:9453-9707 (Note: Cloning vector: pBluescript II SK (+), digestive site: HindIII/BamHI) | AAGCTTAAACTATCAAGAAGAGTGGGTGACGCACAAGAAGGAGGTCGTGCTAACCGTGCCGACTGAAGGGCTCGAGGTTACGTGGGGCAACAACGAGCCGTATAAGTATTGGCCGCAGTTATCTGCAAACGGTACAGCCCACGGCCACCCGCATGAGATAATCTTGTACTATTATGAGCTGTACCCTACTATGACTGTAGTAGTTGTGTCAGTGGCCTCGTTCATACTCCTGTCGATGGTGGGTATGGCAGTGGGGATGTGGGATCC |
| ZIKV gene·GenBank:>NC_012532.1:1546-1798 (Note: Cloning vector: pBluescript II SK (+), digestive site: HindIII/BamHI) | AAGCTTAGGCCTTGACTTTTCAGATCTGTATTACCTGACCATGAACAATAAGCATTGGTTGGTGCACAAAGAGTGGTTTCATGACATCCCATTGCCTTGGCATGCTGGGGCAGACACCGGAACTCCACACTGGAACAACAAAGAGGCATTGGTAGAATTCAAGGATGCCCACGCCAAGAGGCAAACCGTCGTCGTTCTGGGGAGCCAGGAAGGAGCCGTTCACACGGCTCTCGCTGGAGCTCTAGAGGCTGAGATGGATGGATCC |
| H1N1 gene·GenBank:>NC_002016.1:26-276 (Note: Cloning vector: pBluescript II SK (+), digestive site: HindIII/BamHI) | AAGCTTATGAGTCTTCTAACCGAGGTCGAAACGTACGTTCTCTCTATCATCCCGTCAGGCCCCCTCAAAGCCGAGATCGCACAGAGACTTGAAGATGTCTTTGCAGGGAAGAACACCGATCTTGAGGTTCTCATGGAATGGCTAAAGACAAGACCAATCCTGTCACCTCTGACTAAGGGGATTTTAGGATTTGTGTTCACGCTCACCGTGCCCAGTGAGCGAGGACTGCAGCGTAGACGCTTTGTCCAAAATGCCCTGGATCC |

Note: The underlined sequence represents the cleavage site of the restriction endonuclease enzyme.

**Table S3.** The information of Influenza A clinical samples.

| **Pos.1** | **Neg.2** | **Pos.3** | **Pos.4** | **Pos.5** | **Pos.6** | **Pos.7** | **Pos.8** | **Neg.9** | **Pos.10** |
| --- | --- | --- | --- | --- | --- | --- | --- | --- | --- |
| **+** | **-** | **+** | **+** | **+** | **+** | **+** | **+** | **-** | **+** |
| **Pos.11** | **Pos.12** | **Pos.13** | **Pos.14** | **Neg.15** | **Pos.16** | **Pos.17** | **Pos.18** | **Pos.19** | **Pos.20** |
| **+** | **+** | **+** | **+** | **-** | **+** | **+** | **+** | **+** | **+** |
| **Pos.21** | **Pos.22** | **Pos.23** | **Pos.24** | **Pos.25** | **Pos.26** | **Pos.27** | **Neg.28** | **Pos.29** | **Pos.30** |
| **+** | **+** | **+** | **+** | **+** | **+** | **+** | **-** | **+** | **+** |
| **Pos.31** | **Neg.32** | **Pos.33** | **Pos.34** | **Neg.35** | **Pos.36** | **Pos.37** | **Pos.38** | **Pos.39** | **Pos.40** |
| **+** | **-** | **+** | **+** | **-** | **+** | **+** | **+** | **+** | **+** |
| **Pos.41** | **Pos.42** | **Pos.43** | **Neg.44** | **Pos.45** | **Pos.46** | **Pos.47** | **Pos.48** | **Neg.49** | **Pos.50** |
| **+** | **+** | **+** | **-** | **+** | **+** | **+** | **+** | **-** | **+** |
| **Pos.51** | **Pos.52** | **Pos.53** | **Pos.54** | **Pos.55** | **Pos.56** | **Neg.57** | **Pos.58** | **Neg.59** | **Pos.60** |
| **+** | **+** | **+** | **+** | **+** | **+** | **-** | **+** | **-** | **+** |

Note: “+” indicates a positive sample for influenza A, “-” indicates a negative sample for influenza A.

**Figure S1**


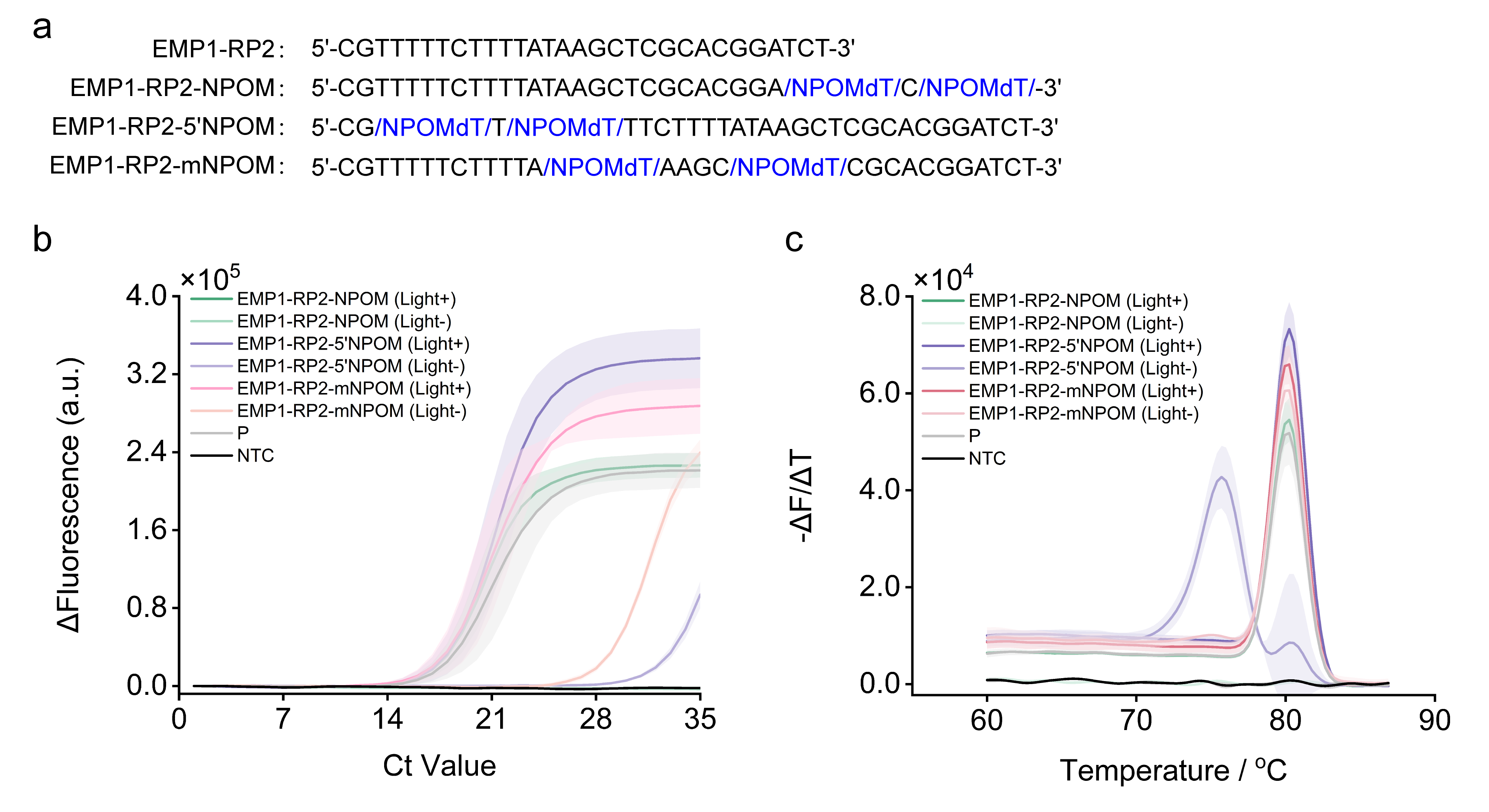


**Figure S1 | Investigation of the effects of modified positions on the conditional control of DNA polymerase activity.** (a) Sequence information of the photocaged reverse primers. The 6-NPOM-caged thymidine was highlighted in blue. The photoactivated qPCR assay for amplifying EMP1 gene (5 pg/μL) using wild forward primer (EMP1-FP2) and various photocaged reverse primers: (b) real-time fluorescence curves, and (c) melting curves. P represents the positive control group, which utilizes a EMP1 plasmid template (5 pg/μL) along with wild forward and reverse primers (EMP1-FP2 and EMP1-RP2) for the conventional qPCR. NTC represents the blank control, using RNase-free water instead of plasmid template. ΔFluorescence (a.u.) represents the difference between the fluorescence value and the initial fluorescence value. Data are represented as mean ± standard error (n = 3 technical replicates).

**Figure S2**


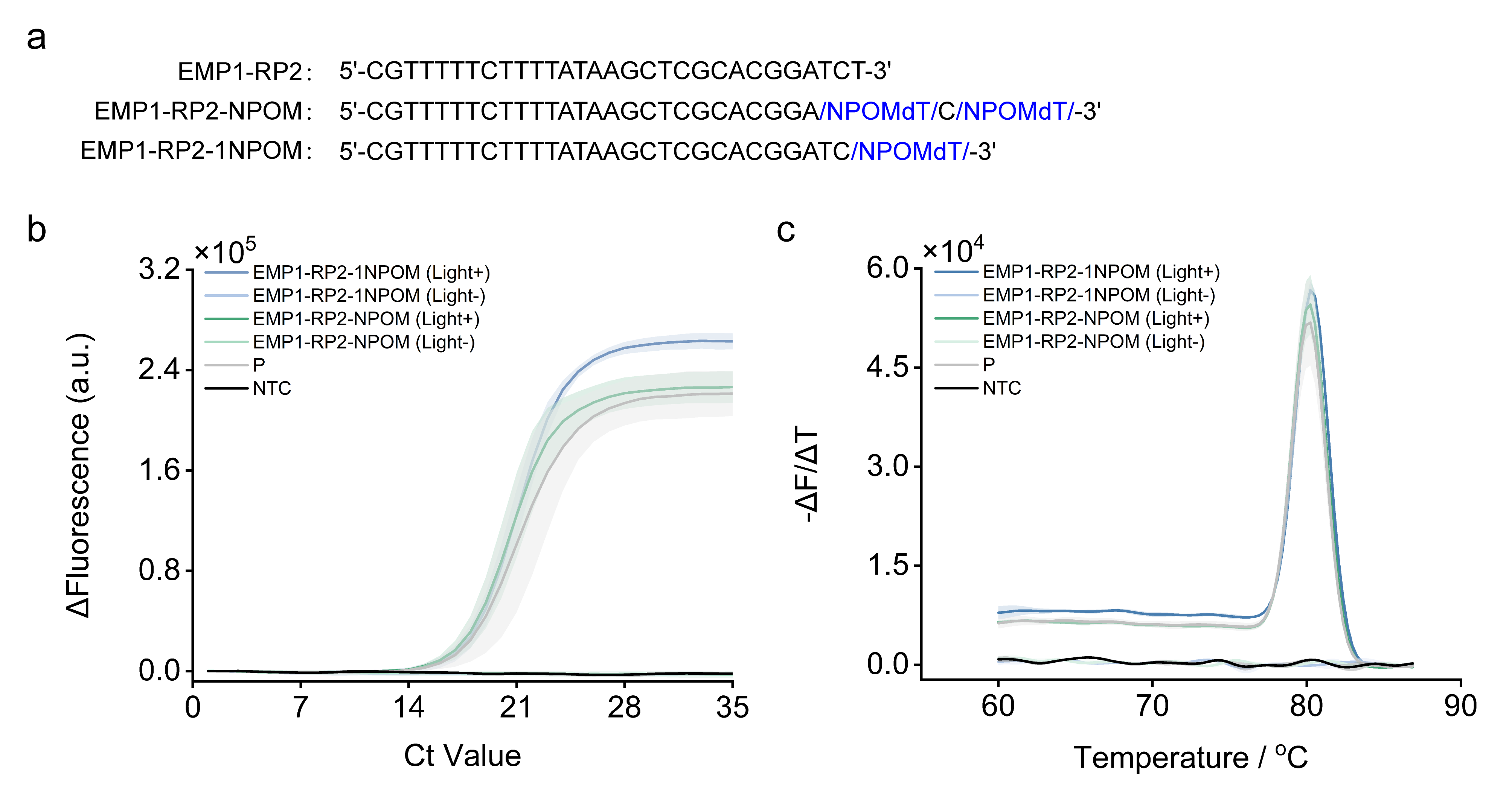


**Figure S2 |** **Investigation of the effect of the number of 3'-end modifications on the conditional control of DNA polymerase activity.** (a) Sequence information of the photocaged reverse primers. The 6-NPOM-caged thymidine was highlighted in blue. The photoactivated qPCR assay for amplifying EMP1 gene (5 pg/μL) using wild forward primer (EMP1-FP2) and various photocaged reverse primers: (b) real-time fluorescence curves, and (c) melting curves. P represents the positive control group, which utilizes a EMP1 plasmid template (5 pg/μL) along with wild forward and reverse primers (EMP1-FP2 and EMP1-RP2) for the conventional qPCR. NTC represents the blank control, using RNase-free water instead of plasmid template. ΔFluorescence (a.u.) represents the difference between the fluorescence value and the initial fluorescence value. Data are represented as mean ± standard error (n = 3 technical replicates).

**Figure S3**


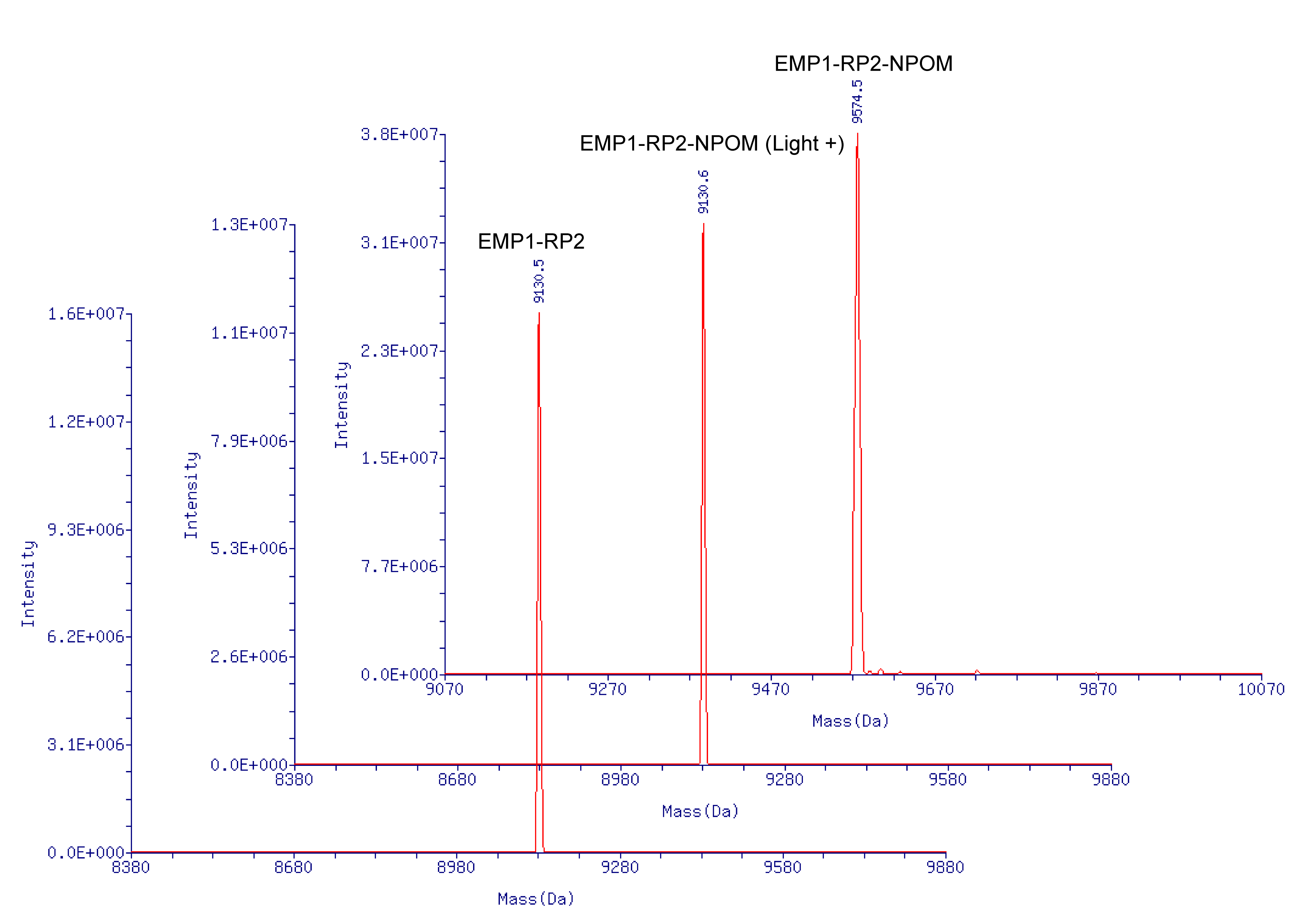


**Figure S3 | The low-resolution mass spectrometry analysis of various primers.** The molecular weight of the wild reverse primer (EMP1-RP2) is 9130.5 Da. The molecular weight of the photocaged reverse primer (EMP1-RP2-NPOM) is 9574.5 Da. The molecular weight of the photocaged reverse primer (EMP1-RP2-NPOM) after exposure to 365 nm UV (30 W) for 50 s is 9130.6 Da.

**Figure S4**


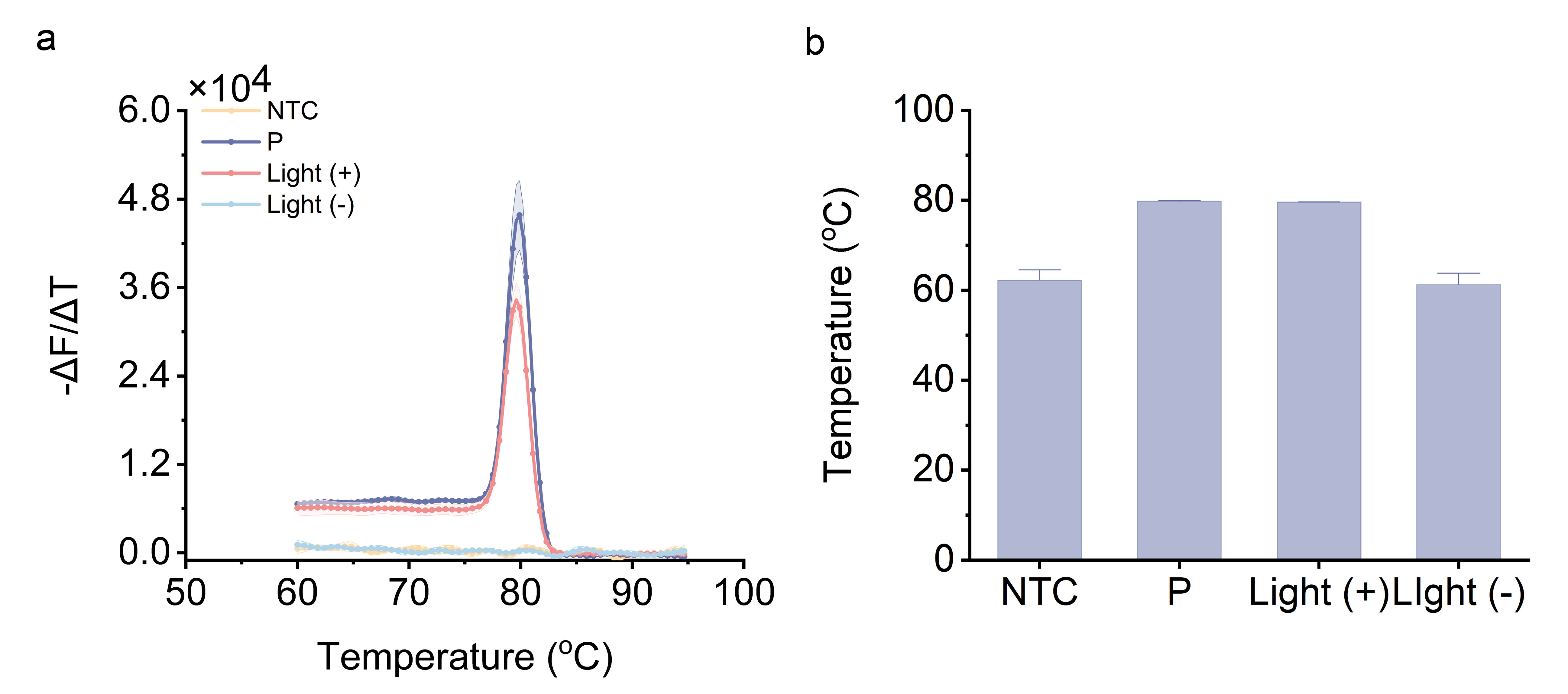


**Figure S4 | The photoactivated qPCR for detecting plasmodium falciparum (EMP1).** Feasibility analysis of the photoactivated qPCR assay for amplifying the EMP1 gene (1 pg/μL) using a wild-type forward primer (EMP1-FP2) and a photocaged reverse primer (EMP1-RP2-NPOM): (a) melting curves, and (b) histogram of melting temperatures. "Light+" indicates that amplification reagent was subjected to light treatment with 365 nm UV lamp (30 W) for 50 s. "Light-" indicates that the reaction was not treated with a 365 nm UV lamp. P represents the positive control group, which utilizes a EMP1 plasmid template (1 pg/μL) along with wild forward and reverse primers (EMP1-FP2 and EMP1-RP2) for the conventional qPCR. NTC represents the blank control, using RNase-free water instead of plasmid template. ΔFluorescence (a.u.) represents the difference between the fluorescence value and the initial fluorescence value. Data are represented as mean ± standard error (n = 3 technical replicates).

**Figure S5**


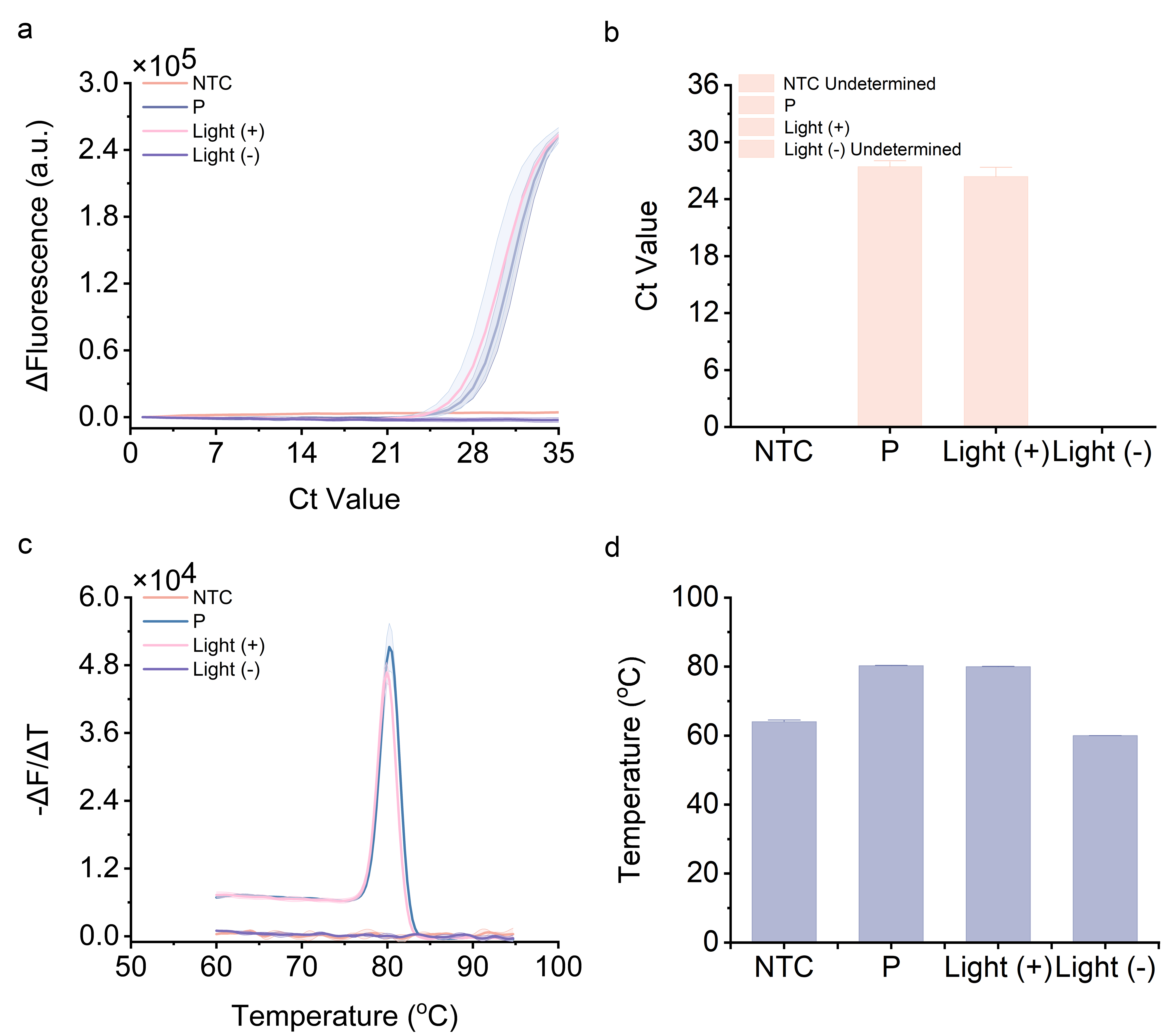


**Figure S5 |** **The photoactivated qPCR for detecting flavivirus dengue virus (****DENV).** Feasibility analysis of the photoactivated qPCR assay for amplifying the DENV gene (1 pg/μL) using a wild-type forward primer (DENV-FP) and a photocaged reverse primer (DENV-RP-NPOM): (a) real-time fluorescence curves, (b) histogram of cycle threshold values, (c) melting curves, and (d) histogram of melting temperatures. "Light+" indicates that reaction was subjected to light treatment with 365 nm UV lamp (30 W) for 50 s. "Light-" indicates that the reaction was not treated with a 365 nm UV lamp. P represents the positive control group, which utilizes a DENV plasmid template (1 pg/μL) along with wild forward and reverse primers (DENV-FP and DENV-RP) for the conventional qPCR. NTC represents the blank control, using RNase-free water instead of plasmid template. ΔFluorescence (a.u.) represents the difference between the fluorescence value and the initial fluorescence value. Data are represented as mean ± standard error (n = 3 technical replicates).

**Figure S6**

**
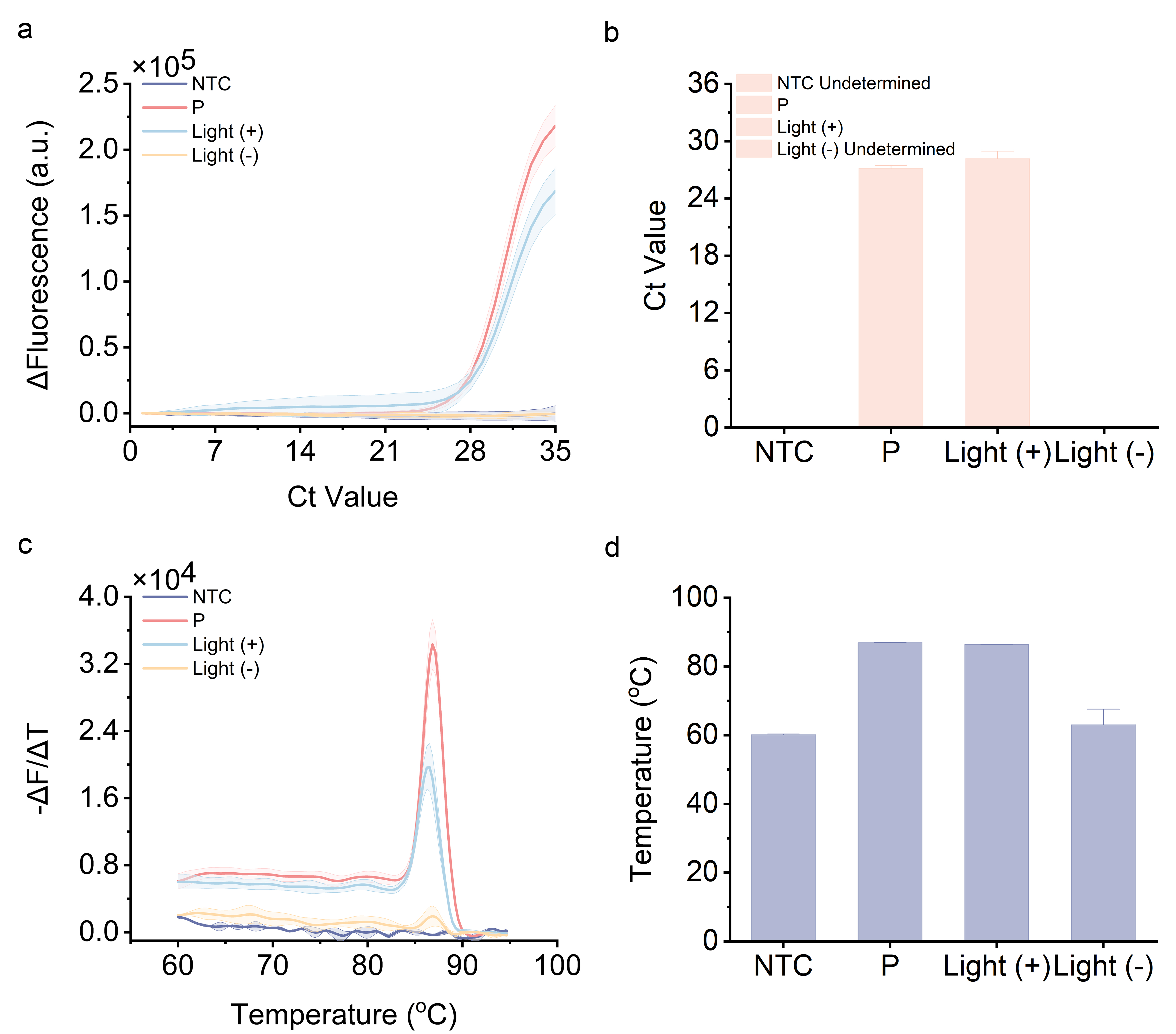
**

**Figure S6 | The photoactivated qPCR for detecting** **Chikungunya virus (****CHIKV).** Feasibility analysis of the photoactivated qPCR assay for amplifying the CHIKV gene (1 pg/μL) using a wild-type forward primer (CHIKV-FP3) and a photocaged reverse primer (CHIKV-RP1-NPOM): (a) real-time fluorescence curves, (b) histogram of cycle threshold values, (c) melting curves, and (d) histogram of melting temperatures. "Light+" indicates that reaction was subjected to light treatment with 365 nm UV lamp (30 W) for 50 s. "Light-" indicates that the reaction was not treated with a 365 nm UV lamp. P represents the positive control group, which utilizes a CHIKV plasmid template (1 pg/μL) along with wild forward and reverse primers (CHIKV-FP3 and CHIKV-RP1) for the conventional qPCR. NTC represents the blank control, using RNase-free water instead of plasmid template. ΔFluorescence (a.u.) represents the difference between the fluorescence value and the initial fluorescence value. Data are represented as mean ± standard error (n = 3 technical replicates).

**Figure S7**


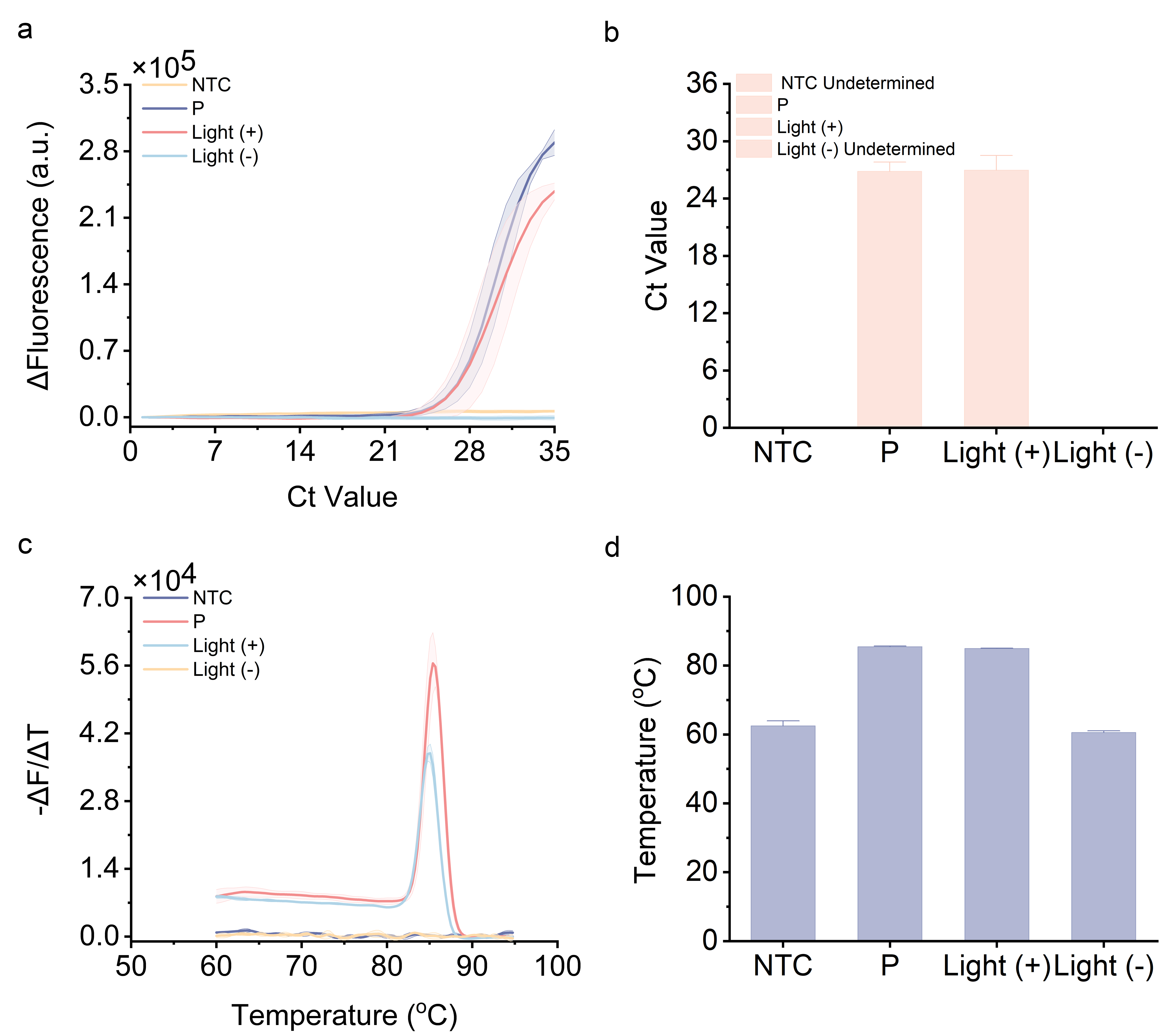


**Figure S7 | The photoactivated qPCR for detecting Zika virus (****ZIKV).** Feasibility analysis of the photoactivated qPCR assay for amplifying the ZIKV gene (1 pg/μL) using a wild-type forward primer (ZIKV-FP3) and a photocaged reverse primer (ZIKV-RP3-NPOM): (a) real-time fluorescence curves, (b) histogram of cycle threshold values, (c) melting curves, and (d) histogram of melting temperatures. "Light+" indicates that reaction was subjected to light treatment with 365 nm UV lamp (30 W) for 50 s. "Light-" indicates that the reaction was not treated with a 365 nm UV lamp. P represents the positive control group, which utilizes a ZIKV plasmid template (1 pg/μL) along with wild forward and reverse primers (ZIKV-FP3 and ZIKV-RP3) for the conventional qPCR. NTC represents the blank control, using RNase-free water instead of plasmid template. ΔFluorescence (a.u.) represents the difference between the fluorescence value and the initial fluorescence value. Data are represented as mean ± standard error (n = 3 technical replicates).

**Figure S8**

**
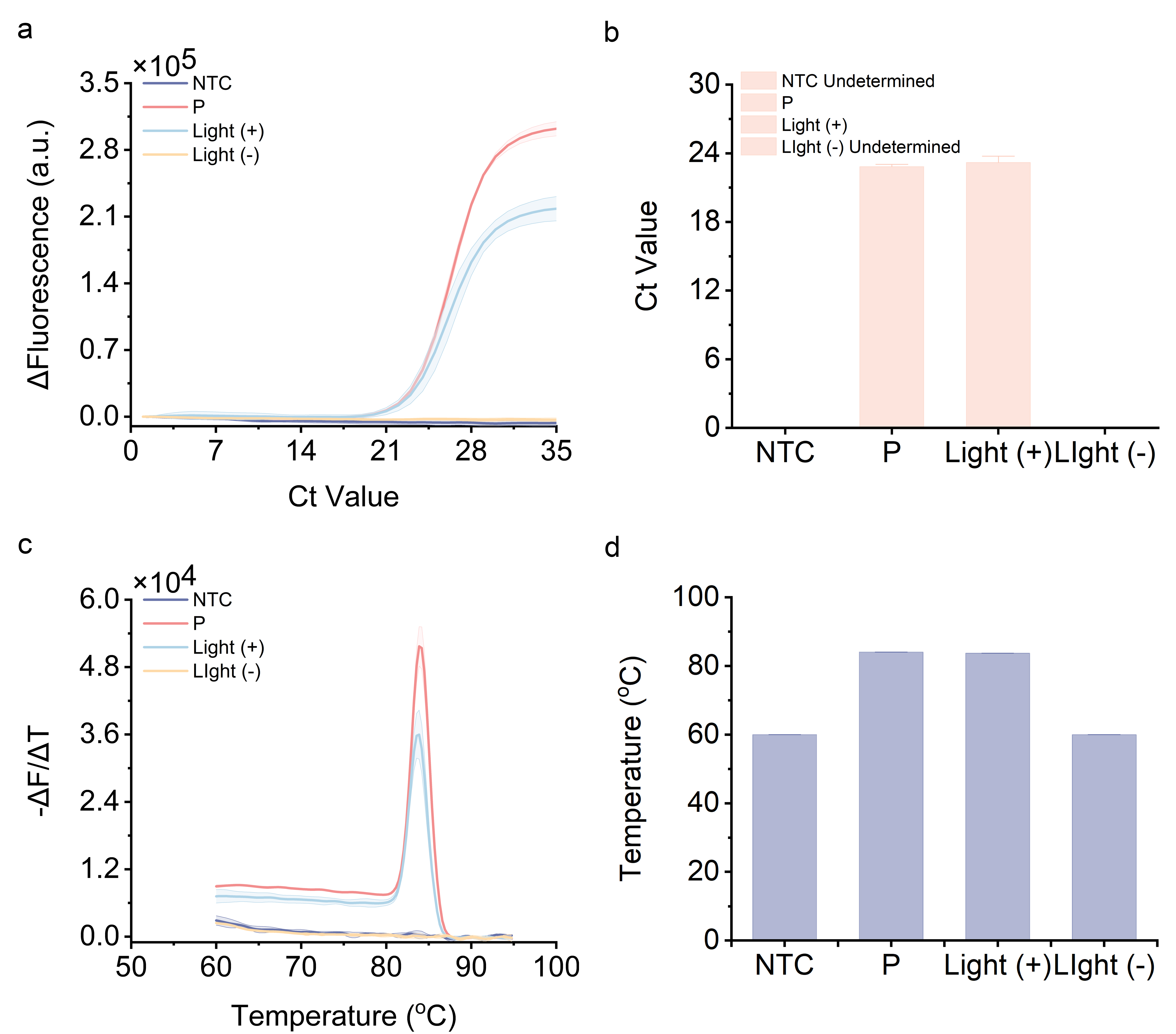
**

**Figure S8 | The photoactivated qPCR for detecting influenza A virus (****H1N1).** Feasibility analysis of the photoactivated qPCR assay for amplifying the H1N1 gene (1 pg/μL) using a wild-type forward primer (H1N1-FP1) and a photocaged reverse primer (H1N1-RP4-NPOM): (a) real-time fluorescence curves, (b) histogram of cycle threshold values, (c) melting curves, and (d) histogram of melting temperatures. "Light+" indicates that reaction was subjected to light treatment with 365 nm UV lamp (30 W) for 50 s. "Light-" indicates that the reaction was not treated with a 365 nm UV lamp. P represents the positive control group, which utilizes a H1N1 plasmid template (1 pg/μL) along with wild forward and reverse primers (H1N1-FP1 and H1N1-RP4) for the conventional qPCR. NTC represents the blank control, using RNase-free water instead of plasmid template. ΔFluorescence (a.u.) represents the difference between the fluorescence value and the initial fluorescence value. Data are represented as mean ± standard error (n = 3 technical replicates).

**Figure S9**


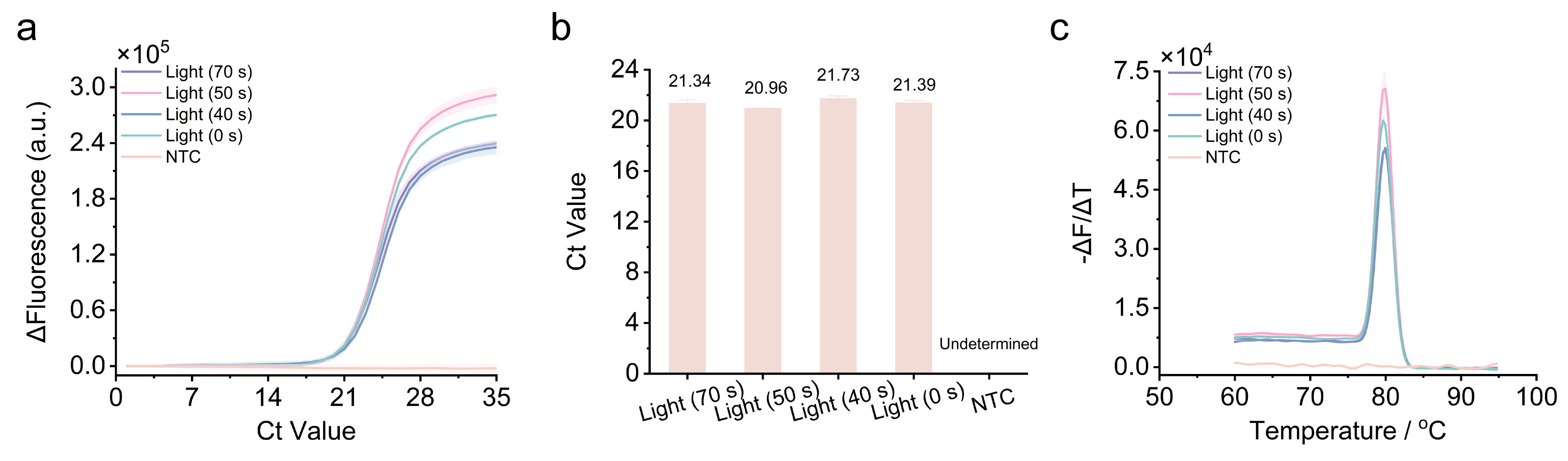


**Figure S9 | The influence of UV irradiation on templates.** Dose‑response analysis of the UV irradiation on the EMP1 template (2.5 pg/μL) using a wild-type forward primer (EMP1-FP2) and reverse primer (EMP1-RP2): (a) real-time fluorescence curves, (b) histogram of cycle threshold values, (c) melting curves. The EMP1 template was subjected to UV light treatment with a 365 nm UV lamp (30 W) for different durations (0 - 70 s), and then a conventional qPCR was performed. NTC represents the blank control, using RNase-free water instead of plasmid template. ΔFluorescence (a.u.) represents the difference between the fluorescence value and the initial fluorescence value. Data are represented as mean ± standard error (n = 3 technical replicates).

**Figure S10**


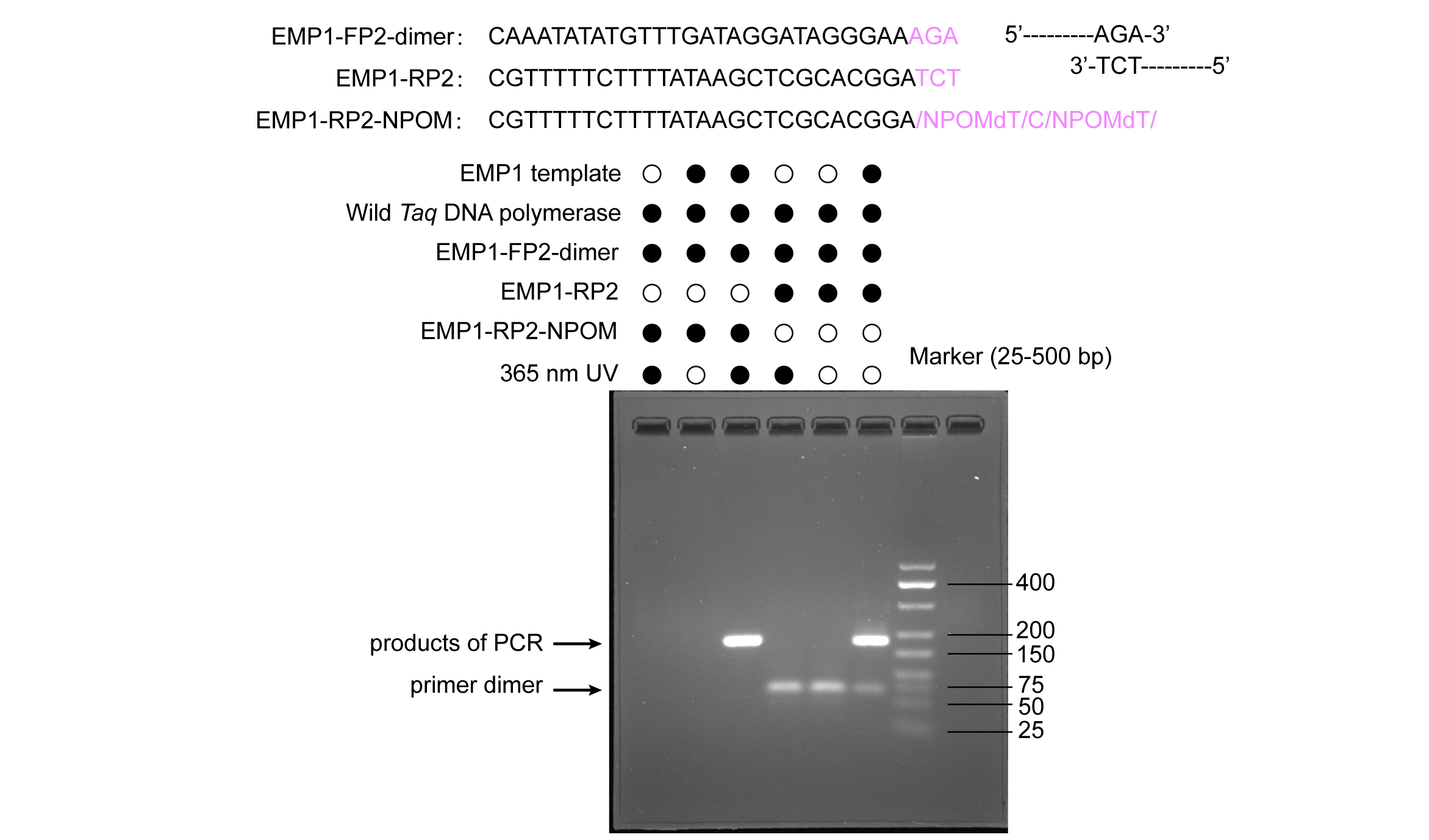


**Figure S10 |** **Evaluation of the effect of photocaged primer on preventing non-specific primer dimer formation.** Agarose gel electrophoresis analysis of the PCR products resulting from the amplification of a 162-bp fragment from the EMP1 gene (1 pg/μL) using 0.4 μM wild primers (EMP1-RP2 and EMP1-FP2-dimer) or photocaged primers (EMP1-RP2-NPOM and EMP1-FP2-dimer). The three complementary bases at the 3'-end of the primer are highlighted in pink. The wild *Taq* DNA polymerase indicates non-hot-start *Taq* DNA polymerase. "Light+" indicates that amplification reagent was subjected to light treatment with 365 nm UV lamp (30 W) for 50 s.

**Figure S11**


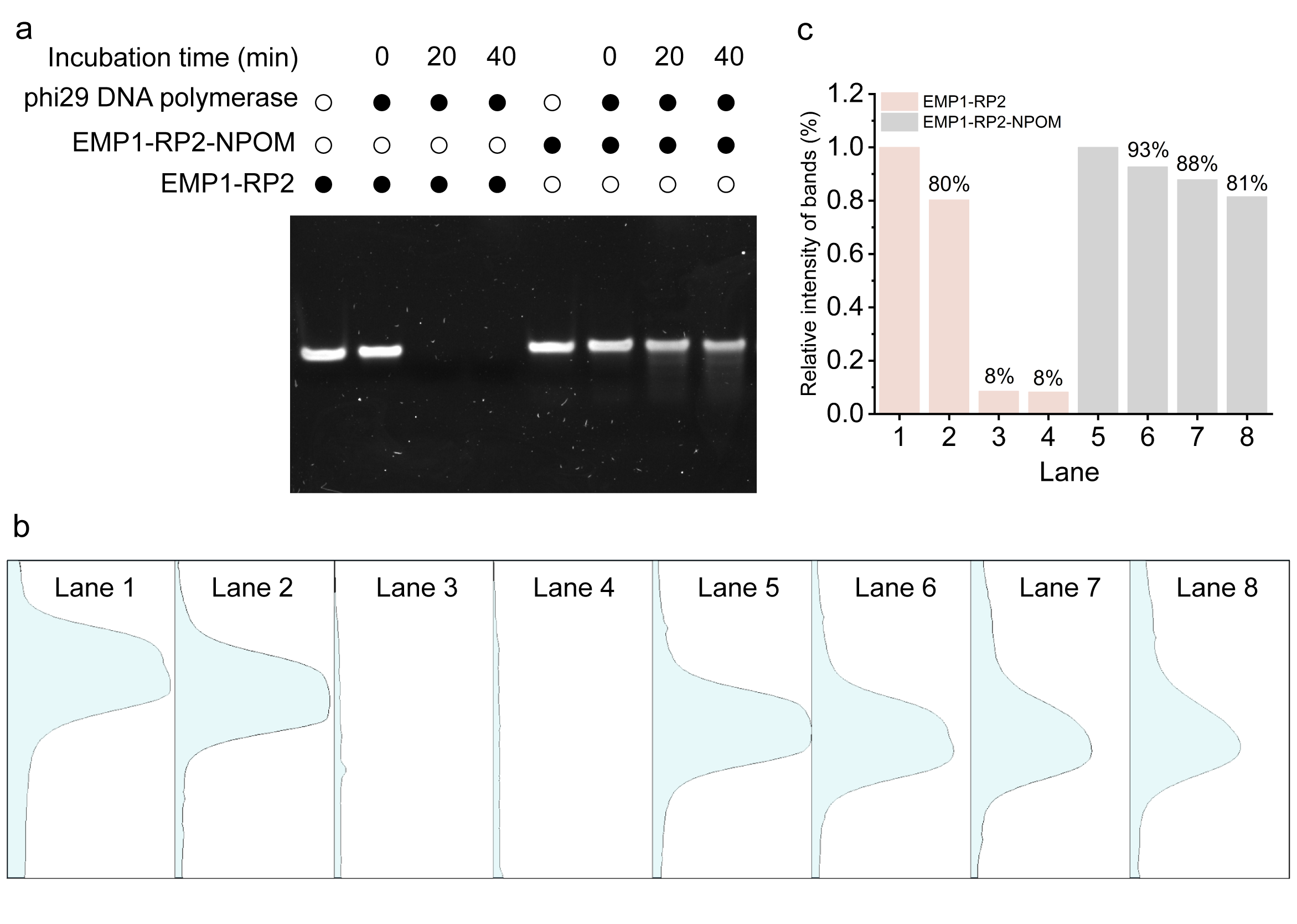


**Figure S11 |** **Evaluation of exonuclease resistance of photocaged primer against phi29 DNA polymerase.** (a) PAGE analysis of the products resulting from degradation experiment. (b) The curve graph of quantification of band intensity. (c) The percentage of intensity of bands relative to the first band in each group. Lane 1 contains 10 μL of 1 μM EMP1-RP2. Lane 5 contains 10 μL of 1 μM EMP1-RP2-NPOM. The primer degradation assay was performed in a 20 μL of system containing 4 U of phi29 DNA polymerase and 1 μM primer (EMP1-RP2 or EMP1-RP2-NPOM).

**Figure S12**


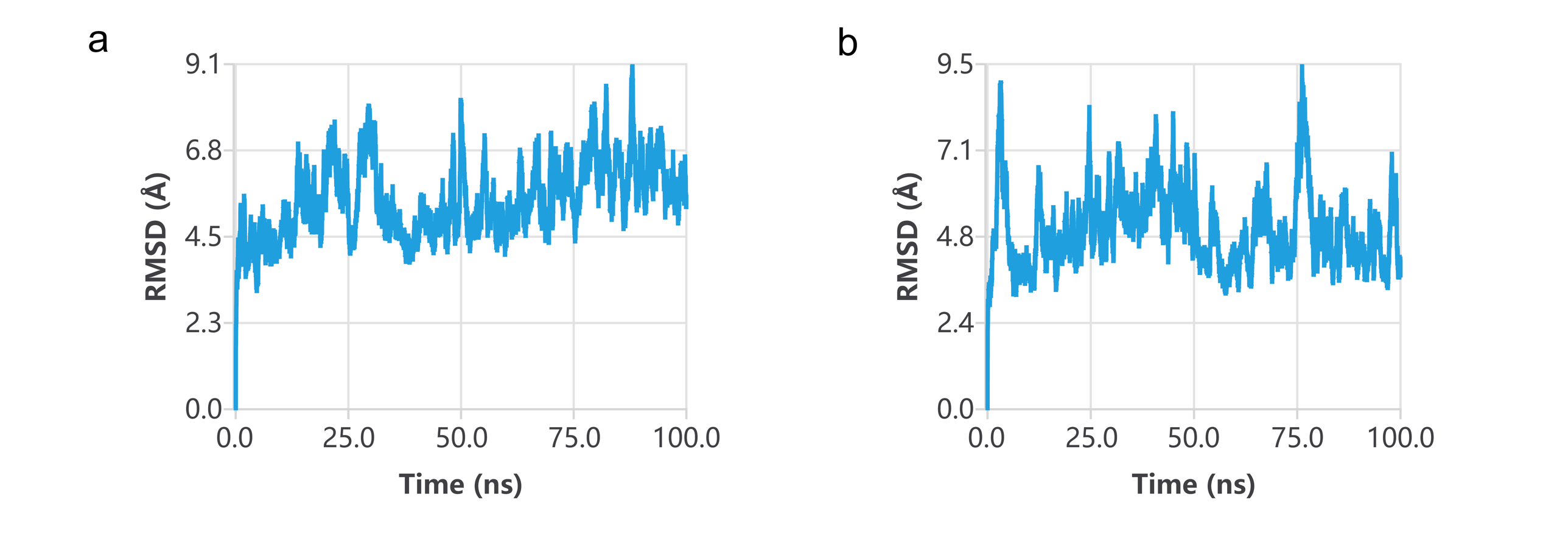


**Figure S12 | The** **root means square deviation (RMSD) of template-primer-polymerase complex versus simulation time.** (a) The RMSD of mismatched primer (EMP1-RP2-M3)-template-polymerase complex versus simulation time. (b) The RMSD of photocaged primer (EMP1-RP2-NPOM)-template-polymerase complex versus simulation time. The root-mean-square deviation (RMSD) can provide insights into the motion of the complexes. A larger RMSD value indicates greater fluctuations, suggesting more turbulent motion. Conversely, a smaller RMSD value corresponds to smoother motion.

The RMSD of both types of complexes shows minimal fluctuations, remaining within the 10 Å range without significant deviation. This indicates that the movement of both complexes is relatively stable and that the system remains intact. The volatility and height of the RMSD of photocaged primer (EMP1-RP2-NPOM)-template-polymerase complex are greater than those of mismatched primer (EMP1-RP2-M3)-template-polymerase complex, indicating that the former displays a more pronounced degree of motion.

**Figure S13**


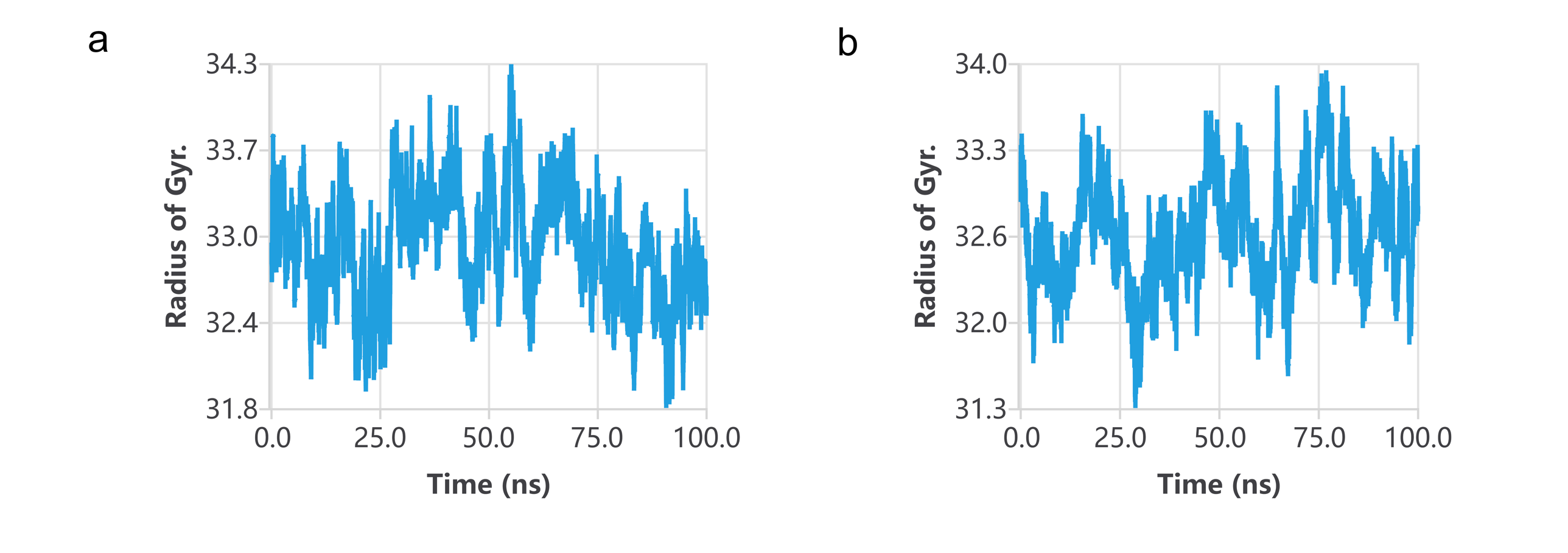


**Figure S13 | The RoG (****radius of gyration) of template-primer-polymerase complex versus simulation time.** (a) The RoG of mismatched primer (EMP1-RP2-M3)-template-polymerase complex versus simulation time; (b) The RoG of photocaged primer (EMP1-RP2-NPOM)-template-polymerase complex versus simulation time. The RoG measures a system's overall compactness, with lower values indicating greater compactness. The RoG of mismatched primer (EMP1-RP2-M3)-template-polymerase complex exhibits fluctuations between 31.8 and 34.3 Å. The RoG of photocaged primer (EMP1-RP2-NPOM)-template-polymerase complex exhibits fluctuations between 31.3 and 34.0 Å. The RoG of photocaged primer (EMP1-RP2-NPOM)-template-polymerase complex (2.7) is higher than that of mismatched primer (EMP1-RP2-M3)-template-polymerase complex (2.5), indicating that the former has greater potential for fluctuation.

**Figure S14**


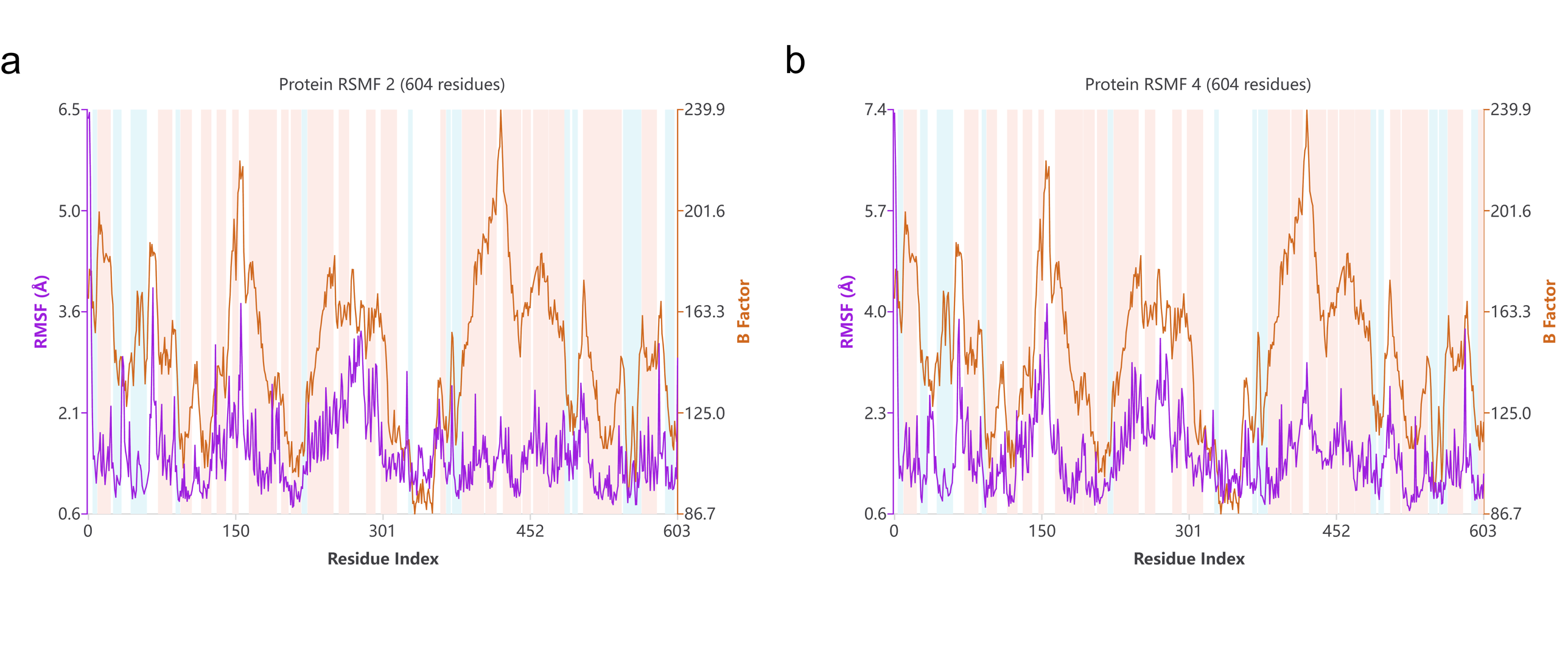


**Figure S14 | The root means square fluctuation (RMSF) of a polymerase upon binding to different primer-template complexes as a function of time.** (a) The RMSF of polymerase upon binding to mismatched primer (EMP1-RP2-M3))-template complex. (b) The RMSF of polymerase upon binding to photocaged primer (EMP1-RP2-NPOM)-template complex. The RMSF reflects the magnitude of a polymerase's flexibility during molecular dynamics simulations. The RMSF is represented by a color gradient, with a red background indicating helices, blue indicating β-sheets, and white indicating loops. The RMSF of polymerase bound to mismatched primer (EMP1-RP2-M3)-template complex is generally low, particularly when compared to the B factor of the two complexes. Conversely, the RMSF of polymerase bound to photocaged primer (EMP1-RP2-NPOM)-template complex is higher overall, especially within the 300-500 sequence segment. Furthermore, regarding the secondary structure, there is minimal distinction between the polymerase bound to different primer-template complexes. This suggests that the polymerase exhibits greater flexibility in the presence of the photocaged primer (EMP1-RP2-NPOM)-template complex.

**Figure S15**


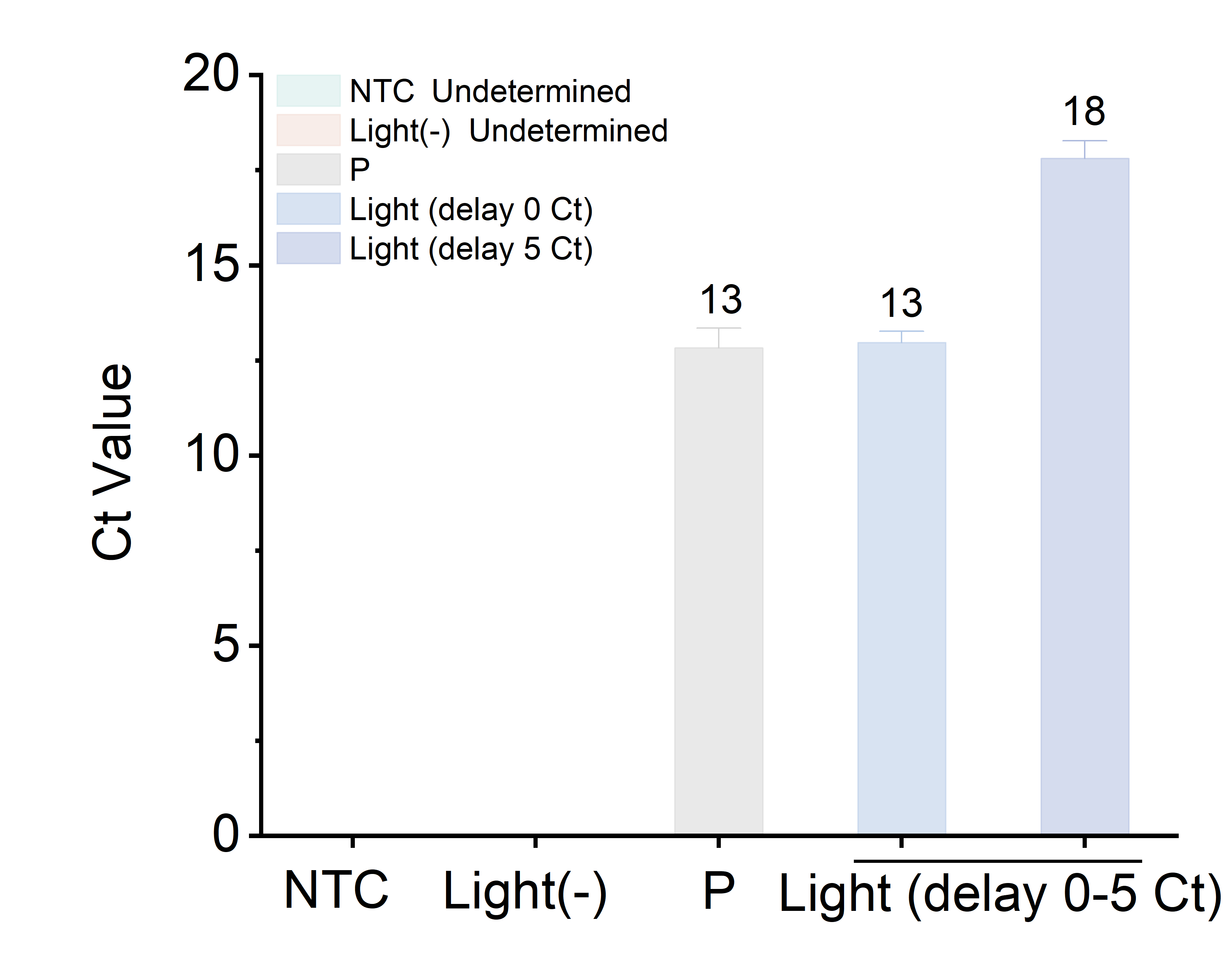


**Figure S15 |** **Cycle threshold (Ct) value of photoactivated qPCR for amplifying CHIKV plasmid (1 ng/μL) using wild forward primer (CHIKV-FP3) and photocaged reverse primer (CHIKV-RP1-NPOM).** "Light+" indicates that amplification reagent was subjected to light treatment with 365 nm UV lamp (30 W) for 50 s. "Light-" indicates that the reaction was not treated with a 365 nm UV lamp. P represents the positive control group, using CHIKV plasmid template (1 ng/μL) with wild forward and reverse primers (CHIKV-FP3 and CHIKV-RP1) for conventional qPCR. NTC represents the blank control, using RNase-free water instead of plasmid template.

**Figure S16**

Previous studies have demonstrated that the first step of primer extension is the establishment of primer-template complementarity. If the 3'-end of the primer does not match with the template, DNA polymerase cannot initiate DNA synthesis. Component deletion experiments revealed that the reverse primers are closely related to the generation of fluorescence signal (Figure S16b). We therefore designed a series of mismatched nucleobases on the reverse primer to assess it impact on primer extension. Two mismatched nucleobases at the 3'-end of the reverse primer were sufficient to inhibit the amplification, regardless of the strength of the mismatched nucleobases (Figure S16a and c-d). Accordingly, we synthesized a photocaged reverse primer with two 6-NPOM-caged thymine at the 3'-end.


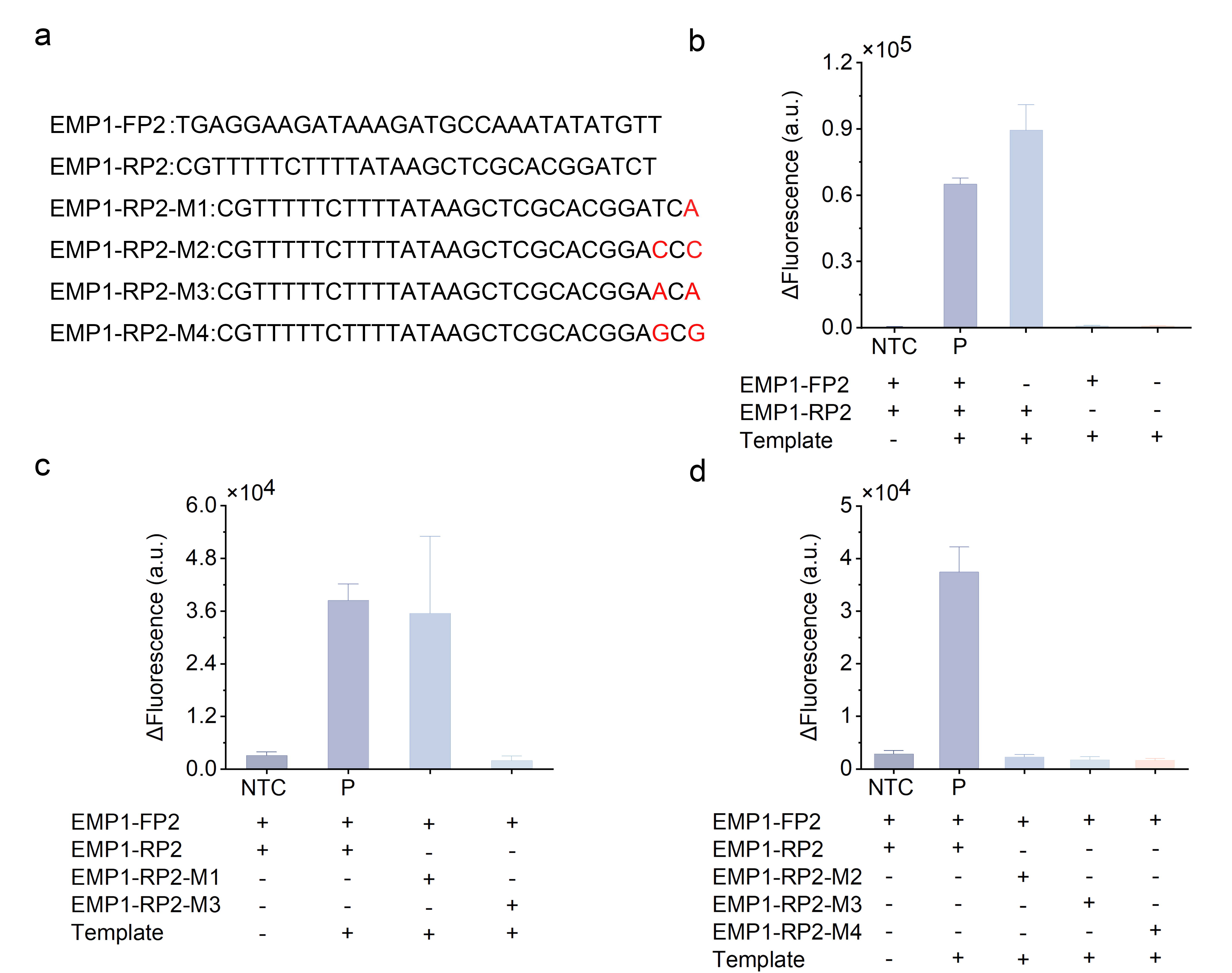


**Figure S16 | Design and validation of mismatched primers.** (a) The figure depicts the sequence of wild and mismatched primers, wherein the bases indicated in red represent mismatched nucleobases. (b) Primers associated with fluorescence signal generation were screened by component deletion experiments. These experiments involved adding either the forward primer (EMP1-FP2), the reverse primer (EMP1-RP2), both, or neither into the conventional RPA system. (c) Investigation of the effect of the number of mismatched nucleobases on the conventional RPA system. Amplification was conducted using wild forward primer (EMP1-FP2) and two reverse primers with different mismatched numbers (EMP1-RP2-M1 and EMP1-RP2-M3 in a). (d) Evaluation of the impact of mismatched strength on the conventional RPA. Amplification was conducted using wild forward primer (EMP1-FP2) and three reverse primers with different mismatched strengths (EMP1-RP2-M2, EMP1-RP2-M3, and EMP1-RP2-M4 in a). Mismatch intensity order: G:A > A:A > A:C. P represents the positive control group, using a plasmid template (EMP1 plasmid) with wild forward and reverse primers (EMP1-FP2 and EMP1-RP2) for the conventional RPA. NTC represents the blank control, using RNase-free water instead of plasmid template. ΔFluorescence (a.u.) represents the difference between the fluorescence value and the initial fluorescence value. Data are represented as mean ± standard error (n = 3 technical replicates).

**Figure S17**

We noted that inappropriate lighting conditions could lead to an increase in sample temperature and affect the activity of the non-thermophilic protease, thereby reducing the efficiency of amplification (Figure S17-18). Subsequently, we optimized the lighting conditions by investigating the relationship between temperature, light distance, and exposure time (Figure S19-20). The results indicated that light irradiation at the distance of 4 cm or 5 cm maintained the temperature between 30-45 °C. When the sample was irradiated with 365 nm UV lamp (30 W) for 50 s at a distance of 4 cm, the amplification efficiency was the highest, making it the optimal lighting condition for subsequent experiments (Figure S20).


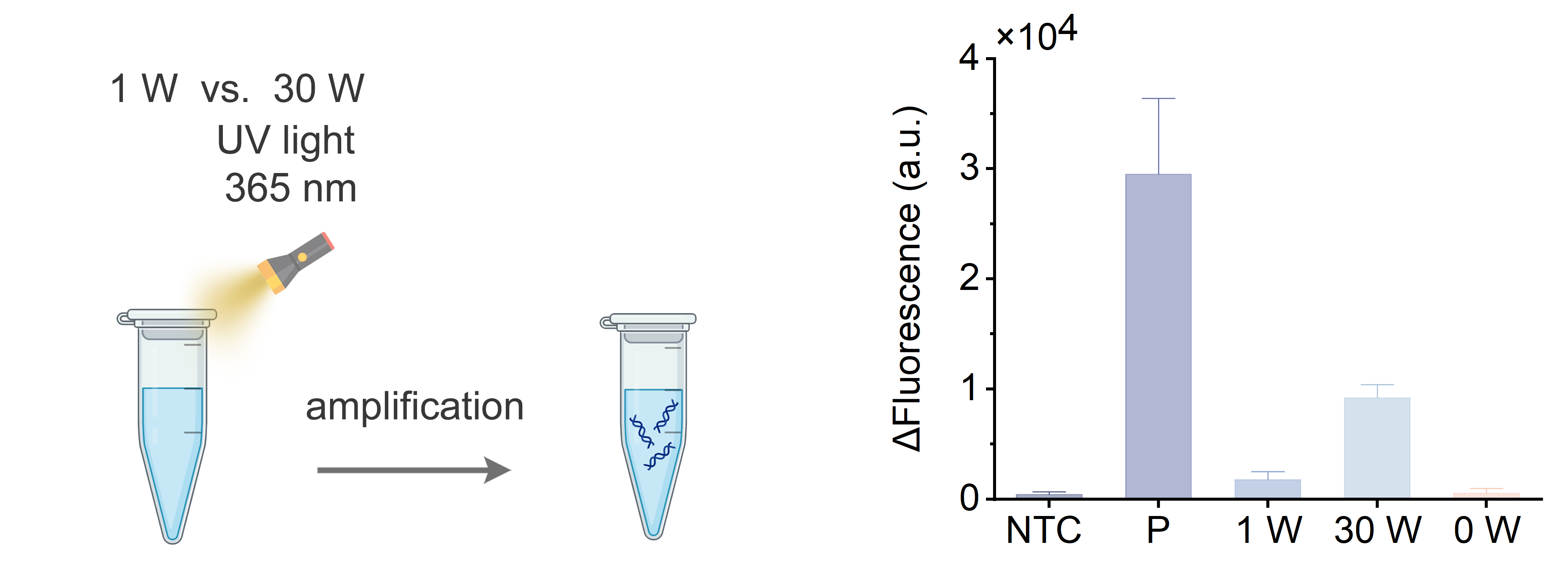


**Figure S17 | Evaluating the effect of different intensities of UV light on the amplification efficiency using a photocaged primer.** The Light-start RPA reaction using the wild forward primer (EMP1-FP2) and the photocaged reverse primer (EMP1-RP2-NPOM) is performed in parallel, except that the reactions are irradiated with two different powers of a 365 nm UV lamp for 30 s, respectively. P represents the positive control group, using a plasmid template (EMP1 plasmid) with wild forward and reverse primers (EMP1-FP2 and EMP1-RP2) for the conventional RPA system. NTC represents the blank control, using RNase-free water instead of plasmid template. ΔFluorescence (a.u.) represents the difference between the fluorescence value and the initial fluorescence value. Data are represented as mean ± standard error (n = 3 technical replicates).

**Figure S18**


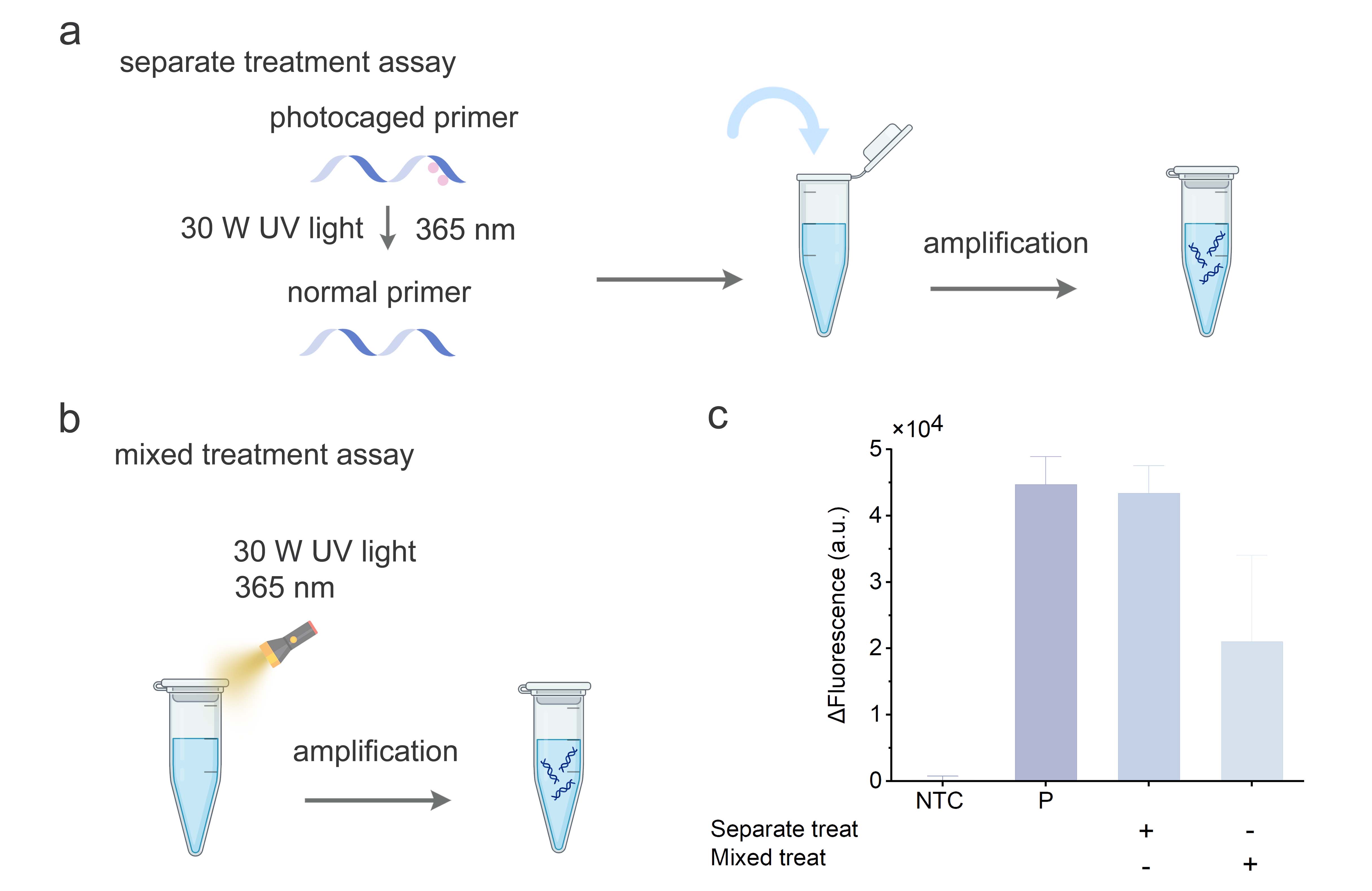


**Figure S18 | Comparing the effect of irradiating the photocaged primer separately and irradiating the Light-start RPA reaction directly on the amplification efficiency.** (a) Schematic diagram of the separate treatment assay. The separate treatment assay refers to irradiating the photocaged reverse primer (EMP1-RP2-NPOM) for 30 s before adding it to the amplification system. (b) Schematic diagram of mixed treatment assay. The mixed treatment assay refers to the amplification system being directly irradiated for 30 s. A 365 nm UV lamp (30 W) was used for the experiment. (c) Comparing the effect of irradiating the photocaged primer separately and irradiating the amplification system directly on the amplification efficiency. P represents the positive control group, using a plasmid template (EMP1 plasmid) with wild forward and reverse primers (EMP1-FP2 and EMP1-RP2) for the conventional RPA system. NTC represents the blank control, using RNase-free water instead of plasmid template. ΔFluorescence (a.u.) represents the difference between the fluorescence value and the initial fluorescence value. Data are represented as mean ± standard error (n = 3 technical replicates).

**Figure S19**


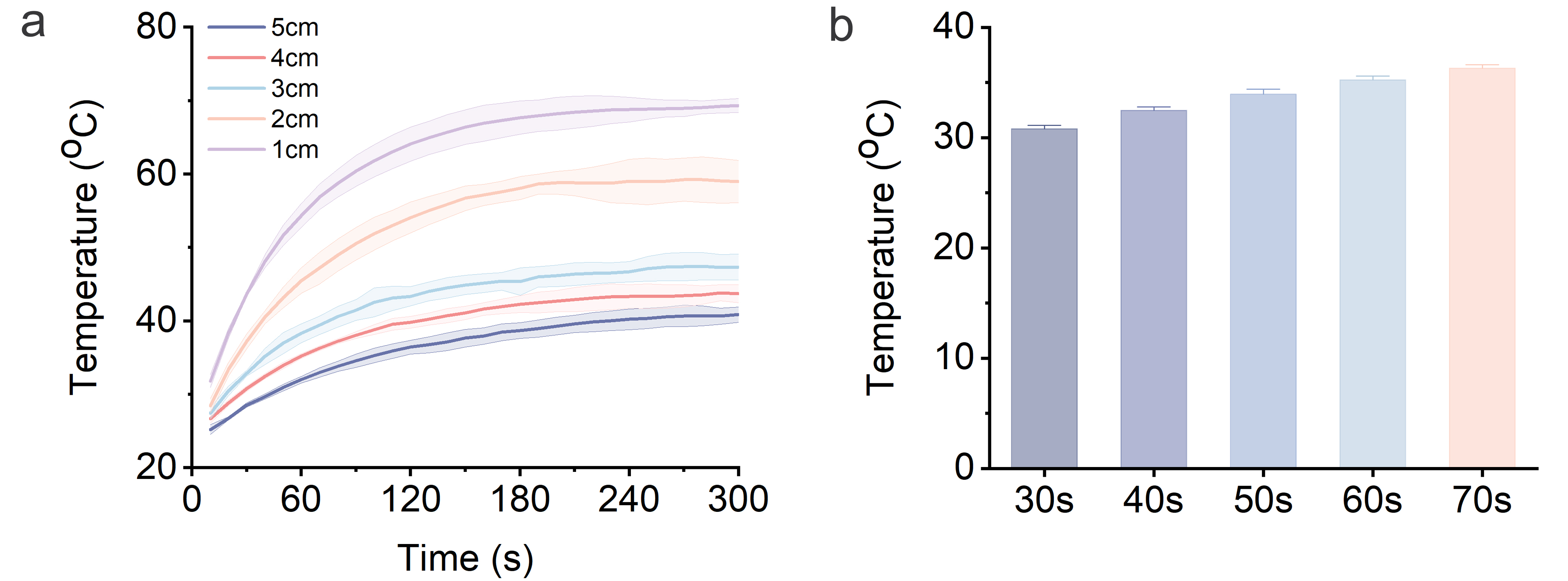


**Figure S19 | Time-distance-temperature graph.** (a) Micro-thermometer was used to record the temperature changes of the conventional RPA with light distance and time. (b) Temperature-time histogram at a distance of 4 cm. The distance between the 365 nm UV lamp and the PCR tube was maintained at 4 cm, and a micro-thermometer was used to measure the temperature of the conventional RPA at varying irradiation times. A 365 nm UV lamp (30 W) was used for the experiment. Data are represented as mean ± standard error (n = 3 technical replicates).

**Figure S20**


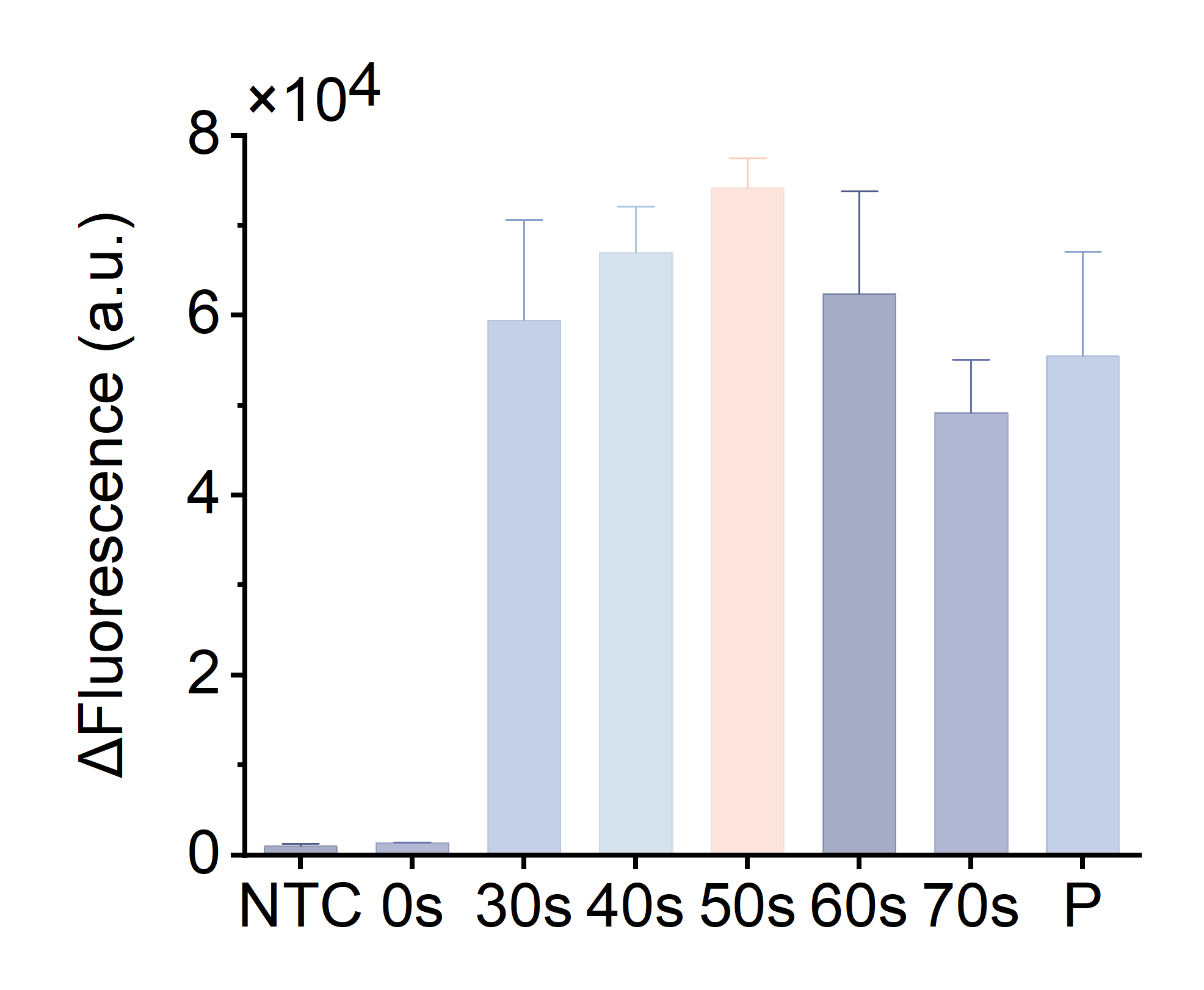


**Figure S20 | Assessing the effect of light activation time on amplification efficiency.** The Light-start RPA was treated with a 365 nm UV lamp (30 W) for varying periods. Subsequently, the system was amplified at 39 °C. The distance between the 365 nm UV lamp (30 W) and the PCR tube was maintained at 4 cm. P represents the positive control group, using a plasmid template (EMP1 plasmid) with wild forward and reverse primers (EMP1-FP2 and EMP1-RP2) for the conventional RPA. NTC represents the blank control, using RNase-free water instead of plasmid template. ΔFluorescence (a.u.) represents the difference between the fluorescence value and the initial fluorescence value. Data are represented as mean ± standard error (n = 3 technical replicates).

**Figure S21**


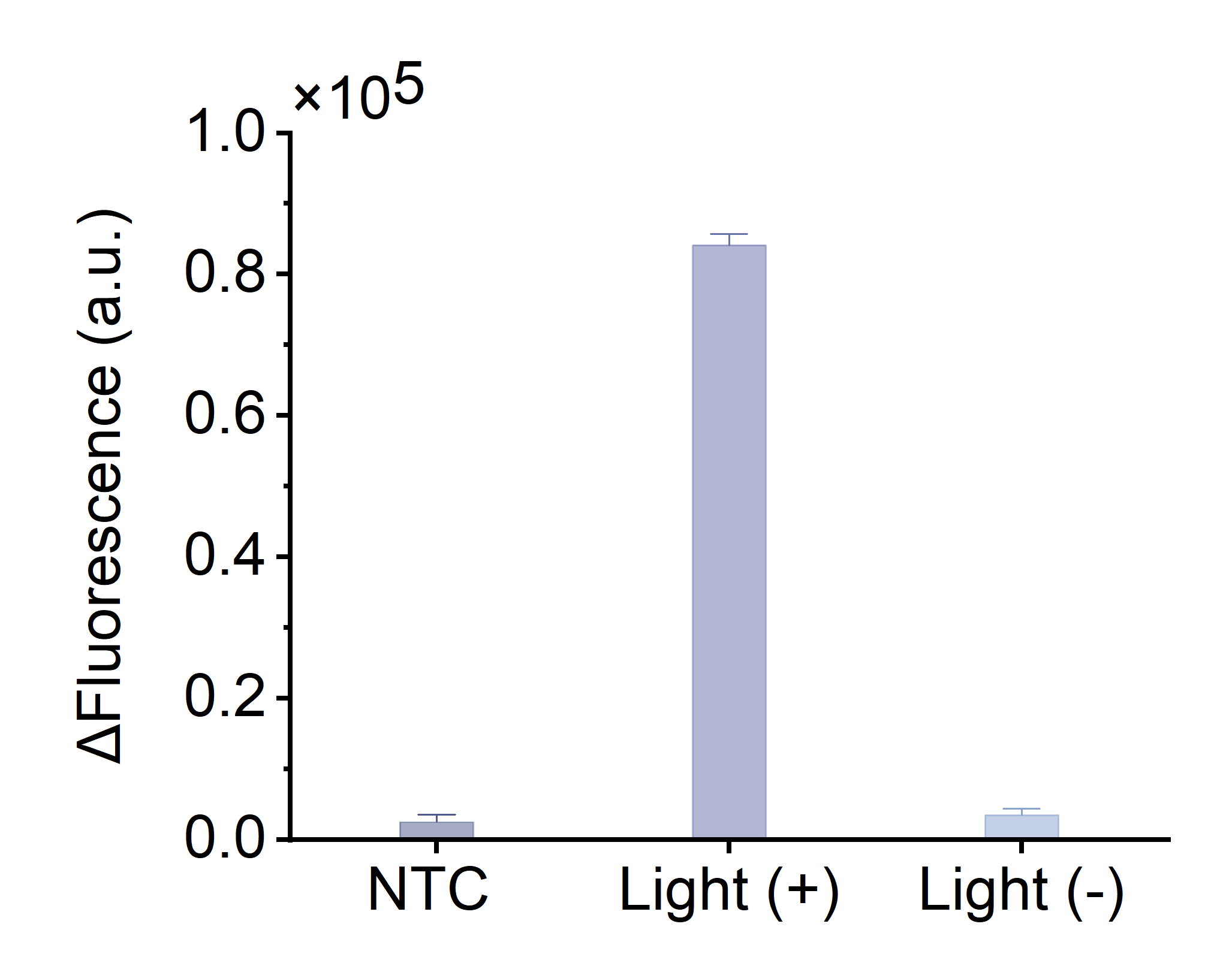


**Figure S21 | The stability of photocaged primers.** The photocaged reverse primer (CHIKV-RP1-NPOM) was stored at room temperature under ambient light for 24 h prior to their addition to the Light-start RPA. The amplification system was then activated by illumination with a 365 nm UV lamp (30 W) for 50 s (light +). "Light-" indicates that the sample was not treated with a 365 nm UV lamp. NTC represents the blank control, using RNase-free water instead of plasmid template. ΔFluorescence (a.u.) represents the difference between the fluorescence value and the initial fluorescence value. Data are represented as mean ± standard error (n = 3 technical replicates).

**Figure S22**

The droplet generator is fabricated by fixing a polyethylene needle inside a common microcentrifuge tube, where the needle serves as a container for the Light-start RPA reagent. (Figure S22a). The generator is then placed in a 1.5 mL centrifuge tube, followed by a bench centrifuge, where centrifugal force drives the aqueous reagent through the needle to form droplets. The droplets rise to the top interface due to the higher density of the fluorocarbon oil (Figure S22b).


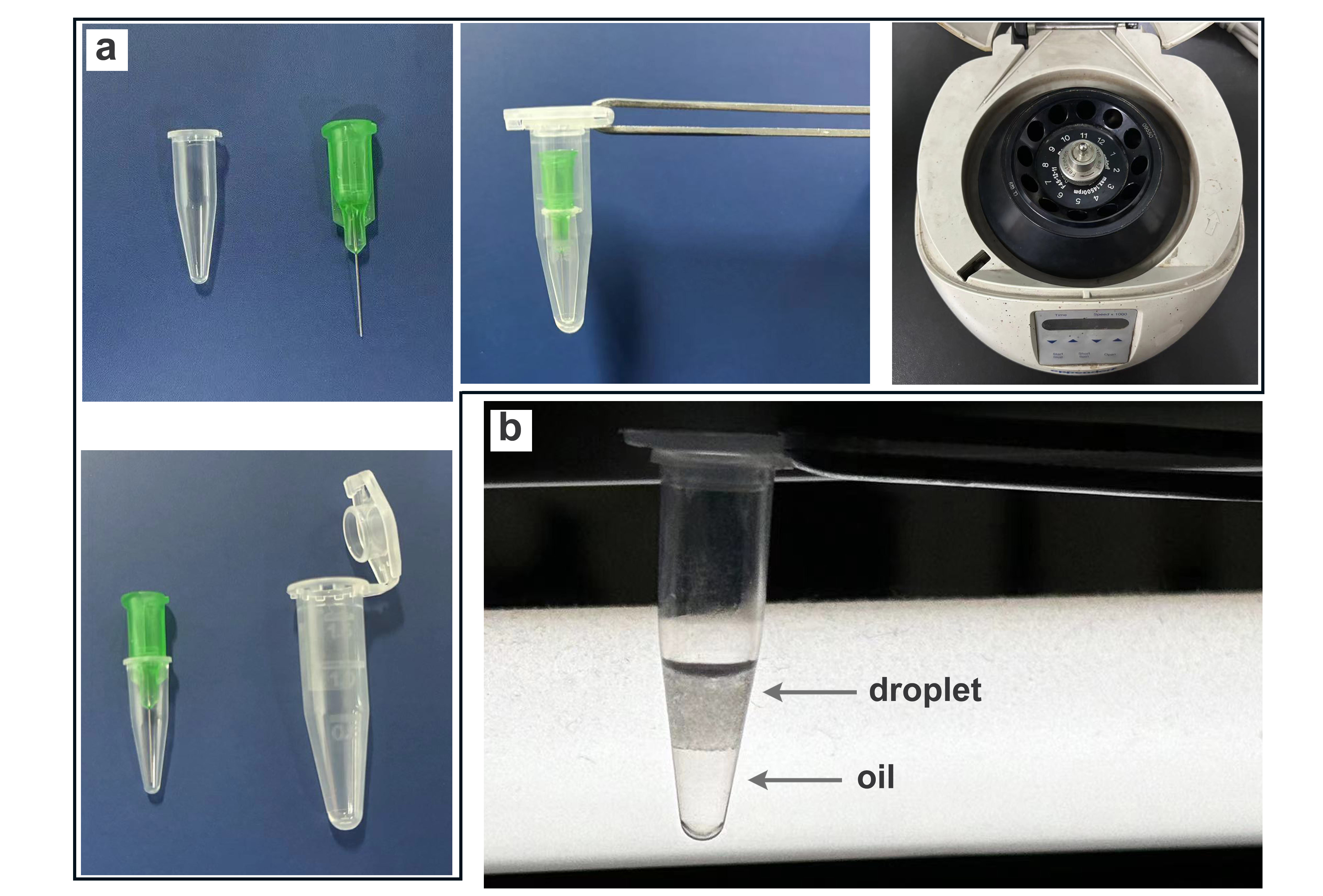


**Figure S22 | Image of the droplet generator.** (a) Actual picture of centrifugal-driven droplet generator. (b) After the droplets are produced by centrifugation, they are transferred to a 1.5 mL centrifuge tube for imaging. Upper layer: droplets, the lower layer: fluorocarbon oil.

**Figure S23**

To investigate the relationship between droplet diameter and rotational speed, we first conducted droplets generation experiment with bench-top centrifuge under different rotational speeds. The results demonstrated a negative correlation between droplet diameter and centrifugal rotational speed; as the rotational speed increased, the droplet diameter decreased (Figure S23-28).


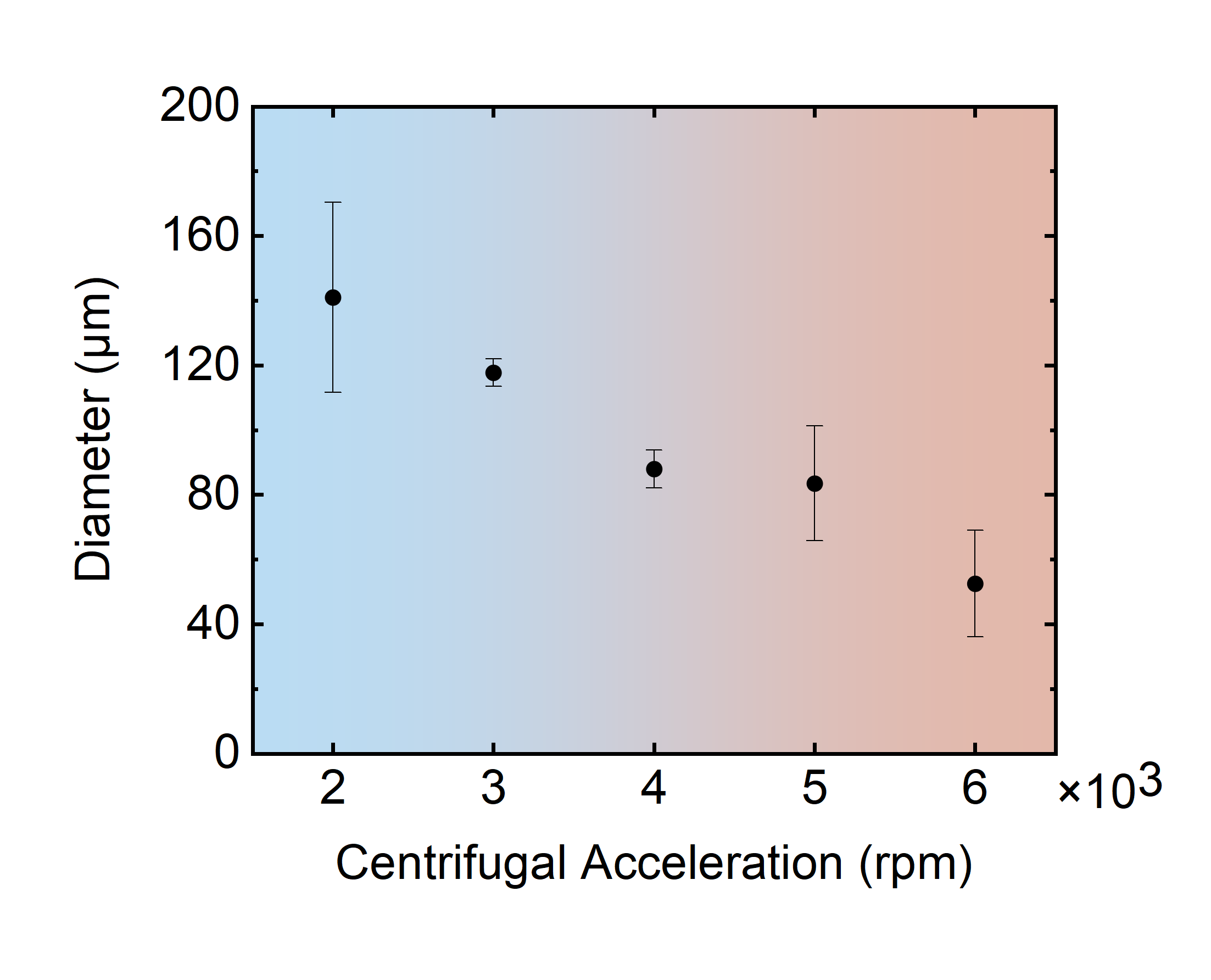


**Figure S23 | The relationship between droplet diameter and centrifuge speed.** The error bars representing the standard deviation of droplet diameter.

**Figure S24**


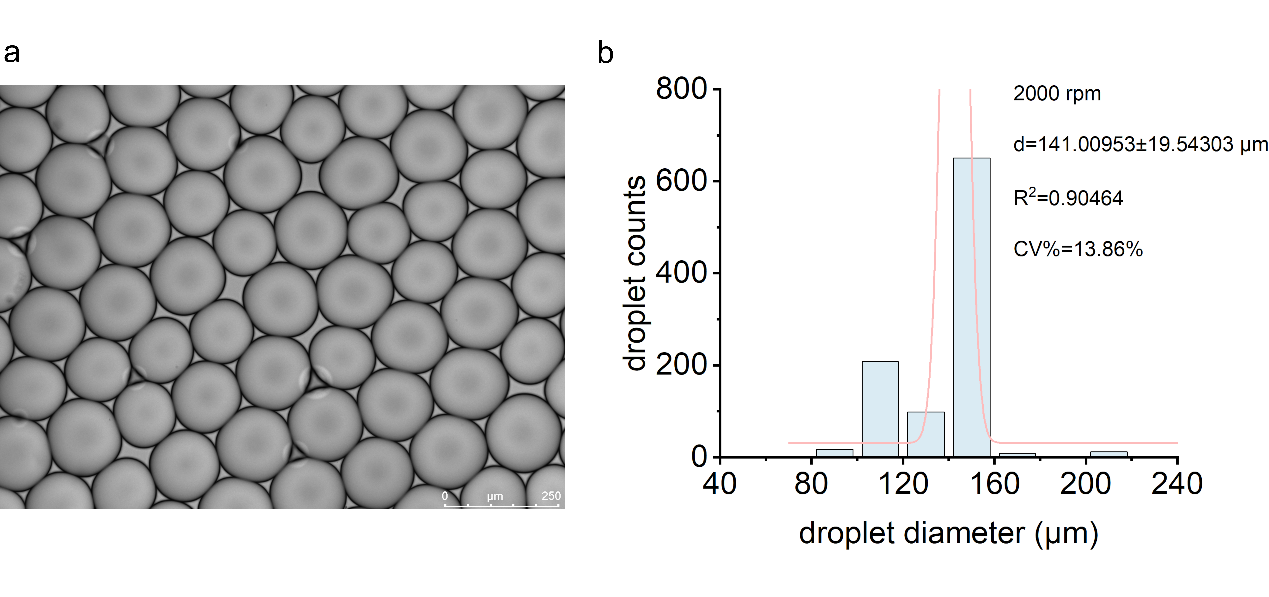


**Figure S24 | Droplet morphology and diameter at 2000 rpm.** (a) Droplet morphology under the bright field of a Leica DM4 B Upright Microscopes (10×). (b) Histogram of droplet diameter frequency distribution. CV% (coefficient of variation) =sd./mean × 100%.

**Figure S25**


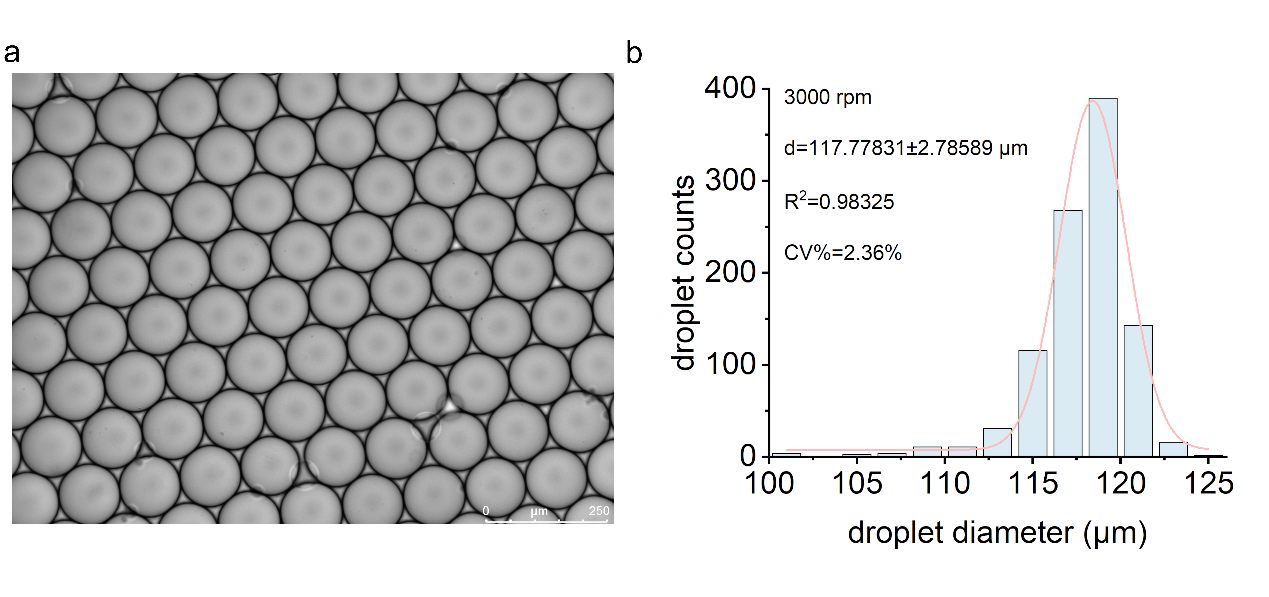


**Figure S25 | Droplet morphology and diameter at 3000 rpm.** (a) Droplet morphology under the bright field of a Leica DM4 B Upright Microscopes (10×). (b) Histogram of droplet diameter frequency distribution. CV% (coefficient of variation) =sd./mean × 100%.

**Figure S26**


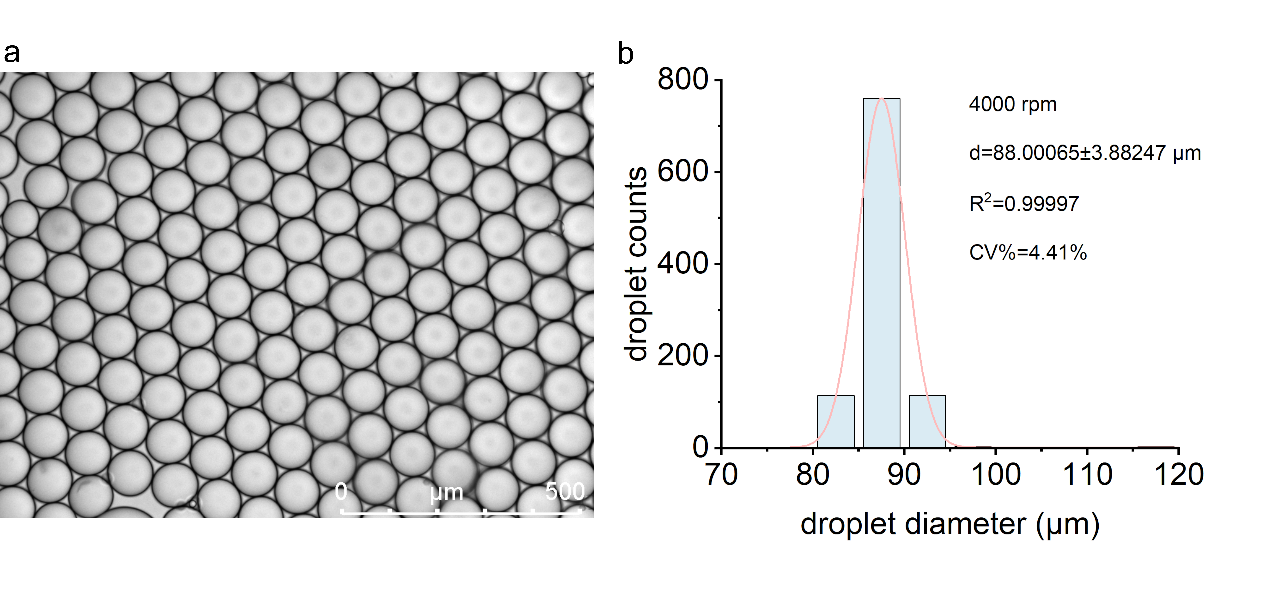


**Figure S26 | Droplet morphology and diameter at 4000 rpm.** (a) Droplet morphology under the bright field of a Leica DM4 B Upright Microscopes (10×). (b) Histogram of droplet diameter frequency distribution. CV% (coefficient of variation) =sd./mean × 100%.

**Figure S27**


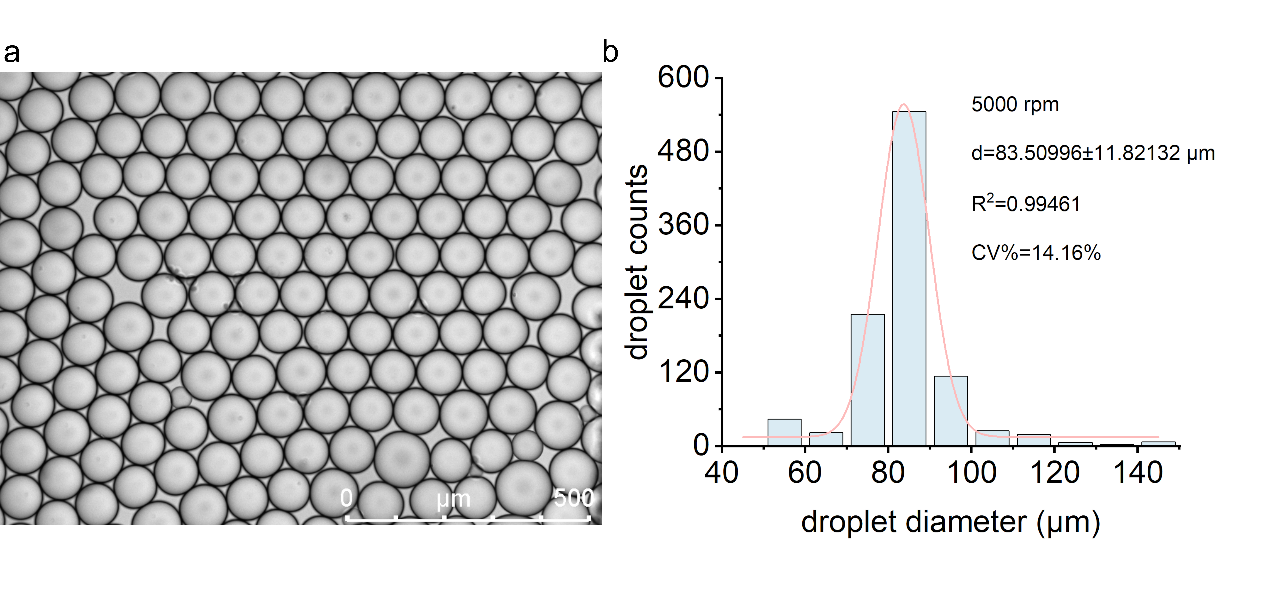


**Figure S27 | Droplet morphology and diameter at 5000 rpm.** (a) Droplet morphology under the bright field of a Leica DM4 B Upright Microscopes (10×). (b) Histogram of droplet diameter frequency distribution. CV% (coefficient of variation) =sd./mean × 100%.

**Figure S28**


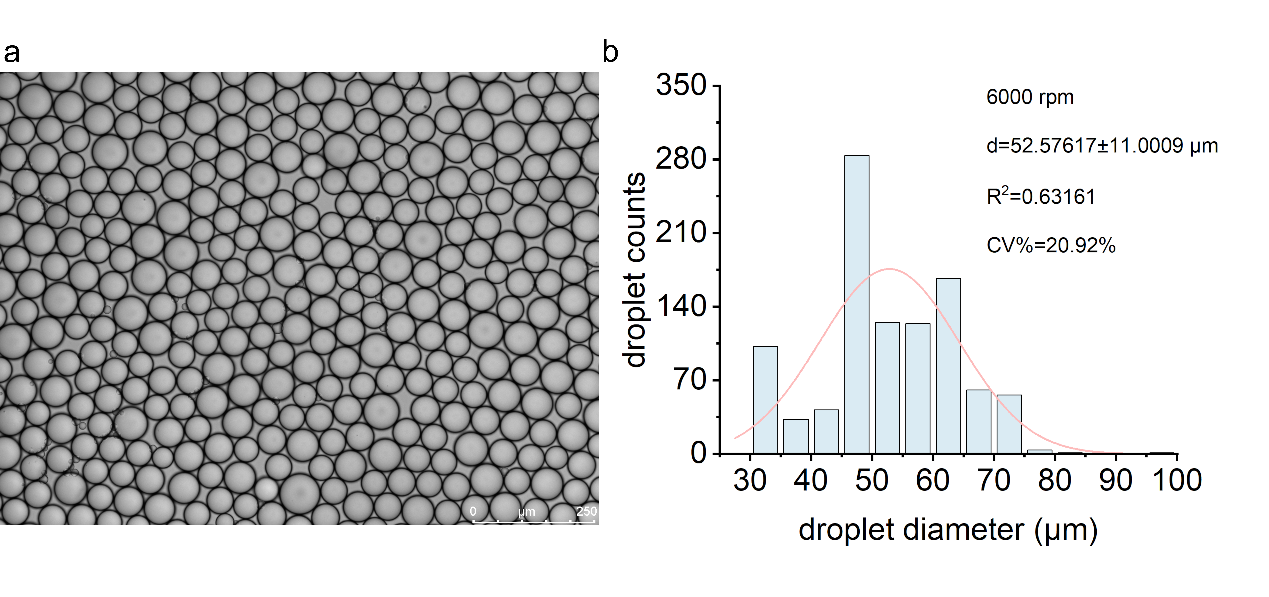


**Figure S28| Droplet morphology and diameter at 6000 rpm.** (a) Droplet morphology under the bright field of a Leica DM4 B Upright Microscopes (10×). (b) Histogram of droplet diameter frequency distribution. CV% (coefficient of variation) =sd./mean × 100%.

**Figure S29**


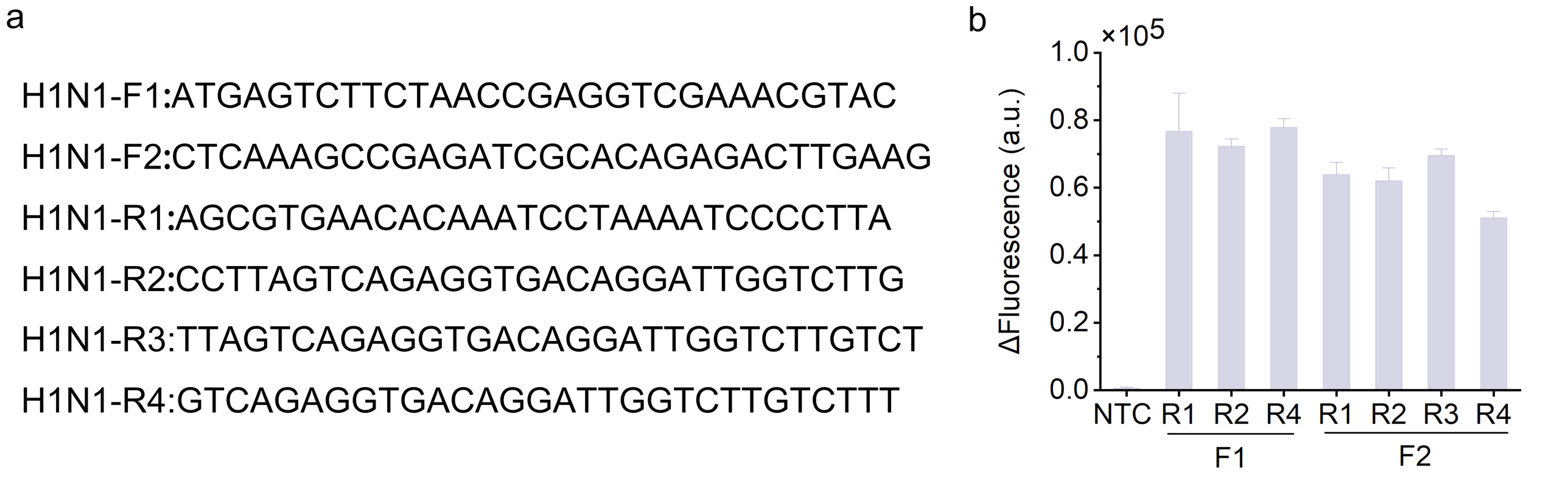


**Figure S29 | Primer screening of influenza A virus (H1N1).** The conventional RPA was performed using an H1N1 plasmid (1 pg/μL) with various primer pairs. The results indicated that the conventional RPA using the H1N1-F1 and H1N1-R4 primer pair exhibited the most pronounced fluorescent change. Therefore, we used H1N1-R4 to designed photocaged reverse primer (H1N1-RP4-NPOM) for Light-start RPA assay. F indicates the forward primer; R indicates the reverse primer. NTC represents the blank control, using RNase-free water instead of plasmid template. ΔFluorescence (a.u.) represents the difference between the fluorescence value and the initial fluorescence value. Data are represented as mean ± standard error (n = 2 technical replicates).

**Figure S30**


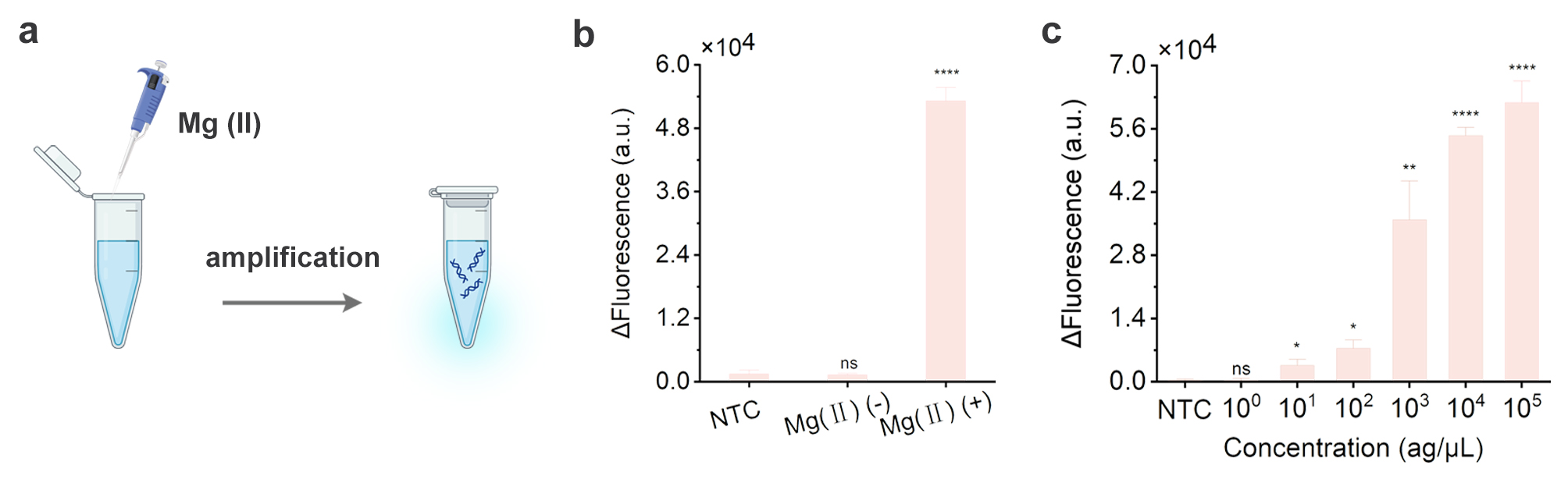


**Figure S30 | Conventional RPA system for the detection of influenza A virus (H1N1) plasmid.** (a) The operation flow diagram of a conventional RPA system. (b) Feasibility study on magnesium ions to initiate conventional RPA for detecting H1N1 plasmid (1 pg/μL). "Mg (II) (+)" indicates that the reaction with magnesium ion. "Mg (II) (-)" indicates that the reaction without magnesium ion. (c) Evaluation of the LoD of conventional RPA for detection of H1N1 plasmid templates. NTC represents the blank control, using RNase-free water instead of plasmid template. ΔFluorescence (a.u.) represents the difference between the fluorescence value and the initial fluorescence value. Fluorescence intensity was compared with NTC using a two-tailed t-test for significant differences: ****P < 0.0001; **P < 0.01; *P < 0.05; ns represents no significant difference from NTC. Data are represented as mean ± standard error (n = 3 technical replicates).

**Figure S31**





**Figure S31 | Primer screening of EMP1, ZIKV and** **CHIKV.** The conventional RPA was performed using EMP1 (a-b), CHIKV (c-d), or ZIKV (e-f) plasmids (1 pg/μL) with various primer pairs. The results indicated that the conventional RPA using the F2-R2 primer pair for EMP1 plasmids, the F3-R1 primer pair for CHIKV plasmids, and the F3-R3 primer pair for ZIKV plasmids exhibited the most pronounced fluorescent change. Therefore, we used the optimal reverse primers to designed photocaged reverse primers (EMP1-RP2-NPOM, CHIKV-RP1-NPOM, ZIKV-RP3-NPOM) for Light-start RPA. F indicates the forward primer; R indicates the reverse primer. NTC represents the blank control, using RNase-free water instead of plasmid template. ΔFluorescence (a.u.) represents the difference between the fluorescence value and the initial fluorescence value. Specifically, the Bio-Rad Connect qPCR instrument was used to monitor the fluorescent signal of the conventional RPA system with the EMP1 plasmid, while the ABI Q3 instrument was used to monitor the fluorescent signal of the conventional RPA system with the ZIKV and CHIKV plasmids. Data are represented as mean ± standard error (n = 2 technical replicates).

**Figure S32**


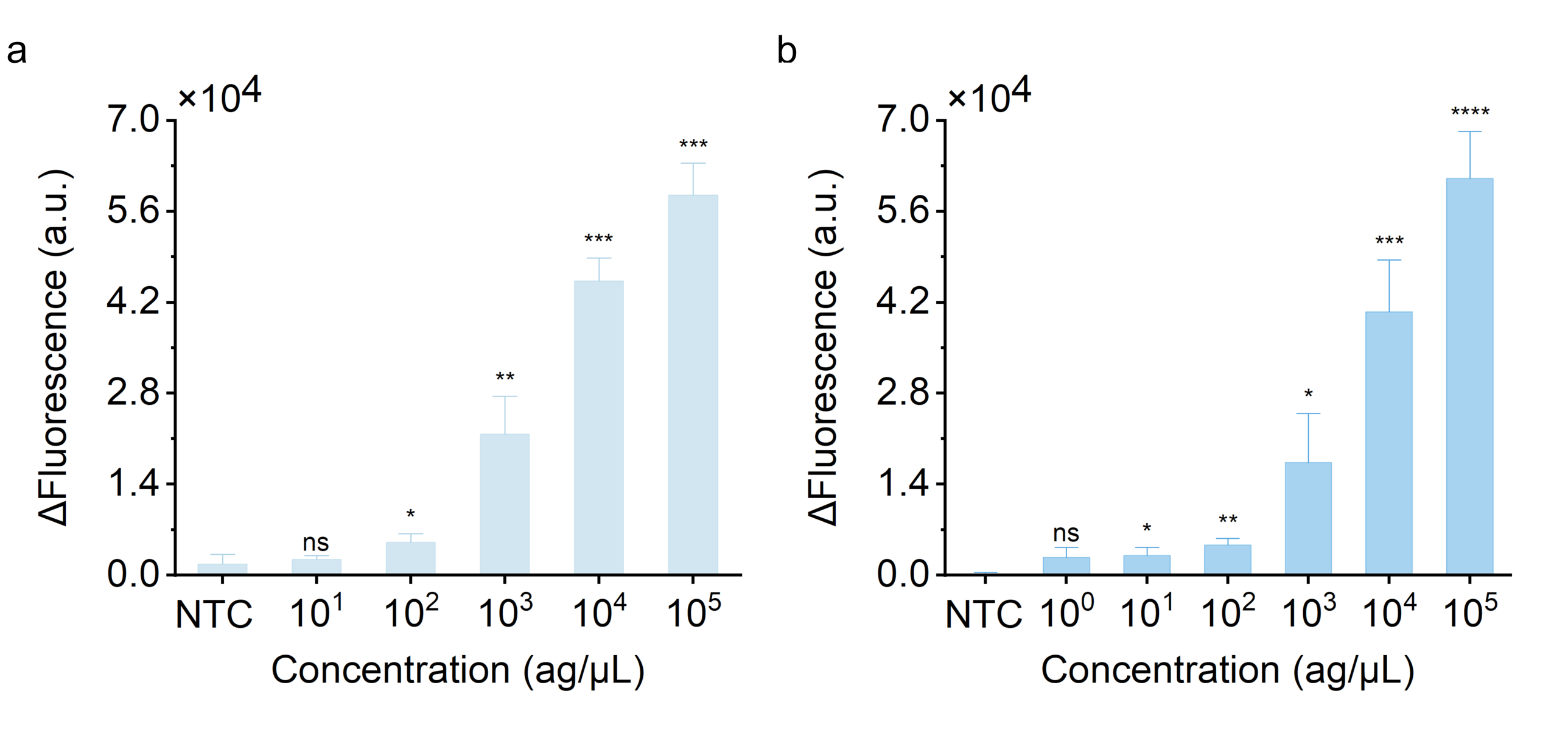


**Figure S32 |** **Comparison of conventional RPA and Light-start RPA for the detection of plasmodium falciparum (EMP1) plasmid.** (a) The conventional RPA with wild forward and reverse primers (EMP1-FP2 and EMP1-RP2) can detect synthesized EMP1 plasmids at concentrations as low as 100 ag/µL. (b) The Light-start RPA with EMP1-FP2 and EMP1-RP2-NPOM primers can detect synthesized EMP1 plasmids at concentrations as low as 10 ag/µL. NTC represents the blank control, using RNase-free water instead of plasmid template. ΔFluorescence (a.u.) represents the difference between the fluorescence value and the initial fluorescence value. Data are represented as mean ± standard error (n = 3 technical replicates).

**Figure S33**


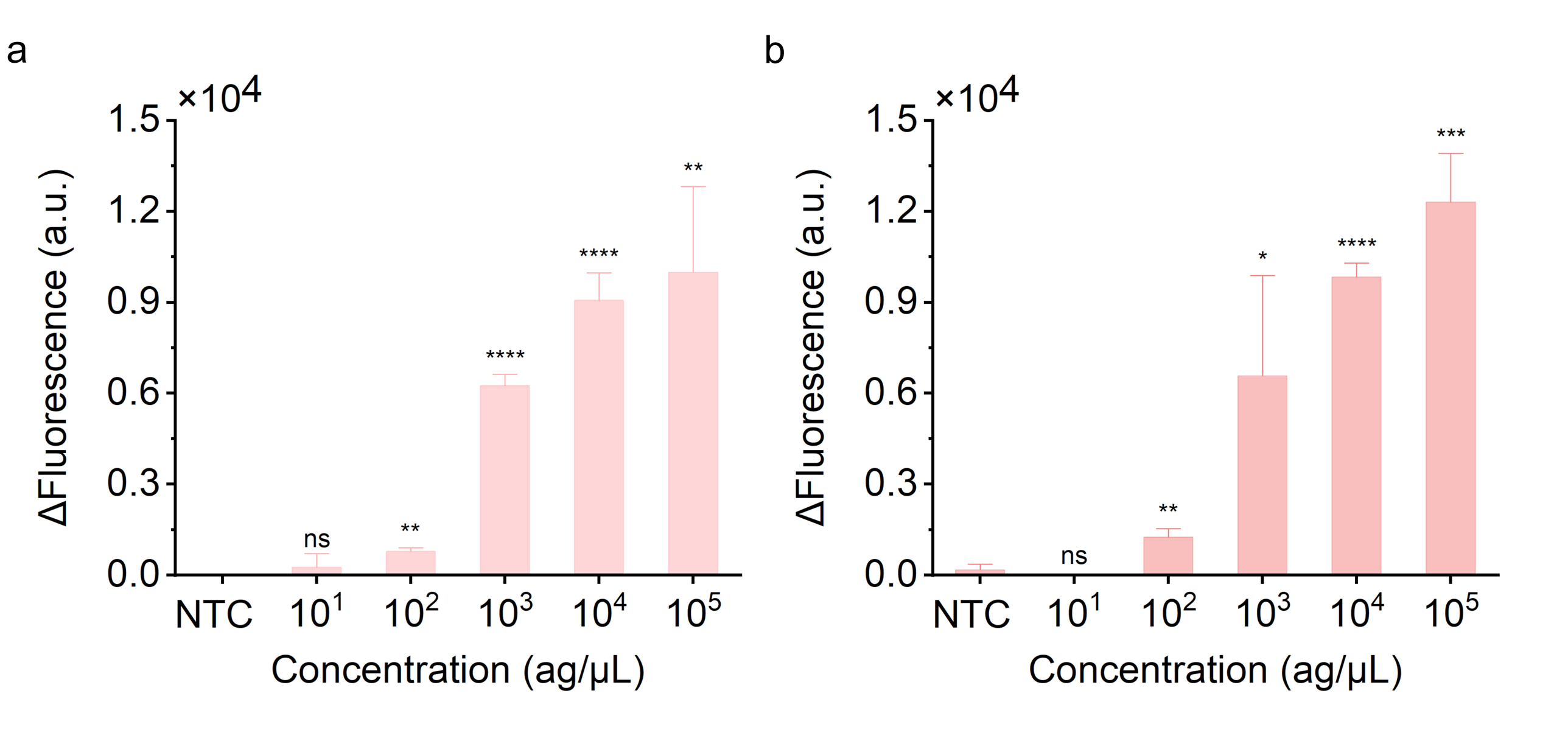


**Figure S33 | Comparison of conventional RPA and Light-start RPA for the detection of flavivirus dengue virus (DENV) plasmid.** (a) The conventional RPA with wild forward and reverse primers (DENV-FP and DENV-RP) can detect synthesized DENV plasmids at concentrations as low as 100 ag/µL. (b) The Light-start RPA with DENV-FP and DENV-RP-NPOM primers can detect synthesized DENV plasmids at concentrations as low as 100 ag/µL. NTC represents the blank control, using RNase-free water instead of plasmid template. ΔFluorescence (a.u.) represents the difference between the fluorescence value and the initial fluorescence value. Data are represented as mean ± standard error (n = 3 technical replicates).

**Figure S34**

**
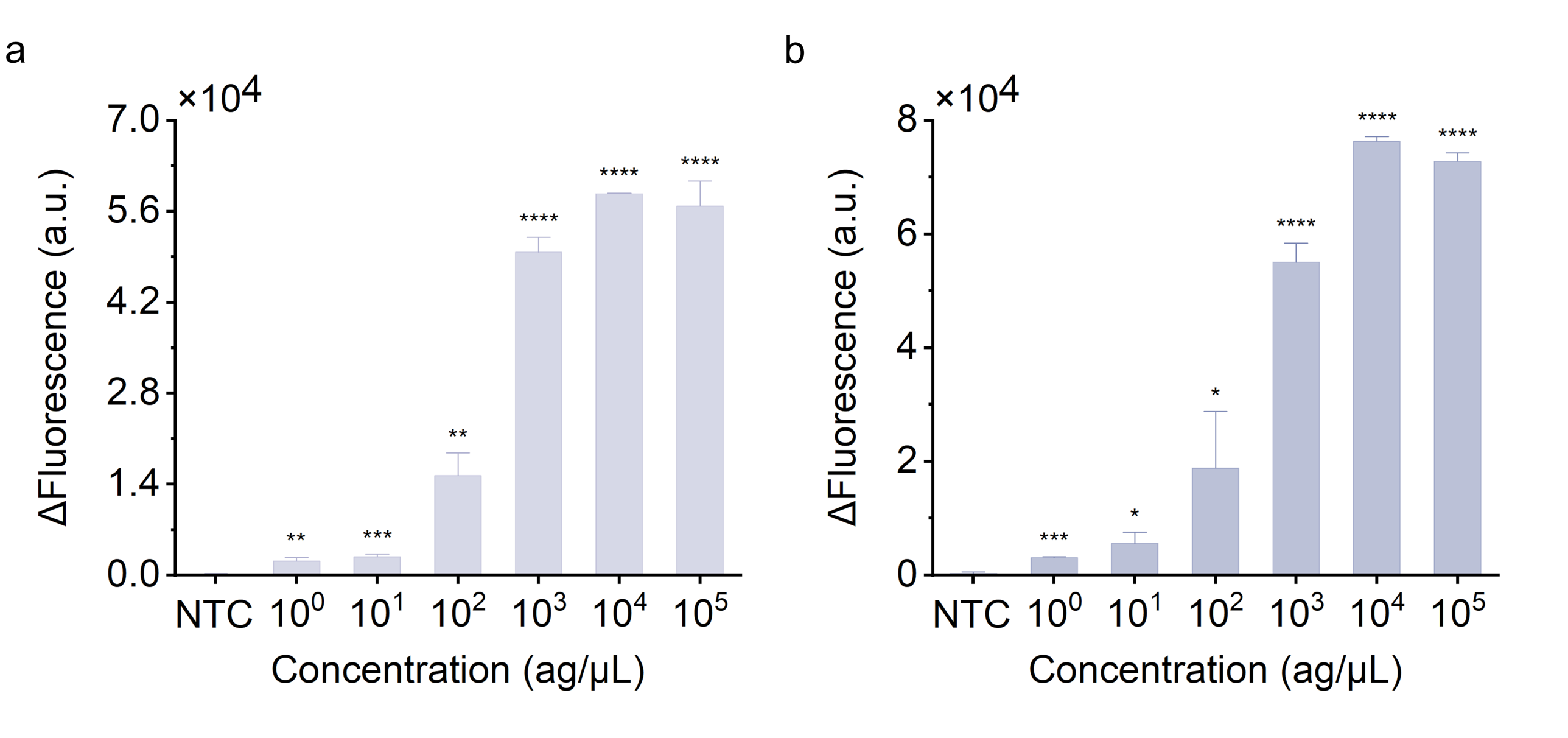
**

**Figure S34 | Comparison of conventional RPA and Light-start RPA for the detection of Chikungunya virus (CHIKV) plasmid.** (a) The conventional RPA with wild forward and reverse primers (CHIKV-FP3 and CHIKV-RP1) can detect synthesized CHIKV plasmids at concentrations as low as 1 ag/µL. (b) The Light-start RPA with CHIKV-FP3 and CHIKV-RP1-NPOM primers can detect synthesized CHIKV plasmids at concentrations as low as 1 ag/µL. NTC represents the blank control, using RNase-free water instead of plasmid template. ΔFluorescence (a.u.) represents the difference between the fluorescence value and the initial fluorescence value. Data are represented as mean ± standard error (n = 3 technical replicates).

**Figure S35**


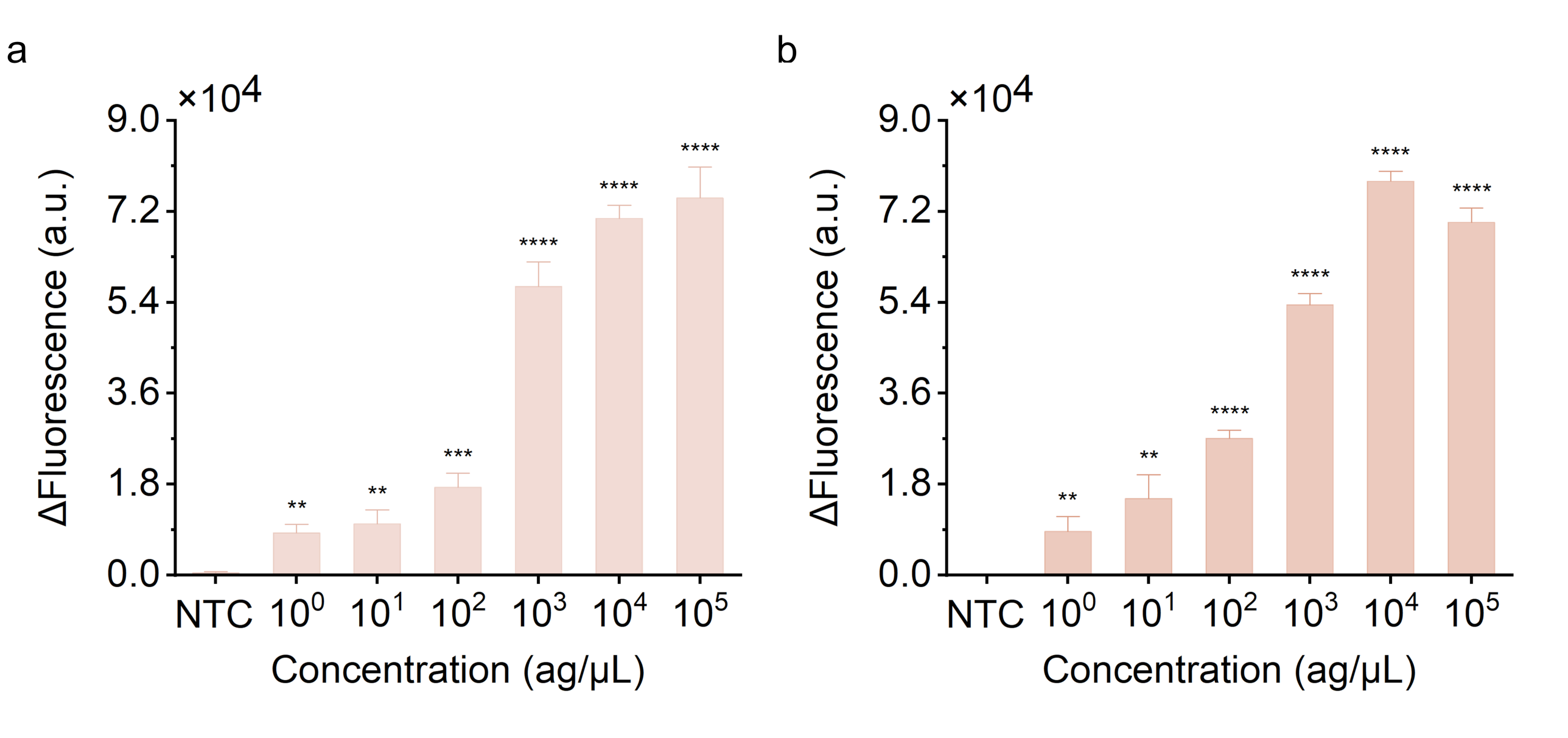


**Figure S35 | Comparison of conventional RPA and Light-start RPA for the detection of Zika virus (ZIKV) plasmid.** (a) The conventional RPA with wild forward and reverse primers (ZIKV-FP3 and ZIKV-RP3) can detect synthesized ZIKV plasmids at concentrations as low as 1 ag/µL. (b) The Light-start RPA with ZIKV-FP3 and ZIKV-RP3-NPOM primers can detect synthesized ZIKV plasmids at concentrations as low as 1 ag/µL. NTC represents the blank control, using RNase-free water instead of plasmid template. ΔFluorescence (a.u.) represents the difference between the fluorescence value and the initial fluorescence value. Data are represented as mean ± standard error (n = 3 technical replicates).

**Figure S36**


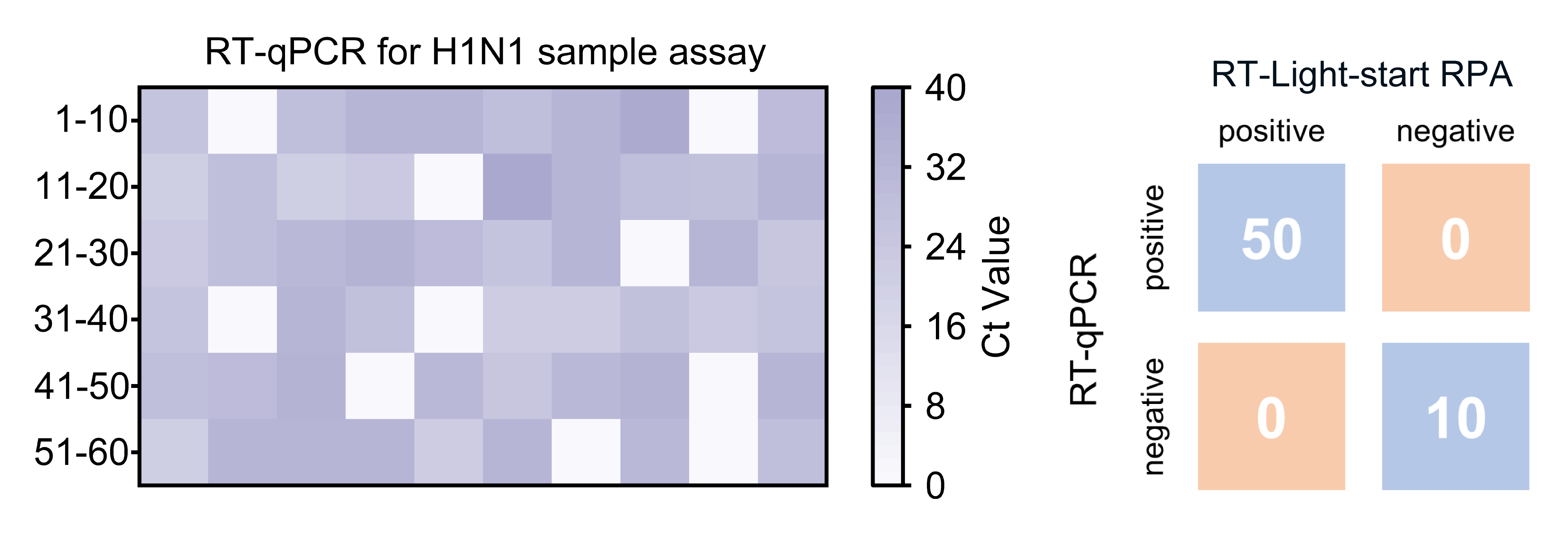


**Figure S36 | CFDA-approved RT-qPCR for detection of H1N1 RNA in clinical samples.** (a) The heat map showing the detection results of 60 clinical H1N1 RNA samples using CFDA-approved RT-qPCR method. (b) Correlation of RT-Light-start RPA with RT-qPCR detection in clinical samples.

**Figure S37**

We further evaluated the adaptability of Light-start RPA technology for instrument-free point-of-care diagnostics by integrating it with a lateral flow assay (RT-Light-start RPA-LFA). As shown in Figure S37, the RT-Light-start RPA-LFA probe (LFA-Probe) is designed using a sandwich method: a complementary sequence to the template is positioned between the upstream and downstream primers. The 5' end is modified with carboxyfluorescein (FAM), the middle region is marked with a dSpacer (THF), and the 3' end is labeled with a blocking group. When the amplicons are applied to the LFA strip, they bind to the Au NPs-labeled anti-FAM antibody, forming a ternary complex. As the complex flows through the test line (T), it is captured by streptavidin, producing a visible test line. When the complex continues to the control line (C), the Au NPs-labeled anti-FAM antibody binds to the secondary antibody, producing a visible control line. A positive result is indicated when both the test and control lines show color, while a negative result is indicated when only the control line is colored.


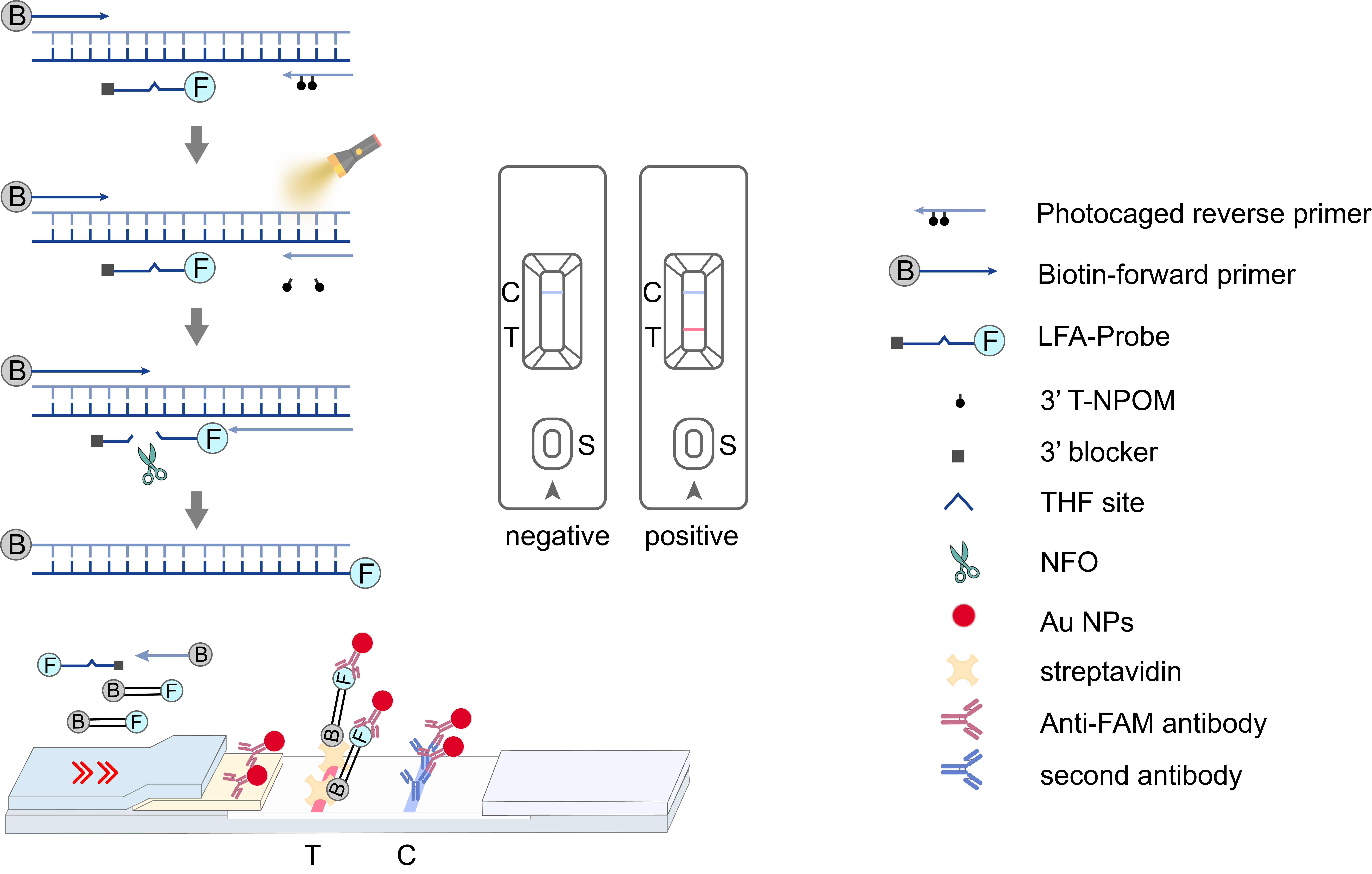


**Figure S37 | Schematic diagram of RT-Light-start RPA-LFA.**

**Figure S38**


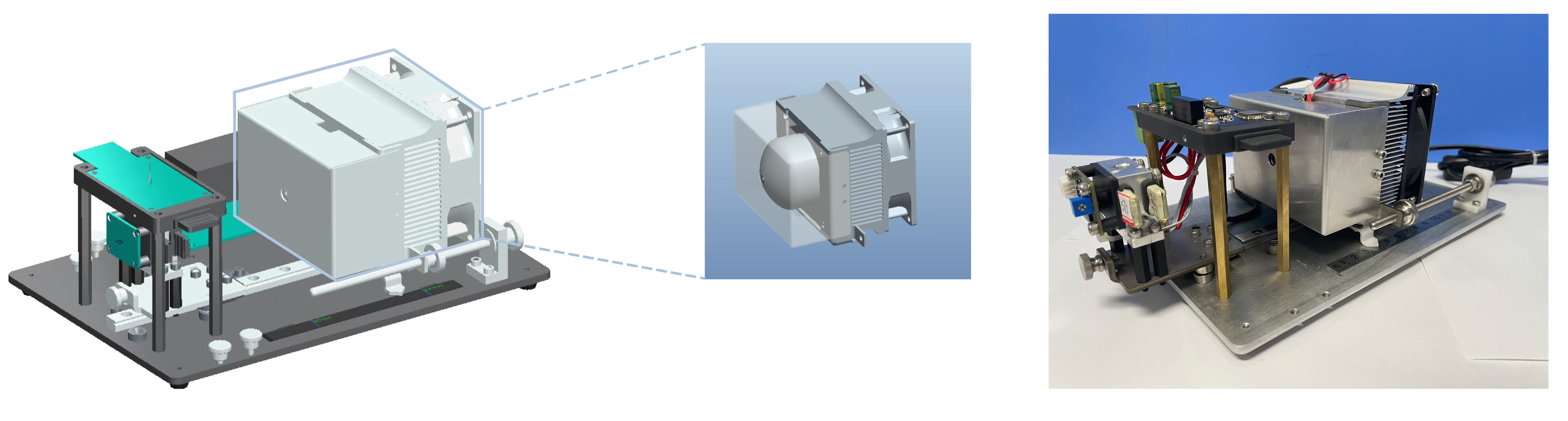


**Figure S38 | Three-dimensional models and illustrations of integrated Light-start RPA detection device.**
